# Alkynyl Ligands as Building Blocks for the Preparation of Phosphorescent Iridium(III) Emitters: Alternative Synthetic Precursors and Procedures

**DOI:** 10.1021/acs.inorgchem.2c00197

**Published:** 2022-04-19

**Authors:** Vadim Adamovich, María Benítez, Pierre-Luc Boudreault, María L. Buil, Miguel A. Esteruelas, Enrique Oñate, Jui-Yi Tsai

**Affiliations:** † Departamento de Química Inorgánica, Instituto de Síntesis Química y Catálisis Homogénea (ISQCH), Centro de Innovación en Química Avanzada (ORFEO-CINQA), Universidad de Zaragoza—CSIC, 50009 Zaragoza, Spain; ‡ Universal Display Corporation, Ewing, New Jersey 08618, United States

## Abstract

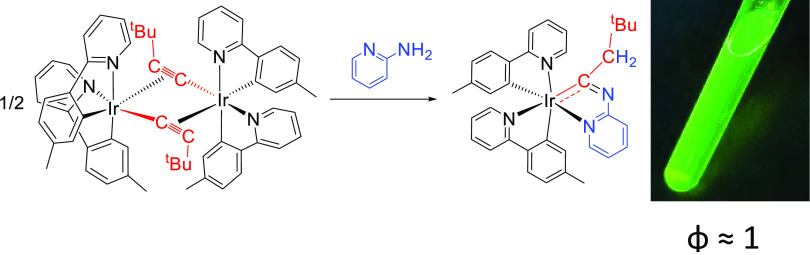

Alkynyl ligands stabilize dimers [Ir(μ-X)(3b)_2_]_2_ with a cis disposition of the heterocycles of the 3b ligands, in contrast to chloride. Thus, the complexes of this class—*cis-*[Ir(μ_2_-η^2^-C≡CPh){κ^2^-*C*,*N*-(C_6_H_4_-Isoqui)}_2_]_2_ (Isoqui = isoquinoline) and *cis-*[Ir(μ_2_-η^2^-C≡CR){κ^2^-*C*,*N*-(MeC_6_H_3_-py)}_2_]_2_ (R = Ph, *^t^*Bu)—have been prepared in high yields, starting from the dihydroxo-bridged dimers *trans-*[Ir(μ-OH){κ^2^-*C*,*N*-(C_6_H_4_-Isoqui)}_2_]_2_ and *trans-*[Ir(μ-OH){κ^2^-*C*,*N*-(MeC_6_H_3_-py)}_2_]_2_ and terminal alkynes. Subsequently, the acetylide ligands have been employed as building blocks to prepare the orange and green iridium(III) phosphorescent emitters, Ir{κ^2^-*C*,*N*-[C(CH_2_Ph)Npy]}{κ^2^-*C*,*N*-(C_6_H_4_-Isoqui)}_2_ and Ir{κ^2^-*C*,*N*-[C(CH_2_R)Npy]}{κ^2^-*C*,*N*-(MeC_6_H_3_-py)}_2_ (R = Ph, *^t^*Bu), respectively, with an octahedral structure of *fac* carbon and nitrogen atoms. The green emitter Ir{κ^2^-*C*,*N*-[C(CH_2_*^t^*Bu)Npy]}{κ^2^-*C*,*N*-(MeC_6_H_3_-py)}_2_ reaches 100% of quantum yield in both the poly(methyl methacrylate) (PMMA) film and 2-MeTHF at room temperature. In organic light-emitting diode (OLED) devices, it demonstrates very saturated green emission at a peak wavelength of 500 nm, with an external quantum efficiency (EQE) of over 12% or luminous efficacy of 30.7 cd/A.

## Abstract 2

Acetylide ligands have been employed as building blocks to prepare orange and green iridium(III) phosphorescent emitters, with an octahedral structure of *fac* carbon and nitrogen atoms. In OLED devices, the emitter Ir{κ^2^-*C*,*N*-[C(CH_2_*^t^*Bu)Npy]}{κ^2^-*C*,*N*-(MeC_6_H_3_-py)}_2_ demonstrates very saturated green emission at a peak wavelength of 500 nm, with a luminous efficacy of 30.7 cd/A.

## Introduction

There is great interest in iridium(III) phosphorescent emitters because they show a fast *S*_0_–*T*_1_ intersystem crossing. Such ability allows them to harvest singlet and triplet excitons and to achieve internal quantum efficiencies close to 100%.^1^ The highest occupied molecular orbital–lowest unoccupied molecular orbital (HOMO–LUMO) gap in these compounds depends upon the ligands, and therefore, it in principle appears to be possible to design compounds to obtain properties in accordance with the requirements of a given application.^2^ Thus, complexes bearing different ligands mobilize special attention since they facilitate a better fine tuning of the features of the emitter.^3^ Octahedral complexes coordinating three 3e-donor bidentate ligands (3b) are the most usual. Among them, species bearing two different types, [3b + 3b + 3b′], are particularly valued because they do not present the serious issues associated with the ligand distribution equilibria,^4^ which are observed for the heteroleptic emitters [3b + 3b′ + 3b″] containing three different ligands.^5^

The [3b + 3b + 3b′]-type emitters commonly contain two orthometalated phenyl-heterocycles (3b) and another ligand (3b′). Dimers [Ir(μ-Cl)(3b)_2_]_2_ are usually the starting point for the preparation of these compounds. In most of the cases, the synthesis procedure involves the replacement of the bridge chlorides by the own 3b′ ligand.^6^ Selective postfunctionalization of some coordinated ligands is an alternative procedure, which can be also successfully employed. It takes place in two steps, which include a C–H bromination and subsequently a palladium-catalyzed Suzuki–Miyaura cross-coupling.^7^ A third method scarcely used is the building of a new ligand on the metal coordination sphere by multicomponent reactions involving the coupling of several coordinated ligands or coordinated ligands and external molecules.^8^ At first glance, it is more challenging and requires the use of starting compounds other than the dimers [Ir(μ-Cl)(3b)_2_]_2_ or derivatives thereof.

A common structural feature of the emitters obtained by these procedures is the mutually trans disposition of the heterocyclic rings, with some rare exception observed with fluorinated phenyl-pyridines.^9^ This lack of structural diversity is a consequence of the retention of the stereochemistry of the mononuclear fragments of the dimers [Ir(μ-Cl)(3b)_2_]_2_ during the preparation reactions of the emitters. In the search for emitters with a cis disposition of the heterocycles, some linkers have been designed to tie them, but the rigidity of the resulting organic molecules greatly complicates the reactions usually employed for this type of synthesis.^10^ Thus, the stabilization of dimers [Ir(μ-X)(3b)_2_]_2_ with a cis disposition of the heterocycles of the 3b ligands is a target of prominent importance for the field.

A promising alternative to the chloride bridge is the alkynyl-type ligands, as chloride behave as monodentate 1e-donors in mononuclear compounds and bridge 3e-donors in bimetallic species.^11^ However, the metal–alkynyl bond is significantly more versatile than the metal–chloride. In contrast to chloride, the π-system of the C–C triple bond in principle provides a pathway for electron density delocalization. Thus, the alkynyl anions, isoelectronic with the carbonyl ligand, display moderate π-acceptor ability, which allows them to participate in metal-to-ligand back-bonding. Furthermore, the substituent of the C–C triple bond can govern the contribution of the σ-ligand-to-metal, π-metal-to-ligand, and π-ligand-to-metal bonding components to the metal–alkynyl bonding overall situation.^12^ Because the metal–heterocycle and metal–aryl bonds of the chelate chromophores provide an asymmetric bonding situation, such modifications in the metal–alkynyl interaction could be relevant to stabilize a particular disposition of the chelating chromophore. Moreover, an increase in the substituent size should destabilize the bimetallic unit, affording five-coordinate transitory fragments, which could provide pathways to change the mutual disposition of the rings and prevent the retention of the stereochemistry during the reactions of the dimers. An additional advantage of the alkynyl ligands is their potential use in organometallic synthesis as building blocks.^13^

**Scheme 1:**
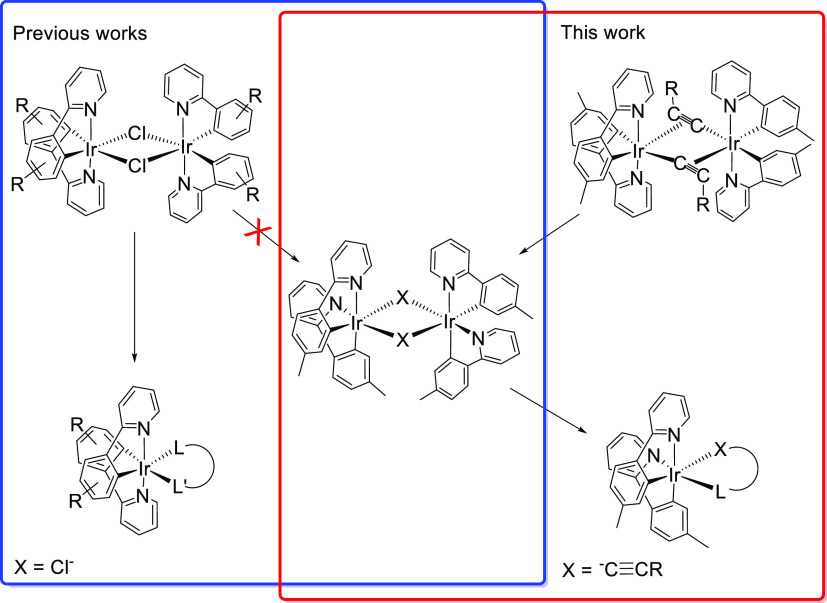
Contextualization of the Work

We are interested in finishing with the monotonous structures imposed by the dimers [Ir(μ-Cl)(3b)_2_]_2_. Thus, in the search for alternative starting materials, which would allow the preparation of emitters of the class [3b + 3b + 3b′] coordinating the 3b chromophores with their heterocycles cis-disposed, we have replaced the chloride bridges with acetylides. This paper demonstrates that in contrast to chloride, acetylide anions stabilize dimers [Ir(μ_2_-η^2^-C≡CR)(3b)_2_]_2_ coordinating the 3b ligands with the corresponding heterocycles in position cis, and such dimers allow to generate emitters [3b + 3b + 3b′], which retain the disposition, using the acetylide bridges as building blocks (Scheme 1).

## Results and Discussion

### [Ir(μ_2_-η^2^-C≡CR)(3b)_2_]_2_ Complexes Bearing Cis-Heterocycles

The acetylenic C(sp)–H bond is generally much more reactive than the C(sp^3^)–H and even C(sp^2^)–H bonds. Thus, it affords hydride–metal–alkynyl derivatives by oxidative addition to unsaturated transition metal complexes^14^ and generates metal–alkynyl species by heterolytic activation with saturated and unsaturated hydroxide compounds, where the OH group acts as an internal base.^15^ The C–H bond reactivity of the terminal alkynes and the ability of the hydroxide ligand to promote the abstraction of the acetylenic hydrogen atom, giving water as a unique subproduct, inspired us to use terminal alkynes and the dihydroxo-bridged dimers *trans-*[Ir(μ-OH){κ^2^-*C*,*N*-(C_6_H_4_-Isoqui)}_2_]_2_ (**1**) and *trans-*[Ir(μ-OH){κ^2^-*C*,*N*-(MeC_6_H_3_-py)}_2_]_2_ (**2**) as the precursor molecules to prepare the respective target acetylide dimers. Furthermore, the preparation of these dimers is very easy,^8e^ and their stability is comparable to that of the respective Cl dimers. The selected orthometalated 1-phenylisoquinoline ligand of dimer **1** should generate emitters in the low-energy region; it is well-known that the increase in the conjugation in the heterocycle by fused aromatic groups produces a red shift in the emission.^16^ In contrast, the orthometalated 2-(*p*-tolyl)pyridine chromophore would afford emitters in the zone of moderate–high energies.

**Scheme 2:**
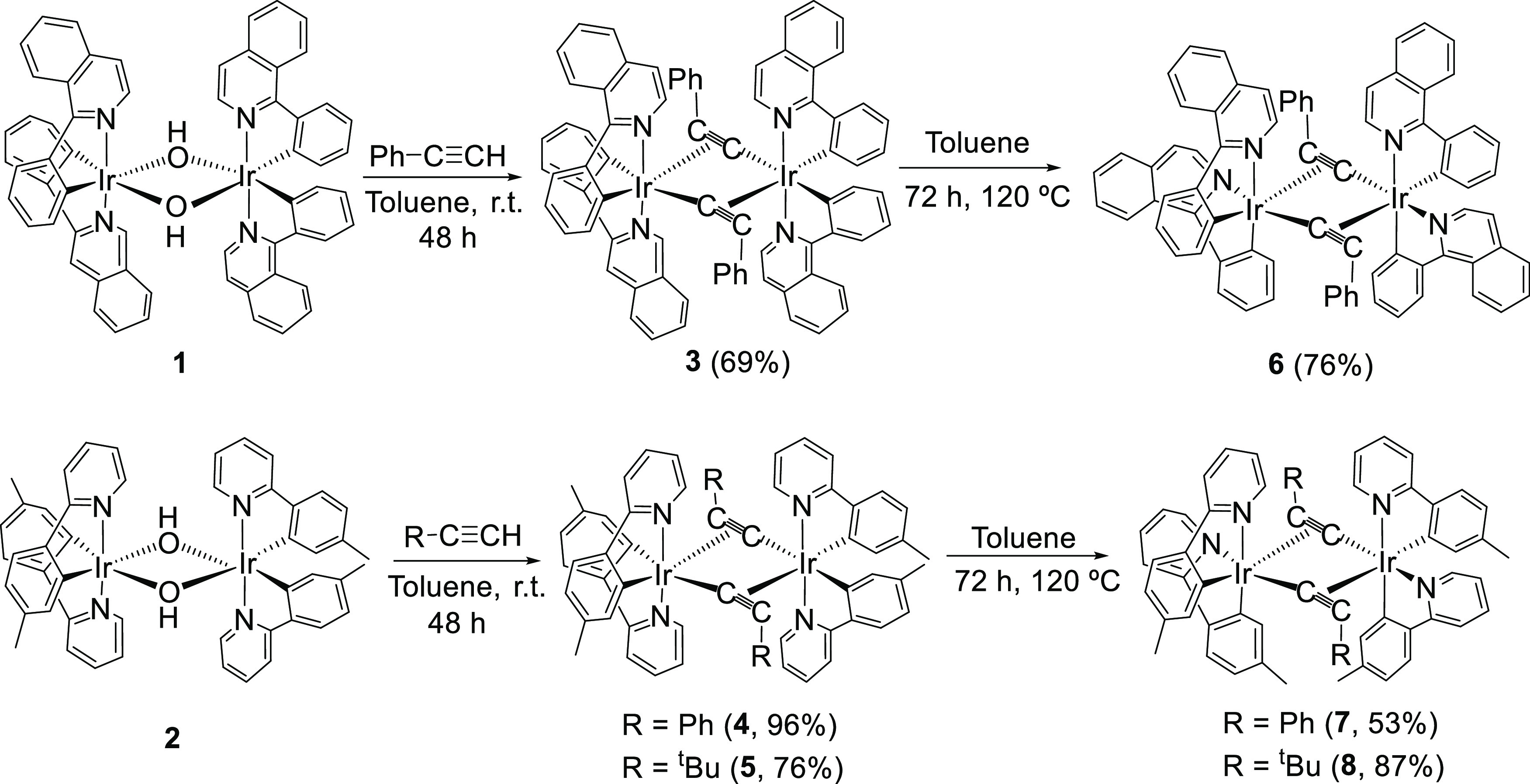
Preparation of Complexes **3**–**8**

Treatment of toluene suspensions of dimer **1** with 5.0 equiv of phenylacetylene and dimer **2** with 5.0 equiv of phenylacetylene and *tert*-butylacetylene, at room temperature, for 48 h leads to the dimers *trans-*[Ir(μ_2_-η^2^-C≡CPh){κ^2^-*C*,*N*-(C_6_H_4_-Isoqui)}_2_]_2_ (**3**) and *trans-*[Ir(μ_2_-η^2^-C≡CR){κ^2^-*C*,*N*-(MeC_6_H_3_-py)}_2_]_2_ (R = Ph (**4**), *^t^*Bu (**5**)), respectively, as a result of the OH-promoted heterolytic C(sp)–H bond activation of the respective terminal alkynes (Scheme 2). Complex **3** was obtained as a red solid in 69% yield, after Al_2_O_3_ column chromatography purification, whereas the *p*-tolylpyridine counterparts **4** and **5** were isolated as analytically pure-yellow solids in 96 and 73% yields, respectively, without the need for additional purification. In this context, we note that Lalinde and co-workers have prepared in moderate–good yields the related 2-phenylpyridine dimers *trans-*[Ir(μ_2_-η^2^-C≡CR){κ^2^-*C*,*N*-(C_6_H_4_-py)}_2_]_2_ (R = *p*-MeC_6_H_4_, *p*-MeOC_6_H_4_, 1-Np, *^t^*Bu, SiMe_3_), by alkynylation of the chloride precursor *trans-*[Ir(μ-Cl){κ^2^-*C*,*N*-(C_6_H_4_-py)}_2_]_2_ with the corresponding LiC≡CR or by displacement of acetonitrile from the mononuclear solvento cation [Ir{κ^2^-*C*,*N*-(MeC_6_H_3_-py)}_2_(CH_3_CN)_2_]^+^ with the acetylide.^17^

**Figure 1:**
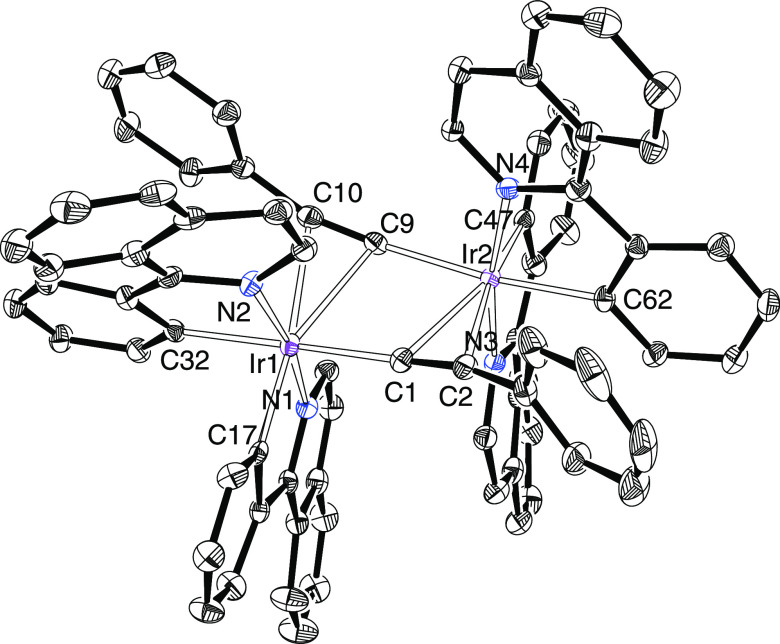
Oak Ridge Thermal Ellipsoid Plot (ORTEP) diagram of complex **3**. Hydrogen atoms are omitted for clarity. Selected bond lengths (Å) and angles (deg): Ir(1)–N(1) = 2.041(4), Ir(1)–N(2) = 2.052(4), Ir(1)–C(17) = 2.007(4), Ir(1)–C(32) = 2.046(4), Ir(1)–C(1) = 2.061(4), Ir(1)–C(9) = 2.445(4), Ir(1)–C(10) = 2.419(4), Ir(2)–N(3) = 2.046(4), Ir(2)–N(4) = 2.059(4), Ir(2)–C(47) = 2.001(4), Ir(2)–C(62) = 2.047(4), Ir(2)–C(9) = 2.097(4), Ir(2)–C(1) = 2.424(4), Ir(2)–C(2) = 2.464(4), C(1)–C(2) = 1.231(6), C(9)–C(10) = 1.231(6), N(1)–Ir(1)–N(2) = 170.08(14), C(32)–Ir(1)–C(1) = 168.35(17), N(3)–Ir(2)–N(4) = 169.36(14), and C(62)–Ir(2)–C(9) = 170.67(17).

**Figure 2:**
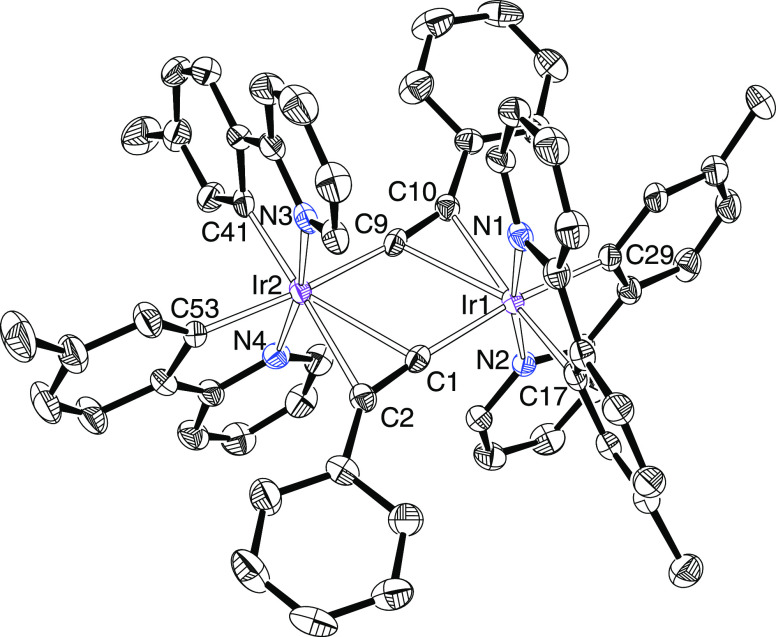
ORTEP diagram of complex **4**. Hydrogen atoms are omitted for clarity. Selected bond lengths (Å) and angles (deg): Ir(1)–N(1) = 2.048(3), Ir(1)–N(2) = 2.049(3), Ir(1)–C(17) = 2.015(4), Ir(1)–C(29) = 2.054(4), Ir(1)–C(1) = 2.055(4), Ir(1)–C(9) = 2.435(4), Ir(1)–C(10) = 2.379(4), Ir(2)–N(3) = 2.057(3), Ir(2)–N(4) = 2.051(3), Ir(2)–C(41) = 2.008(4), Ir(2)–C(53) = 2.054(4), Ir(2)–C(9) = 2.065(4), Ir(2)–C(1) = 2.424(4), Ir(2)–C(2) = 2.363(4), C(1)–C(2) = 1.243(5), C(9)–C(10) = 1.241(5), N(1)–Ir(1)–N(2) = 170.61(12), C(29)–Ir(1)–C(1) = 173.54(15), N(3)–Ir(2)–N(4) = 170.22(13), and C(53)–Ir(2)–C(9) = 172.41(15).

Complexes **3** and **4** were characterized by X-ray diffraction analysis. Both structures demonstrate the success of the C(sp)–H bond heterolytic activation, which takes place with total retention of the stereochemistry of the dimer precursors; the metal centers retain the cis disposition of the metalated phenyl groups and the trans disposition of the heterocycles, keeping the perpendicular chelate ligands in two groups of parallel planes. Previous density functional theory (DFT) calculations on the dihydroxo-bridged precursor **1** have revealed that this enantiomeric disposition is slightly more stable than a meso form.^8e^Figure 1 shows the structure of **3**, whereas Figure 2 gives a view of **4**. The polyhedron around each metal center is the typical octahedron for a six-coordinate d^6^-ion, with the alkynyl bridge ligands bonded through the terminal carbon atom to a metal center and by the C–C triple bond to the other. The metal–alkynyl distances are in the usual range and compare well with those reported for Lalinde’s compounds,^17^ whereas the metal–phenyl bond lengths point out a marked difference in trans-influence between the terminal carbon atom of the alkynyl ligand and its triple bond. Thus, in both structures, the Ir–C distances trans to the triple bond are about 0.04 Å shorter than the Ir–C bond lengths trans to the terminal carbon atom. In agreement with the presence of the alkynyl ligands in these complexes, their ^13^C{H} NMR spectra, at room temperature, in dichloromethane-*d*_2_ contain two singlets, one of them between 102 and 115 ppm and the other between 70 and 80 ppm, due to the C_α_ and C_β_ sp-atoms, respectively. It should be also mentioned that the ^1^H and ^13^C{^1^H} spectra of **4** and **5** furthermore reveal that the iridium centers exchange the C_β_ atoms of the alkynyl ligands. Thus, they display only one resonance for the two inequivalent pairs of methyl groups of the orthometalated *p*-tolyl substituents, at around 1.9 ppm in the ^1^H and at about 22 ppm in the ^13^C{^1^H}.

**Figure 3:**
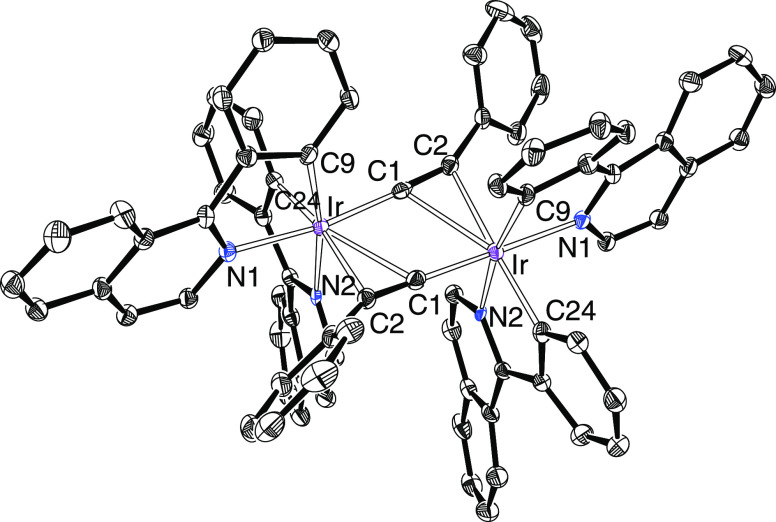
ORTEP diagram of complex **6**. Hydrogen atoms are omitted for clarity. Selected bond lengths (Å) and angles (deg): Ir–N(1) = 2.103(3), Ir–N(2) = 2.130(3), Ir–C(9) = 2.015(3), Ir–C(24) = 2.016(3), Ir–C(1) = 1.989(3), Ir–C(1) = 2.439(3), Ir–C(2) = 2.349(3), C(1)–C(2) = 1.229(5), C(1)–Ir–N(1) = 171.14(12), C(9)–Ir–N(2) = 170.37(12).

**Figure 4:**
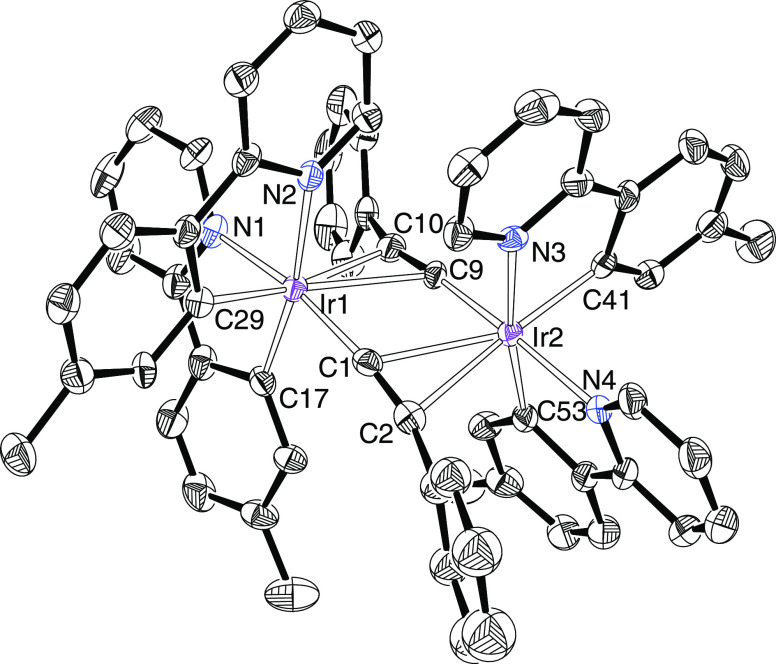
ORTEP diagram of complex **7**. Hydrogen atoms are omitted for clarity. Selected bond lengths (Å) and angles (deg): Ir(1)–N(1) = 2.108(5), Ir(1)–N(2) = 2.158(5), Ir(1)–C(17) = 2.018(6), Ir(1)–C(29) = 2.012(7), Ir(1)–C(1) = 1.989(7), Ir(1)–C(9) = 2.439(6), Ir(1)–C(10) = 2.370(7), Ir(2)–N(3) = 2.155(5), Ir(2)–N(4) = 2.099(5), Ir(2)–C(41) = 2.012(7), Ir(2)–C(53) = 2.006(6), Ir(2)–C(9) = 1.980(6), Ir(2)–C(1) = 2.418(7), Ir(2)–C(2) = 2.366(7), C(1)–C(2) = 1.216(10), C(9)–C(10) = 1.228(9), C(1)–Ir(1)–N(1) = 170.5(2), C(17)–Ir(1)–N(2) = 170.5(2), C(53)–Ir(2)–N(3) = 171.5(2), and C(9)–Ir(2)–N(4) =170.5(2).

There are noticeable differences in behavior between the acetylide dimers **3**–**5** and their precursors **1** and **2** and the chloride counterparts. In contrast to **1** and **2** and the chloride dimers, the mononuclear fragments of **3**–**5** isomerize in toluene, at 120 °C, changing the relative positions of one of the chelates. The isomerization gives rise to the strongly desired dimers *cis-*[Ir(μ_2_-η^2^-C≡CPh){κ^2^-*C*,*N*-(C_6_H_4_-Isoqui)}_2_]_2_ (**6**) and *cis-*[Ir(μ_2_-η^2^-C≡CR){κ^2^-*C*,*N*-(MeC_6_H_3_-py)}_2_]_2_ (R = Ph (**7**), *^t^*Bu (**8**)), bearing cis-heterocycles (Scheme 2). After 72 h, the transformation is quantitative. As a consequence, complexes **6**–**8** were isolated as analytically pure orange (**6**) or yellow (**7** and **8**) solids in high yields (53–87%). The X-ray diffraction analysis structures of **6** and **7** without a shadow of doubt demonstrate the isomerization and therefore the existence of dimers [Ir(μ-X)(3b)_2_]_2_, with a cis disposition of the heterocycles of the 3b ligands, when the bridge ligand X is an acetylide group. Figure 3 shows the structure of the isoquinoline derivative **6**, whereas Figure 4 shows the structure of the pyridine counterpart. In **3** and **4**, the orthometalated ligands lie in two groups of parallel planes. In addition to the heterocycle-phenyl trans disposition in both mononuclear fragments, the most noticeable feature of the structures is the disposition of the acetylide bridges. Located in a perpendicular plane to the N–Ir–C_phenyl_ directions, they dispose the terminal carbon atom trans to the remaining heterocycles, whereas the triple bond lies trans to the phenyl groups. The iridium–alkynyl distances and the iridium–phenyl bond lengths compare well with those of the isomeric precursors. In contrast to **3**–**5**, the structures of the dimers **6**–**8** are rigid in solution. Consistent with Figure 4, the NMR spectra of **7** and **8**, at room temperature, in dichloromethane-*d*_2_ display two singlets assigned to the methyl groups of the *p*-tolyl substituents at about 1.9 and 2.3 ppm in the ^1^H and between 21 and 22 ppm in the ^13^C{^1^H}. The ^13^C{^1^H} spectra also contain the signals due to the C_α_ and C_β_ sp-atoms of the alkynyl bridges, which are observed between 103 and 92 ppm and at about 72 ppm, respectively.

### Alkynyl Bridges as Building Blocks for the Preparation of New Chelating C,N-ligands

We reasoned that dimers **6–8** should be the entry to novel families of emitter compounds, bearing the heterocycles of the chromophores mutually cis-disposed, since the coordination of the acetylide anions to the iridium centers would produce an increase in the reactivity of the alkynyl triple bond, as a consequence of the nucleophilicity transfer from C_α_ to C_β_. Thus, the C–C triple bond should be susceptible to add electrophiles to C_β_ and nucleophiles to C_α_. As a concept validation proof, we decided to study the reactions of dimers **6**–**8** with 2-aminopyridine that has 2(1*H*)-pyridinimine as an imino tautomer.^18^

**Scheme 3:**
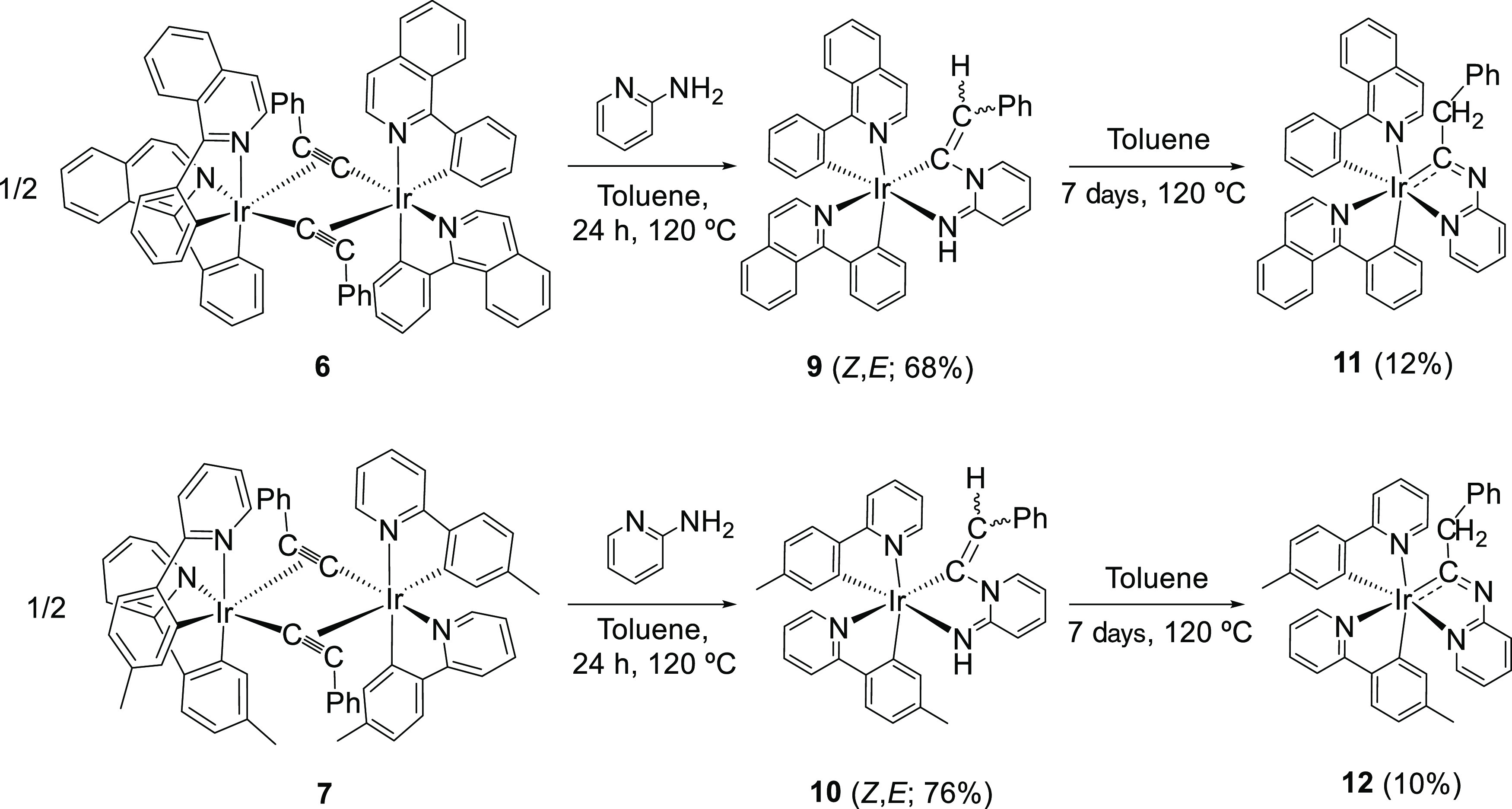
Preparation of Complexes **9**–**12**

Addition of 1.5 equiv of the amine to solutions of **6** and **7** in toluene at 120 °C leads to the mononuclear derivatives Ir{κ^2^-*C*,*N*-[C(=CHPh)-py-NH]}{κ^2^-*C*,*N*-(C_6_H_4_-Isoqui)}_2_ (**9**) and Ir{κ^2^-*C*,*N*-[C(=CHPh)-py-NH]}{κ^2^-*C*,*N*-(MeC_6_H_3_-py)}_2_ (**10**), after 24 h, as a result of the cleavage of the bridges of the dimer precursors, the addition of the N–H bond of the heterocycle of the imino tautomer of the *N*-reagent to the C–C triple bond of the acetylide ligands, and the coordination of the exocyclic imino group to the iridium centers. Complexes **9** and **10** were obtained as red and orange solids in 68 and 76% yields, respectively (Scheme 3).

**Figure 5:**
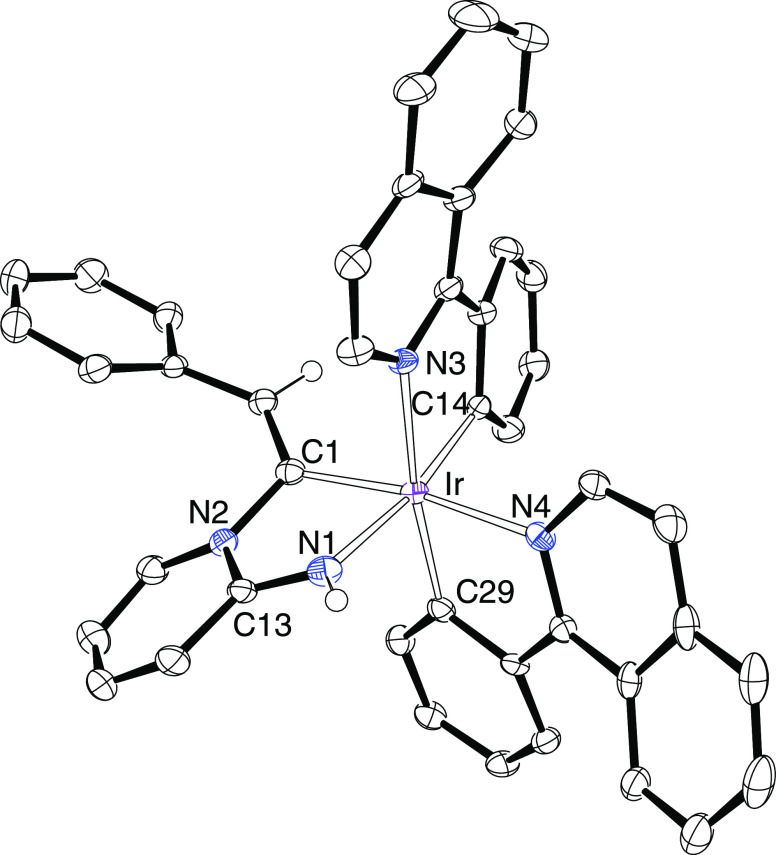
ORTEP diagram of complex **9**. Only significant hydrogen atoms are shown for clarity. Selected bond lengths (Å) and angles (deg): Ir–N(1) = 2.113(3), Ir–N(3) = 2.117(3), Ir–N(4) = 2.110(3), Ir–C(1) = 2.008(4), Ir–C(14) = 2.014(4), Ir–C(29) = 2.006(4), N(1)–C(13) = 1.313(5), N(2)–C(13) = 1.387(5), N(2)–C(1) = 1.464(5).

**Figure 6:**
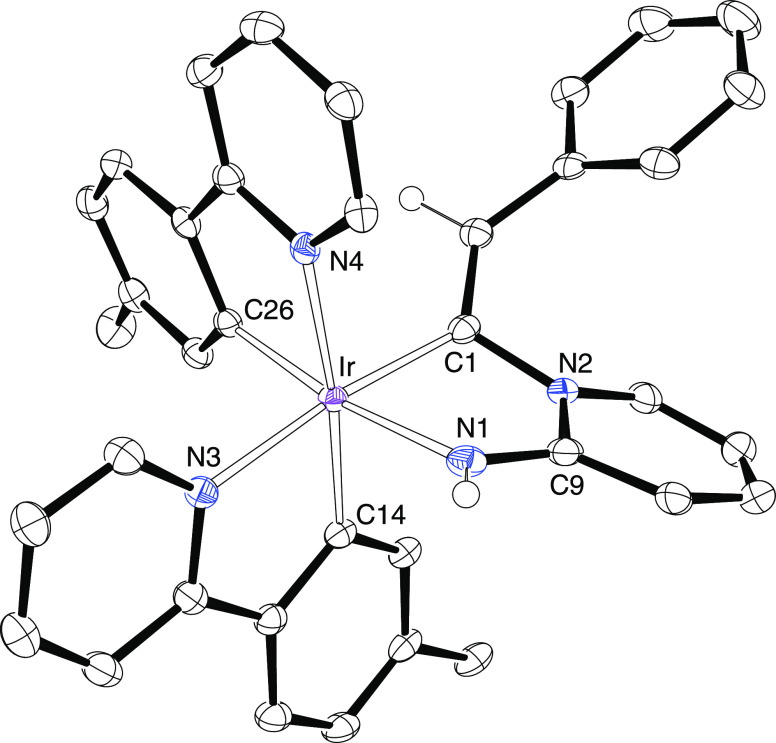
ORTEP diagram of complex **10**. Only significant hydrogen atoms are shown for clarity. Selected bond lengths (Å) and angles (deg): Ir–N(1) = 2.133(2), Ir–N(3) = 2.115(2), Ir–N(4) = 2.126(2), Ir–C(1) = 2.000(2), Ir–C(14) = 2.012(2), Ir–C(26) = 2.012(2), N(1)–C(29) = 1.308(3), N(2)–C(9) = 1.378(3), N(2)–C(1) = 1.470(3), C(26)–Ir–N(1) = 171.54(9), C(1)–Ir–N(3) = 172.86(9), and C(14)–Ir–N(4) = 173.58(9).

Complexes **9** and **10** were characterized by X-ray diffraction analysis. Figure 5 gives a view of the structure of the isoquinoline derivative **9**, whereas Figure 6 shows the structure of the pyridine complex **10**. Both structures prove the addition of the 2(1*H*)-pyridinimine tautomer to the triple bonds of the dimer precursors. The reactions give rise to a 3e-donor C,N-chelating styrylpyridinimine ligand. Thus, the polyhedron around the metal centers can be idealized as octahedrons defined by three C,N-chelating ligands with *fac* dispositions of carbons and heteroatoms. The most remarkable characteristic of the generated ligand is the *E*-stereochemistry of the styryl moiety, with the hydrogen atom pointing out the electron cloud of the orthometalated substituent of one of the heterocycles and the metal fragment and the phenyl group trans-disposed with regard to the C–C double bond. The ^1^H and ^13^C{^1^H} NMR spectra, at room temperature, in dichloromethane-*d*_2_ reveal that in solution, these compounds exist as a mixture of *E*- and *Z*-styryl isomers, in about 3:2 molar ratio. Thus, the ^1^H spectra display two broad singlets at about 5.8 and 5.4 ppm due to the NH-hydrogen atom of the imine moiety, whereas the signals due to the CHPh-hydrogen atom are observed at 6.43 (**9**) and 6.67 (**10**) ppm for an isomer and around 4.9 ppm for the other. We assume that isomer *E* is the major one in both cases since it has lower steric hindrance and its styryl CHPh resonance appears at higher field as a consequence of the ring current effect. In the ^13^C{^1^H} spectra, the resonances corresponding to the endocyclic carbon atom of the styryl moiety appear close to 150 ppm for both isomers of both complexes.

The styrylpyridinimine ligand of **9** and **10** rearranges to give an iridaimidazo[1,2-*a*]pyridine bicycle, in toluene, at 120 °C. The transformation is slow and partial. Thus, under the above-mentioned conditions, complexes **9** and **10** evolve to the iridaimidazopyridine derivatives Ir{κ^2^-*C*,*N*-[C(CH_2_Ph)Npy]}{κ^2^-*C*,*N*-(C_6_H_4_-Isoqui)}_2_ (**11**) and Ir{κ^2^-*C*,*N*-[C(CH_2_Ph)Npy]}{κ^2^-*C*,*N*-(MeC_6_H_3_-py)}_2_ (**12**), to afford a mixture of both classes of constitutional isomers, in about 7:3 molar ratio, after a week (Scheme 3). Complexes **11** and **12** were separated from the mixture by silica column chromatography and isolated as orange and yellow solids, respectively, in about 10% yield.

**Figure 7:**
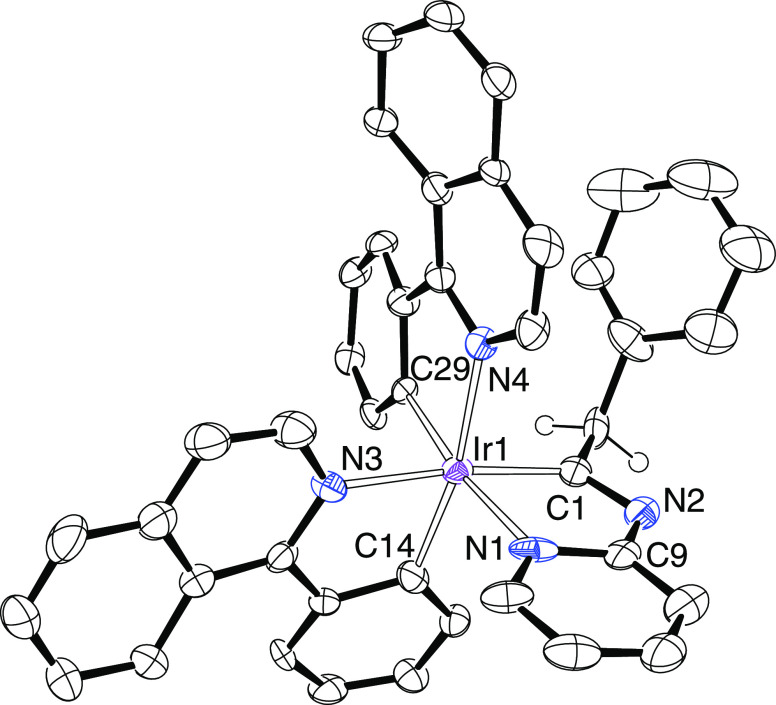
ORTEP diagram of complex **11**. Only significant hydrogen atoms are shown for clarity. Selected bond lengths (Å) and angles (deg): Ir(1)–N(1) = 2.126(7), 2.105(7); Ir(1)–N(3) = 2.139(7), 2.151(7); Ir(1)–N(4) = 2.125(7), 2.150(7); Ir(1)–C(1) = 1.992(10), 1.998(9); Ir(1)–C(14) = 2.011(9), 2.038(9); Ir(1)–C(29) = 2.020(8), 2.013(8); N(1)–C(9) = 1.398(13), 1.371(11); N(2)–C(9) = 1.336(12), 1.389(11); N(2)–C(1) = 1.326(11), 1.294(11); C(29)–Ir(1)–N(1) = 171.6(3), 169.5(3); C(1)–Ir(1)–N(3) = 172.8(3), 170.2(3); C(14)–Ir(1)–N(4) = 168.9(3), and 170.3(3).

The isoquinoline derivative **11** was characterized by X-ray diffraction analysis. The structure, which contains two chemically equivalent but crystallographically independent molecules in the asymmetrical unit, demonstrates the formation of the iridaimidazo[1,2-*a*]pyridine bicycle. It formally results from the addition of the NH_2_ group of the amino tautomer of 2-aminopyridine to the triple bonds of the dimeric precursors. As shown for one of the molecules in Figure 7, the donor atoms of the ligands define an octahedron around the iridium atom, displaying *fac* dispositions of carbons and heteroatoms, in a similar manner to its styrylpyridinimine isomer. The most noticeable features of the structure are the bond lengths in the five-member metallaimidazo ring. The distances Ir–C(1) of 1.992(10) and 1.998(9) Å, C(1)–N(2) of 1.326(11) and 1.294(11) Å, and N(2)–C(9) of 1.336(12) and 1.389(11) Å, which are intermediate between single and double bonds, suggest that there is electron delocalization in the bond sequence Ir(1)–C(1)–N(2).^19^ However, the values of the nuclear independent chemical shift (NICS) computed at the center of the five-member ring and out of plane at 1 Å above and below the center (−1.7, −1.2, and −1.4 ppm) are scarcely negative, pointing out very poor aromaticity. The ^1^H and ^13^C{^1^H} NMR spectra of **11** and **12**, at room temperature, in dichloromethane-*d*_2_ are congruous with Figure 7. In the ^1^H spectra, the most remarkable details are the absence of any NH and CHPh resonances and the presence of an AB spin system centered at about 4.0 ppm and defined by Δν ≈ 44 Hz and *J*_A–B_ ≈ 13 Hz, due to the CH_2_Ph substituent of the generated five-member ring. In agreement with a significant double character for the Ir–C bond in the latter, the resonance corresponding to such a carbon atom appears at notable low field, about 228 ppm, in the ^13^C{^1^H} spectra.

**Scheme 4:**
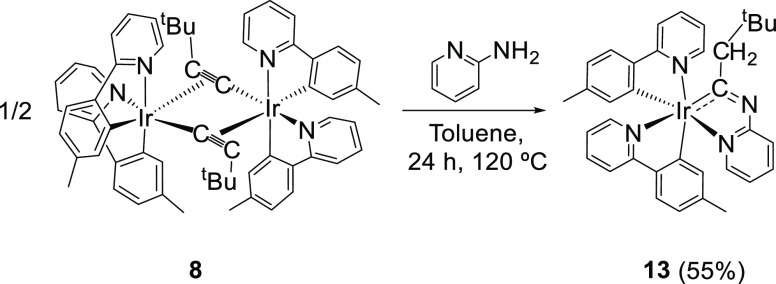
Preparation of Complex **13**

The *tert*-butyl group destabilizes the styrylpyridinimine isomer, while it decreases the activation energy for the formation of the iridaimidazopyridine derivative. Thus, in contrast to **6** and **7**, the treatment of suspensions of the dimer **8**, in toluene, with 1.5 equiv of 2-aminopyridine, at 120 °C, for 24 h directly leads to Ir{κ^2^-*C*,*N*-[C(CH_2_*^t^*Bu)Npy]}{κ^2^-*C*,*N*-(MeC_6_H_3_-py)}_2_ (**13**) with no observation of any styrylpyridinimine isomer (Scheme 4). Complex **13** was isolated as a yellow solid in 55% yield. In accordance with **11** and **12**, its ^1^H NMR spectrum, in dichloromethane-*d*_2_, at room temperature, shows an AB spin system at 2.66 ppm and defined by Δν = 36 Hz and *J*_A–B_ = 14.8 Hz, whereas the ^13^C{^1^H} contains the expected singlet at 234.5 ppm, two characteristic resonances supporting the formation of the iridaimidazo[1,2-*a*]pyridine bicycle also in this case.

### Photophysical and Electrochemical Properties of the Iridaimidazopyridine Derivatives

Table 1 gathers selected absorptions from the UV–vis spectra of 10^–5^ M solutions of **11**–**13**, in 2-methyltetrahydrofuran (2-MeTHF), at room temperature (Figures S1–S3). To fit the bands to their corresponding transitions, we also performed time-dependent DFT (TD-DFT) calculations (B3LYP-D3//SDD(f)/6-31G**) considering tetrahydrofuran. Figures S4–S6 give views of the frontier orbitals. The spectra can be divided into three energy regions: <350, 350–450, and >450 nm. The absorptions observed at energies higher than 350 nm result from ^1^π–π* intra- and interligand transitions. Bands in the range of 350–450 nm correspond to metal-to-ligand combined with ligand-to-ligand or intraligand spin-allowed charge transfers. Weak absorption tails after 450 nm were attributed to formally spin-forbidden transitions, mainly HOMO-to-LUMO, produced by a large spin–orbit coupling resulting from the iridium presence. The HOMO is disposed on the metal center (41–47%) and the 3b (49–51%) and 3b′ (6–8%) ligands, while the LUMO is mainly situated on the 3b ligands (91–96%).

**Table 1:** Selected Calculated (TD-DFT in THF) and Experimental UV–Vis Absorptions for **11**–**13** (in 2-MeTHF) and Their Mayor Contributions

λ exp (nm)	ε (M^–1^ cm^–1^)	exc. energy (nm)	oscilator strength, *f*	transition	character of the transition
Complex **11**					
280	44 100	268	0.0538	HOMO – 6 → LUMO + 2 (74%)	(3b′ → 3b′)
456	4500	471 (*S*_1_)	0.0373	HOMO → LUMO (95%)	(Ir + 3b → 3b)
554	900	554 (*T*_1_)	0	HOMO → LUMO (59%)	(Ir + 3b → 3b)
Complex **12**					
275	186 950	262	0.0394	HOMO – 3 → LUMO + 4 (71%)	(3b → 3b)
355	63 950	343	0.0550	HOMO – 2 → LUMO (84%)	(Ir + 3b′ → 3b)
399	3425	395 (*S*_1_)	0.0245	HOMO → LUMO (85%)	(Ir + 3b → 3b)
466	3300	452 (*T*_1_)	0	HOMO → LUMO (37%)	(Ir + 3b → 3b)
				HOMO → LUMO + 1 (26%)	
Complex **13**					
277	177 680	288	0.1694	HOMO – 5 →LUMO + 1 (62%)	(3b → 3b)
358	58 720	348	0.0896	HOMO – 2 → LUMO (86%)	(Ir + 3b′ → 3b)
401	33 040	397 (*S*_1_)	0.0181	HOMO → LUMO (95%)	(Ir + 3b → 3b)
465	3960	450 (*T*_1_)	0	HOMO → LUMO (36%)	(Ir + 3b → 3b)
				HOMO – 1 → LUMO (22%)	
				HOMO – 1 → LUMO + 1 (12%)	

The electrochemical behavior of **11**–**13** was analyzed to obtain additional information about their frontier orbitals. The cyclic voltammetry measurements were carried out in dichloromethane under argon, using [Bu_4_N]PF_6_ as a supporting electrolyte (0.1 M). Figure S8 shows the voltammograms. Table 2 gathers the potentials versus Fc/Fc^+^. It also includes the HOMO energy levels, obtained from the oxidation potentials, and HOMO and LUMO energy levels were DFT-calculated. Complex **11** exhibits two irreversible oxidations at 0.51 and 1.02 V, whereas two quasi-reversible oxidations are observed for the pyridine counterparts **12** and **13** between 0.30 and 0.95 V. As expected, the HOMO–LUMO gap is significantly smaller for the isoquinoline derivative **11** than for the *p*-tolylpyridine species **12** and **13**.

**Table 2:** Electrochemical and DFT Molecular Orbital Energy Data for **11**–**13**

		obs (eV)	calcd (eV)		
complex	*E*^ox^ (V)	HOMO^a^	HOMO	LUMO	HLG^b^
**11**	0.51, 1.02	–5.31	–5.17	–1.85	3.32
**12**	0.38,^c^ 0.87^c^	–5.18	–5.13	–1.24	3.89
**13**	0.35,^c^ 0.93^c^	–5.15	–5.15	–1.27	3.88

a HOMO = −[*E*^ox^ versus Fc/Fc^+^ + 4.8] eV.

b HGL = LUMO – HOMO.

c *E*_1/2_^ox^.

Complexes **11**–**13** are the first members of the iridaimidazopyridine family of phosphorescent iridium(III) emitters. They are emissive upon photoexcitation in a doped poly(methyl methacrylate) (PMMA) film at 5 wt %, at room temperature, and 2-MeTHF at room temperature and at 77 K. Table 3 collects the main photophysical features. The estimated values, from the difference in energy between the optimized triplet states *T*_1_ and the singlet states *S*_0_ in tetrahydrofuran, are almost equal to those experimentally obtained, as expected for emissions corresponding to *T*_1_ excited states.

**Table 3:** Photophysical Data of Complexes **11**–**13**

calcd λ_em_ (nm)	media (*T*/K)	λ_em_ (nm)	τ (μs)	Φ	*k*_r_^a^ (s^–1^)	*k*_nr_^a^ (s^–1^)	*k*_r_/*k*_nr_
Complex **11**							
635	PMMA (298)	632	1.8	0.12	6.7 × 10^4^	4.9 × 10^5^	0.1
	2-MeTHF (298)	594, 625	3.6	0.14	3.9 × 10^4^	2.4 × 10^5^	0.2
	2-MeTHF (77)	572, 619	7.4				
Complex **12**							
507	PMMA (298)	489, 514	1.3	0.75	5.8 × 10^5^	1.9 × 10^5^	3.1
	2-MeTHF (298)	490, 514	2.3	0.76	3.3 × 10^5^	1.0 × 10^5^	3.3
	2-MeTHF (77)	473, 507	4.2				
Complex **13**							
480	PMMA (298)	497, 513	1.9	∼1	5.3 × 10^5^		
	2-MeTHF (298)	493, 517	2.2	∼1	4.5 × 10^5^		
	2-MeTHF (77)	473, 509	3.5				

a Calculated according to *k*_r_ = Φ/τ and *k*_nr_ = (1 – Φ)/τ.

**Figure 8:**
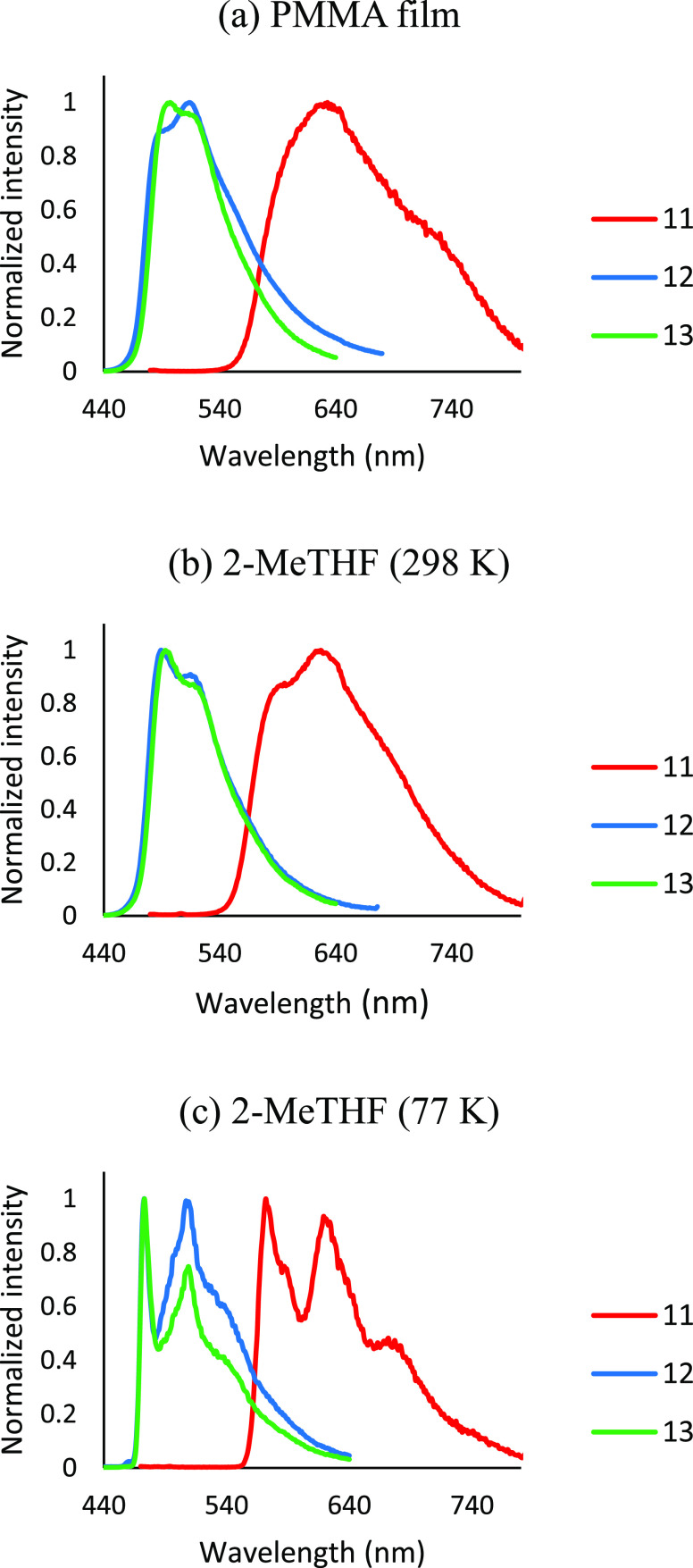
(a) Emission spectra of **11**, **12**, and **13** in 5 wt % PMMA films at 298 K. (b) Emission spectra of **11**, **12**, and **13** in 2-MeTHF at 298 K. (c) Emission spectra of **11**, **12**, and **13** in 2-MeTHF at 77 K.

Isoquinoline complex **11** is an orange emitter (572–632 nm), which displays lifetimes in the range of 7.4–1.8 μs and moderate quantum yields of about 0.13. In contrast, the *p*-tolylpyridine counterparts **12** and **13** are very efficient green emitters (473–517 nm) as expected from a higher HOMO–LUMO gap. They exhibit shorter lifetimes, 4.2–1.3 μs, and quantum yields higher than 0.75. Worthy of note is the quantum yield of **13**, which reaches the unity in both the PMMA film and 2-MeTHF at room temperature. Another noticeable feature of **12** and **13** with regard to **11** is their narrower emissions. This, which is evident in the emission spectra (Figure 8), points out a lower difference between the structure of the excited state and the ground state for the *p*-tolylpyridine case.^3a^ The spectra of the three compounds also show broad structureless bands at room temperature, which split into vibronic fine structures in 2-MeTHF at 77 K in a congruent manner with a significant participation of ligand-centered ^3^π–π* transitions in the excited state.^20^

### Electroluminescence (EL) Properties of an Organic Light-Emitting Diode (OLED) Device

**Figure 9:**
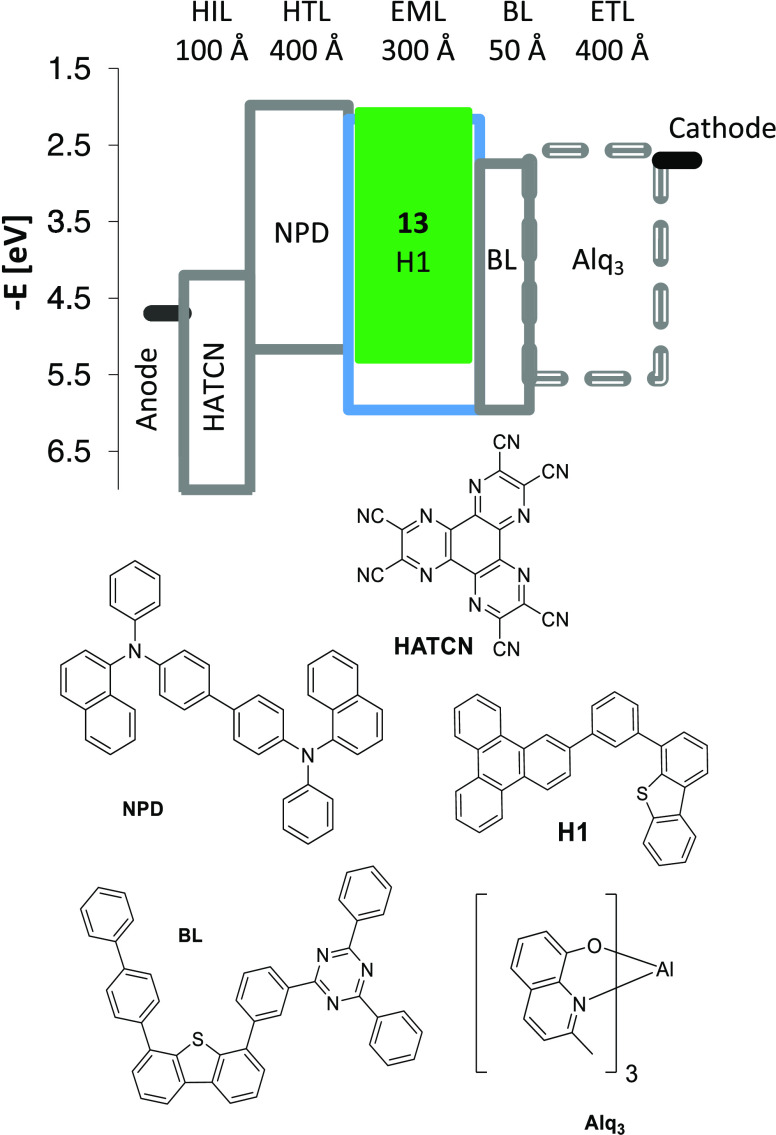
Device structure, energy levels (eV), and molecular structures of the materials used.

To support the applicability of the developed synthetic methodology in the fabrication of OLED devices, complex **13** as an example of saturated green phosphorescent emitters has been tested in bottom-emission OLED structures. Figure 9 shows a scheme of the devices, including energy levels, layer thickness, and materials.

**Figure 10:**
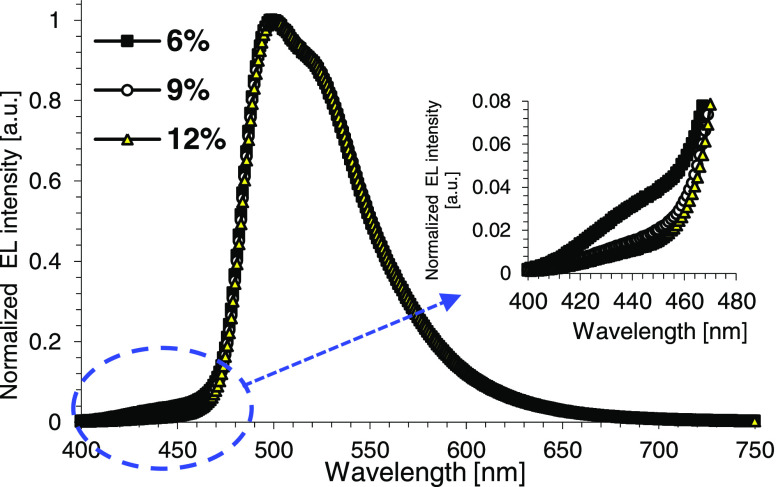
Electroluminescence (EL) spectra of the devices measured at 10 mA/cm^2^.

**Figure 11:**
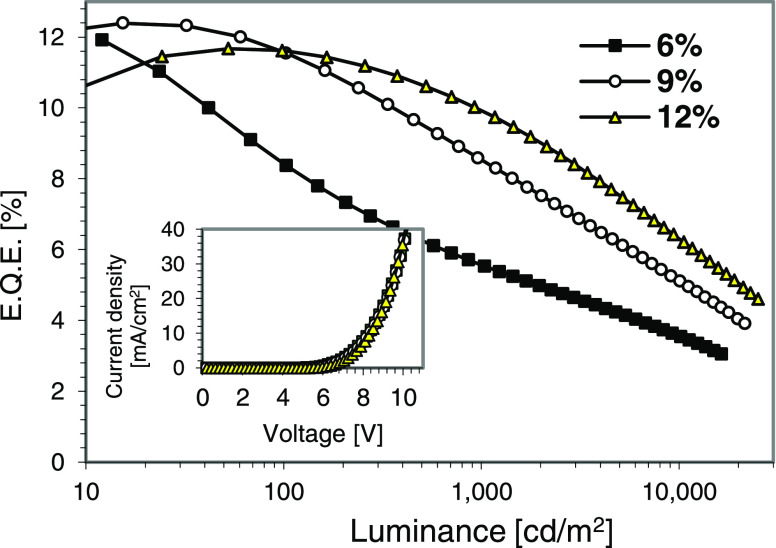
EQE versus luminance correlation plot. Inset: device voltage–current density plot.

The devices were made by high-vacuum (<10^–7^ Torr) thermal evaporation. The anode electrode was 750 Å of indium tin oxide (ITO). The cathode was composed by 10 Å of LiF and 1000 Å of Al. All devices were encapsulated with an epoxy-sealed glass lid glovebox (<1 ppm H_2_O and O_2_) immediately after building, and a moisture scavenger was incorporated within the package. Following the anode-to-cathode sequence, the device organic stack consisted of 100 Å of HATCN as the hole injection layer (HIL); 400 Å of *N*,*N*′-di(1-naphthyl)-*N*,*N*′-diphenyl-(1,1′-biphenyl) 4,4′-diamine (NPD) as a hole-transporting layer (HTL); 300 Å of an emissive layer (EML) containing the host (H1) doped with complex **13** as a green emitter at the investigated concentration; 50 Å of hole blocker material (BL); and 400 Å of Alq_3_ as an electron-transporting layer (ETL). Concentrations of 6, 9, and 12% of emitter were compared side by side in the same structure. The device performance is summarized in Table 4. Electroluminescence (EL) spectra are shown in Figure 10, whereas Figure 11 displays external quantum efficiency (EQE) versus luminance and plots of current density versus voltage (see the inset).

**Table 4:** Performance of Devices Based on Complex **13**

	1931 CIE				at 1000 cd/m^2^			
emitter (%)	*x*	*y*	λ max (nm)	FWHM (nm)	voltage (V)	LE (cd/A)	EQE (%)	PE (lm/W)
6	0.234	0.569	499	68	7.7	16.9	5.6	6.9
9	0.237	0.582	500	68	7.4	26.3	8.5	11.2
12	0.239	0.585	500	69	7.3	30.7	9.9	13.2

Electroluminescence spectra of the fabricated devices revealed that complex **13** provided very saturated green emission with maximum wavelength at 500 nm, full width at half maximum (FWHM) of 68 nm, and emission offset about 470 nm (Figure 10). It corresponds to over 2.6 eV triplet emission energy of the emitter. On the other hand, maximum EQE slightly over 12% was observed, which is low for phosphorescent devices of this class displaying high efficiency. The reason appears to be related to the low triplet of the NPD hole-transporting layer since higher triplet material layers are required to efficiently confine the high triplet excitons of the emitter. In this context, the presence of a clear emission shoulder around 430–440 nm in the EL spectrum of the device containing 6% of emitter **13** should be pointed out (see the expansion in Figure 10). It originates from the NPD layer and strongly supports exciton leakage from the emissive layer and quenching by the low triplet of NPD.

One way to improve the device performance is to increase the emitter concentration. This increase should move the recombination zone away from the low triplet NPD HTL interface, minimizing interface quenching and thus improving the device efficiency. This is exactly what is observed from the device performance. Increasing the emitter concentration from 6 to 9 to 12% significantly improves device EQE, especially at higher luminance levels (see Table 4 and Figure 11), and reduces the amount of the undesirable NPD emission shoulder in the device EL spectrum (see the expansion in Figure 8). A further increase in the emitter concentration over 12%, however, causes concentration emission quenching, which results in the reduction of device efficiency.

## Concluding Remarks

Acetylide anions have received considerable attention as ancillary ligands in connection with the design of transition metal phosphorescent emitters;^21^ their strong field character creates a strong interaction through a p_π_–d_π_ overlap, which contributes to raise the metal-centered d–d energy states. This study reveals that they are much more. In addition to improve the photophysical properties of the emitters, they have now demonstrated an extraordinary synthetic usefulness. Acetylide anions stabilize structures that are elusive for other 3e-donor ligands. Thus, the use of such ability allows us to design alternative synthetic precursors to those currently employed for the preparation of phosphorescent emitters. As a consequence, emitters with unusual stereochemistries can be easily prepared with their properties studied. Furthermore, the coordination of the acetylide to a metal center modifies and enhances the reactivity of the carbon atoms of the triple bond, converting it into an interesting building block, which on the metal coordination sphere generates new types of ligands characteristic of novel families of emitters.

Dimers **6**–**8**, with a cis disposition of the heterocycles, and their transformation first into styrylpyridinimine derivatives and later into the iridaimidazo[1,2-*a*]pyridine emitters **11**–**13**, of an octahedral structure with a *fac* disposition of carbon and nitrogen atoms, are clear concept validation proofs of what we say. The quantum yields of 100% displayed by the green emitter **13**, in both the PMMA film and 2-MeTHF at room temperature, should be furthermore highlighted from the point of view of the photophysical properties.

The designed synthetic pathway goes beyond a conceptual improvement; it has practical applicability as demonstrated by the fabrication of OLED devices based on complex **13**. In this context, it should be mentioned that such an emitter demonstrated in the device very saturated green emission at a peak wavelength of 500 nm, with an external quantum efficiency of over 12% or 30.7 cd/A luminous efficacy. Such deep-green color saturation phosphorescent emitters can find application in future OLED displays with BT.2020 specification.

Having the door opened and the procedure shown, novel families of emitters are expected in a near future; some of them are certainly already on the way.

## Experimental Section

### General Information

All reactions were carried out under argon with dried solvents and using Schlenk tube techniques. Instrumental methods are given in the Supporting Information. In the NMR spectra, chemical shifts (expressed in ppm) are referenced to residual solvent peaks, and coupling constants (*J*) are given in hertz. Signals were assigned using also bidimensional NMR spectra (^1^H–^1^H correlated spectroscopy (COSY), ^1^H–^13^C{^1^H} heteronuclear single quantum coherence (HSQC), and ^1^H–^13^C{^1^H} heteronuclear multiple bond correlation (HMBC)). *trans-*[Ir(μ-OH){κ^2^-*C*,*N*-(C_6_H_4_-Isoqui)}_2_]_2_ (**1**) and *trans-*[Ir(μ-OH){κ^2^-*C*,*N*-(MeC_5_H_3_-py)}_2_]_2_ (**2**) were prepared according to the published methods.^8e^

#### Preparation of *trans-*[Ir(μ_2_-η^2^-C≡CPh){κ^2^-*C*,*N*-(C_6_H_4_-Isoqui)}_2_]_2_ (**3**)

In a Schlenk flask, a suspension of **1** (2000 mg, 1.619 mmol) in toluene (60 mL) was treated with phenylacetylene (890 μL, 8.094 mmol), and the mixture was stirred at room temperature, for 48 h. The resulting brown suspension was dried under vacuum, and the crude was purified by column chromatography (basic Al_2_O_3_, activity grade V) using dichloromethane as an eluent to give as a red solid. Yield: 1570 mg (69%). X-ray quality crystals were grown by slow evaporation of a concentrate solution of the solid in dichloromethane at room temperature. Anal. Calcd for C_76_H_50_Ir_2_N_4_: C, 65.03; H, 3.59; N, 3.99. Found: C, 65.32; H, 3.73; N, 3.87. High-resolution mass spectrometry (HRMS) (electrospray, *m*/*z*) calcd for C_76_H_51_Ir_2_N_4_ [M + H]: 1405.3367; found: 1405.3424. Calcd for C_38_H_26_IrN_2_ [M/2 + H]: 703.1720; found: 703.1660. IR (cm^–1^): ν(C≡C) 1991. ^1^H NMR (300 MHz, CD_2_Cl_2_, 298 K): δ 9.28 (d, ^3^*J*_H–H_ = 6.5, 4H, CH isoqui), 8.78 (d, ^3^*J*_H–H_ = 8.3, 4H, CH Ph-isoqui), 8.00 (d, ^3^*J*_H–H_ = 8.1, 4 H, CH Ph-acetylene), 7.73 (m, 12H, CH Ph-isoqui), 6.74 (m, 6H, CH Ph-acetylene), 6.51 (m, 12H, CH isoqui), 6.14 (d, ^3^*J*_H–H_ = 8.2, 4H, CH isoqui), 6.02 (d, ^3^*J*_H–H_ = 7.6, 4H, CH isoqui). ^13^C{^1^H} NMR (75 MHz, CD_2_Cl_2_, 298 K): δ 169.4 (s, C–N isoqui), 164.0 (s, C Ph-isoqui), 145.4 (s, C Ph-isoqui), 144.2 (s, N–CH isoqui), 137.0 (s, C isoqui), 131.0 (s, CH isoqui), 130.9 (s, CH Ph-isoqui), 130.6 (s, CH isoqui), 130.0 (s, CH *o*-Ph-acetylene), 129.6 (s, CH isoqui), 127.9 (s, CH Ph-isoqui), 127.7 (s, CH Ph-isoqui), 127.7 (s, CH Ph-isoqui), 127.4 (s, CH Ph-isoqui), 127.3 (s, CH isoqui), 127.2 (s, C Ph-isoqui), 125.0 (s, CH *p*-Ph-isoqui), 120.7 (s, CH *m*-Ph-isoqui), 119.6 (s, CH isoqui), 103.8 (s, Ir–C≡*C*–Ph), 79.3 (s, Ir–*C*≡C–Ph).

#### Preparation of *trans-*[Ir(μ_2_-η^2^-C≡CPh){κ^2^-*C*,*N*-(MeC_6_H_3_-py)}_2_]_2_ (**4**)

In a Schlenk flask, a suspension of **2** (2000 mg, 1.833 mmol) in toluene (80 mL) was treated with phenylacetylene (1 mL, 9.163 mmol), and the mixture was stirred at room temperature, for 48 h. The resulting yellow suspension was allowed to settle, and the liquid phase was removed. The formed yellow solid was washed with pentane (3 × 5 mL) and dried under vacuum. Yield: 2220 mg (96%). X-ray quality crystals were grown by layering a solution of this complex in toluene with MeOH at 4 °C. Anal. Calcd for C_64_H_50_Ir_2_N_4_: C, 61.03; H, 4.00; N, 4.45. Found: C, 61.31; H, 4.36; N, 4.15. HRMS (electrospray, *m*/*z*) calcd for C_64_H_50_Ir_2_N_4_Na [M + Na]: 1283.3186; found: 1283.3072. Calcd for C_32_H_25_IrN_2_ [M/2 + Na]: 653.1539; found: 653.1477. IR (cm^–1^) ν(C≡C): 1911. ^1^H NMR (300 MHz, CD_2_Cl_2_, 298 K): δ 9.34 (dd, ^3^*J*_H–H_ = 5.9, ^4^*J*_H–H_ = 0.8, 4H, CH py), 7.73 (d, ^3^*J*_H–H_ = 8.2, 4H, CH py), 7.63 (ddd, ^3^*J*_H–H_ = 8.2, ^3^*J*_H–H_ = 7.7, ^4^*J*_H–H_ = 1.6, 4H, CH py), 7.29 (d, ^3^*J*_H–H_ = 7.9, 4H, CH MeC_6_H_3_-py), 6.72 (m, 6H, CH py, CH *p*-Ph-acetylene), 6.60 (t, ^3^*J*_H–H_ = 7.9, 4H, CH *m*-Ph-acetylene), 6.52 (d, ^3^*J*_H–H_ = 7.9, 4H, CH MeC_6_H_3_-py), 6.13 (dd, ^2^*J*_H–H_ = 7.9, ^4^*J*_H–H_ = 1.2, 4H, CH *o*-Ph-acetylene), 5.68 (s, 4H, CH MeC_6_H_3_-py), 1.91, (s, 12H, CH_3_ MeC_6_H_3_-py). ^13^C{^1^H} NMR (75 MHz, CD_2_Cl_2_, 298 K): δ 169.3 (s, C–N py), 161.0 (s, C–Ir MeC_6_H_3_-py), 151.5 (s, N–CH py), 141.5 (s, C MeC_6_H_3_-py), 139.8 (s, C MeC_6_H_3_-py), 136.2 (s, CH py), 131.4 (s, CH MeC_6_H_3_-py), 130.6 (s, CH, *o*-Ph-acetylene), 127.8 (s, C Ph-acetylene), 127.1 (s, CH *m*-Ph-acetylene), 124.8 (s, CH *p*-Ph-acetylene), 124.0 (s, CH MeC_6_H_3_-py), 122.1 (s, CH MeC_6_H_3_-py), 121.5 (s, CH pyridine), 119.1 (s, CH pyridine), 102.5 (s, Ir–C≡*C*–Ph), 79.0 (s, Ir–*C*≡C–Ph), 22.0 (s CH_3_ MeC_6_H_3_-py).

#### Preparation of *trans-*[Ir(μ_2_-η^2^-C≡C*^t^*Bu){κ^2^-*C*,*N*-(MeC_6_H_3_-py)}_2_]_2_ (**5**)

In a Schlenk flask, a suspension of **2** (2000 mg, 1.833 mmol) in toluene (80 mL) was treated with *tert*-butylacetylene (1 mL, 8.120 mmol), and the mixture was stirred at room temperature, for 48 h. The resulting yellow suspension was allowed to settle, and the liquid phase was removed. The formed yellow solid was washed with pentane (3 × 10 mL) and dried under vacuum. Yield: 1.63 g (73%). Anal. Calcd for C_64_H_50_Ir_2_N_4_: C, 59.09; H, 4.79; N, 4.59. Found: C, 59.36; H, 4.45; N, 4.21. HRMS (electrospray, *m*/*z*) calcd for C_60_H_58_Ir_2_N_4_ [M]: 1220.3915; found: 1220.3921. Calcd for C_30_H_28_IrN_2_ [M/2 – H]: 609.1876 found: 609.1876. IR (cm^–1^): ν(C≡C) 1942. ^1^H NMR (300 MHz, CD_2_Cl_2_, 298 K): δ 9.14 (dd, ^3^*J*_H–H_ = 5.9, ^4^*J*_H–H_ = 0.9, 4H, CH py), 7.78 (d, ^3^*J*_H–H_ = 8.2, 4H, CH py), 7.57 (ddd, ^3^*J*_H–H_ = 8.2; 7.3, ^4^*J*_H–H_ = 1.6, 4H, CH py), 7.51 (d, ^3^*J*_H–H_ = 7.9, 4H, CH MeC_6_H_3_-py), 6.64 (dd, ^3^*J*_H–H_ = 7.9, ^4^*J*_H–H_ = 1.1, 4H, CH MeC_6_H_3_-py), 6.58 (ddd, ^3^*J*_H–H_ = 7.3; 5.9, ^4^*J*_H–H_ = 1.5, 4H, CH py), 5.79 (s, 4H, CH MeC_6_H_3_-py), 1.95 (s, 12H, CH_3_ MeC_6_H_3_-py), 0.40 (s, 18H, *^t^*Bu). ^13^C{^1^H} NMR (75 MHz, CD_2_Cl_2_, 298 K): δ 169.6 (s, N–C py), 161.5 (s, Ir–C MeC_6_H_3_-py), 151.6 (s, N–CH py), 142.1 (s, C MeC_6_H_3_-py), 139.0 (s, C MeC_6_H_3_-py), 135.8 (s, CH py), 132.9 (s, CH MeC_6_H_3_-py), 123.7 (s, CH MeC_6_H_3_-py), 121.8 (s, CH MeC_6_H_3_-py), 121.0 (s, CH py), 118.5 (s, CH py), 114.6 (s, Ir–C≡*C*–*^t^*Bu), 71.1 (s, Ir–*C*≡C–*^t^*Bu), 32.3 (s, CH_3_*^t^*Bu), 32.3 (s, C *^t^*Bu, inferred from the HMBC spectrum), 21.9 (s, CH_3_ MeC_6_H_3_-py).

#### Isomerization of *trans-*[Ir(μ_2_-η^2^-C≡CPh){κ^2^-*C*,*N*-(C_6_H_4_-Isoqui)}_2_]_2_ (**3**) to *cis-*[Ir(μ_2_-η^2^-C≡CPh){κ^2^-*C*,*N*-(C_6_H_4_-Isoqui)}_2_]_2_ (**6**)

A suspension of **3** (500 mg, 0.356 mmol) in toluene (30 mL) was stirred in a Schlenk flask, equipped with a poly(tetrafluoroethylene) (PTFE) stopcock, at 120 °C. After 72 h, the volume was reduced until approximately 1 mL, and the liquid was removed. The obtained orange-red solid was washed with dichloromethane (3 × 1 mL) and dried under vacuum. Yield: 380 mg (76%). X-ray quality crystals were grown by slow evaporation of a concentrate solution of the solid in dichloromethane at room temperature. Anal. Calcd for C_76_H_50_Ir_2_N_4_: C, 65.03; H, 3.59; N, 3.99. Found: C, 65.41; H, 3.86; N, 3.76. HRMS (electrospray, *m*/*z*) calcd for C_76_H_50_Ir_2_N_4_Na [M + Na]: 1427.3186; found: 1427.3172. Calcd for C_38_H_26_IrN_2_ [M/2 + H]: 703.1720; found: 703.1805. IR (cm^–1^): ν(C≡C) 1982, 2015. ^1^H NMR (300 MHz, CD_2_Cl_2_, 298 K): δ 9.08 (m, 2H, CH arom), 8.89 (d, ^3^*J*_H–H_ = 6.53, 4H, CH arom), 8.57 (d, ^3^*J*_H–H_ = 8.32, 2H, CH arom), 8.24 (d, ^3^*J*_H–H_ = 7.98, 2H, CH arom), 8.07 (m, 2H, CH arom), 7.84 (m, 4H, CH arom), 7.43 (m, 8H, CH arom), 7.22 (m, 4H, CH arom), 6.87, (m, 2H, CH arom), 6.61 (m, 4H, CH arom), 6.50 (d, ^3^*J*_H–H_ = 6.14, 2H, CH arom), 6.33 (m, 10H, CH arom), 6.18 (m, 2H, CH arom), 6.10, (d, ^3^*J*_H–H_ = 5.91, 2H, CH arom). The low solubility of the solid precluded to obtain its ^13^C{^1^H} NMR spectrum.

#### Isomerization of *trans-*[Ir(μ_2_-η^2^-C≡CPh){κ^2^-*C*,*N*-(MeC_6_H_3_-py)}_2_]_2_ (**4**) to *cis-*[Ir(μ_2_-η^2^-C≡CPh){κ^2^-*C*,*N*-(MeC_6_H_3_-py)}_2_]_2_ (**7**)

A suspension of **4** (1000 mg, 0.794 mmol) in toluene (80 mL) was stirred in a Schlenk flask, equipped with a PTFE stopcock, at 120 °C. After 72 h, the volume was reduced until approximately 1 mL, and the liquid was removed. The obtained yellow solid was washed with toluene (2 × 1 mL) and pentane (3 × 3 mL) and dried under vacuum. Yield: 531 mg (53%). X-ray quality crystals were grown by slow evaporation of a concentrate solution of the solid in dichloromethane at room temperature. Anal. Calcd for C_64_H_50_Ir_2_N_4_: C, 61.03; H, 4.00; N, 4.45. Found: C, 60.91; H, 3.65; N, 4.20. HRMS (electrospray, *m*/*z*): Calcd for C_64_H_50_Ir_2_N_4_Na [M + Na]: 1283.3186; found: 1283.3196. IR (cm^–1^): ν (C≡C) 2022, 1928. ^1^H NMR (300 MHz, CD_2_Cl_2_, 298 K): δ 9.01 (dd, ^3^*J*_H–H_ = 5.6, ^4^*J*_H–H_ = 0.94, 2H, CH py), 8.41 (s, 2H, CH MeC_6_H_3_-py), 7.95 (d, ^3^*J*_H–H_ = 8.1, 2H, CH py), 7.65 (m, 2H, CH py), 7.59 (d, ^3^*J*_H–H_ = 8.0, 2H, CH MeC_6_H_3_-py), 7.39 (d, ^3^*J*_H–H_ = 8.0, 2H, CH MeC_6_H_3_-py), 7.18 (d, ^3^*J*_H–H_ = 8.1, 2H, CH py), 7.00 (m, 4H, CH py), 6.95 (dd, ^3^*J*_H–H_ = 7.9, ^4^*J*_H–H_ = 1.3, 2H, CH MeC_6_H_3_-py), 6.65 (m, 10H, CH Ph-acetylene, CH py, CH MeC_6_H_3_-py), 6.44 (s, 2H, CH MeC_6_H_3_-py), 6.31 (m, 6H, CH Ph-acetylene, CH py), 6.22 (m, 2H, CH py), 2.32 (s, 6H, CH_3_ MeC_6_H_3_-py), 1.91 (s, 6H, CH_3_ MeC_6_H_3_-py). ^13^C{^1^H} NMR (75 MHz, CD_2_Cl_2_, 298 K): δ 167.1 (s, N–C py), 166.4 (s, N–C py), 156.7 (s, C MeC_6_H_3_-py), 151.5 (s, C MeC_6_H_3_-py), 147.8 (s, N–CH py), 145.8 (s, N–CH py), 141.6 (s, Ir–C MeC_6_H_3_-py), 141.5 (s, Ir–C MeC_6_H_3_-py), 140.7 (s, CH MeC_6_H_3_-py), 140.0 (s, C MeC_6_H_3_-py), 139.2 (s, C MeC_6_H_3_-py), 137.6 (s, CH MeC_6_H_3_-py), 137.2 (s, CH py), 135.4 (s, CH py), 130.6 (s, CH Ph-acetylene), 127.4 (s, C Ph-acetylene), 127.3 (s, CH Ph-acetylene), 124.7 (s, CH Ph-acetylene), 123.9 (s, CH MeC_6_H_3_-py), 123.9 (s, CH MeC_6_H_3_-py), 123.2 (s, CH MeC_6_H_3_-py), 122.3 (s, CH py), 122.0 (s, CH MeC_6_H_3_-py), 120.6 (s, CH py), 119.3 (s, CH py), 118.3 (s, CH py), 92.5 (s, Ir–C≡*C*–Ph), 72.8 (s, Ir–*C*≡C–Ph), 22.1 (s, CH_3_ MeC_6_H_3_-py), 21.8 (s, CH_3_ MeC_6_H_3_-py).

#### Isomerization of *trans-*[Ir(μ_2_-η^2^-C≡C*^t^*Bu){κ^2^-*C*,*N*-(MeC_6_H_3_-py)}_2_]_2_ (**5**) to *cis-*[Ir(μ_2_-η^2^-C≡C*^t^*Bu){κ^2^-*C*,*N*-(MeC_6_H_3_-py)}_2_]_2_ (**8**)

A suspension of **5** (1000 mg, 0.820 mmol) in toluene (80 mL) was stirred in a Schlenk flask, equipped with a PTFE stopcock, at 120 °C. After 72 h, the volume was reduced until approximately 3 mL, and the liquid was removed. The obtained yellow solid was washed with toluene (2 × 2 mL) and pentane (3 × 3 mL) and dried under vacuum. Yield: 873 mg (87%). Anal. Calcd for C_60_H_58_Ir_2_N_4_: C, 59.09; H, 4.79; N, 4.59. Found: C, 58.91; H, 4.63; N, 4.76. HRMS (electrospray, *m*/*z*) calcd for C_60_H_58_Ir_2_N_4_ [M]: 1220.3915; found: 1220.3928. IR (cm^–1^): ν (C≡C) 1942. ^1^H NMR (300 MHz, CD_2_Cl_2_, 298 K): δ 8.64 (ddd, ^3^*J*_H–H_ = 5.54, ^4^*J*_H–H_ = 1.66, ^5^*J*_H–H_ = 0.75, 2H, CH py), 8.49 (s, 2H, CH MeC_6_H_3_-py), 7.86 (d, ^3^*J*_H–H_ = 8.15, 2H, CH py), 7.73 (d, ^3^*J*_H–H_ = 8.18, 2H, CH py), 7.57 (m, 6H. 2H CH py, 4H CH MeC_6_H_3_-py), 7.35 (ddd, ^3^*J*_H–H_ = 8.18; 7.28, ^4^*J*_H–H_ = 1.58, 2H, CH py), 6.91 (dd, ^3^*J*_H–H_ = 7.87, ^4^*J*_H–H_ = 1.20, 2H, CH MeC_6_H_3_-py), 6.84 (ddd, ^3^*J*_H–H_ = 5.77, ^4^*J*_H–H_ = 1.55, ^5^*J*_H–H_ = 0.74, 2H, CH py), 6.67 (dd, ^3^*J*_H–H_ = 7.87, ^4^*J*_H–H_ = 1.08, 2H, CH MeC_6_H_3_-py), 6.50 (m, 4H, CH py), 6.31 (s, 2H, CH MeC_6_H_3_-py), 2.30 (s, 6H, CH_3_ MeC_6_H_3_-py), 1.95 (s, 6H, CH_3_ MeC_6_H_3_-py), 0.42 (s, 18H, *^t^*Bu). ^13^C{^1^H} NMR (75 MHz, CD_2_Cl_2_, 298 K): δ 168.2 (s, N–C py), 166.0 (s, N–C py), 157.4 (s, C MeC_6_H_3_-py), 155.8 (s, C MeC_6_H_3_-py), 147.4 (s, N–CH py), 147.2 (s, N–CH py), 141.0 (s, Ir–C MeC_6_H_3_-py), 140.6 (s, Ir–C MeC_6_H_3_-py), 139.9 (s, CH MeC_6_H_3_-py), 138.8 (s, C MeC_6_H_3_-py), 138.4 (s, C MeC_6_H_3_-py), 136.9 (s, CH MeC_6_H_3_-py), 136.1 (s, CH py), 135.4 (s, CH py), 123.1 (s, CH MeC_6_H_3_-py), 122.9 (s, CH MeC_6_H_3_-py), 122.0 (s, CH MeC_6_H_3_-py), 121.3 (s, CH py), 120.5 (s, CH MeC_6_H_3_-py), 119.4 (s, CH py), 118.2 (s, CH py), 117.6 (s, CH py), 102.4 (s, Ir–C≡*C*–*^t^*Bu), 71.9 (s, Ir–*C*≡C–*^t^*Bu), 32.2 (s, CH_3_*^t^*Bu), 32.0 (s, C *^t^*Bu), 21.1 (s, CH_3_ MeC_6_H_3_-py), 21.0 (s, CH_3_ MeC_6_H_3_-py).

#### Preparation of Ir{κ^2^-*C*,*N*-[C(=CHPh)-py-NH]}{κ^2^-*C*,*N*-(C_6_H_4_-Isoqui)}_2_ (**9**)

A suspension of **6** (300 mg, 0.214 mmol) in toluene (15 mL), placed in a Schlenk flask equipped with a PTFE stopcock, was treated with 2-aminopyridine (60 mg, 0.641 mmol). The mixture was held during 24 h at 120 °C. Afterward, the solution was concentrated until approximately 1 mL, and pentane was added. The resulting red solid was washed with pentane (3 × 3 mL) and dried under vacuum. Yield: 231 mg (68%). X-ray quality crystals were grown by layering a solution of this complex in toluene with MeOH at 4 °C. Anal. Calcd for C_43_H_31_IrN_4_: C, 64.89; H, 3.93; N, 7.04. Found: C, 64.76; H, 3.89, N, 6.87. HRMS (electrospray, *m*/*z*): Calcd for C_43_H_32_IrN_4_ [M + H]: 797.2251; found: 797.2262. IR (cm^–1^): ν(N=H) 3352, 3333. ^1^H and ^13^C{^1^H} spectra show the formation of two isomers in a 60:40 ratio. ^1^H NMR (500 MHz, CD_2_Cl_2_, 298 K): δ 8.97 (m, 1.6H, CH arom both isomers), 8.55 (d, ^3^*J*_H–H_ = 6.1, 0.6H, CH arom *E* isomer), 8.36 (d, ^3^*J*_H–H_ = 6.1, 0.4H, CH arom *Z* isomer), 8.23 (m, 2.0H, CH arom both isomers), 7.97 (dd, ^3^*J*_H–H_ = 7.4, ^4^*J*_H–H_ = 1.2, 0.4H, CH arom *Z* isomer), 7.91 (m, 0.6H, CH arom *E* isomer), 7.88 (m, 0.6H, CH arom *E* isomer), 7.83 (m, 0.8H, CH arom *Z* isomer), 7.65 (m, 5.4H, CH arom both isomers), 7.52 (d, ^3^*J*_H–H_ = 6.1, 0.6H, CH arom *E* isomer), 7.42 (m, 0.8H, CH arom *Z* isomer), 7.32 (m, 1.2H, CH arom both isomers), 7.13–6.82 (m, 7.0H, CH arom both isomers), 6.73 (m, 1.8H, CH arom both isomers), 6.56 (m, 0.8H, CH arom *Z* isomer), 6.43 (m, 3.8H, CH arom both isomers + =C*H*Ph *Z* isomer, inferred from the HMBC spectrum), 5.98 (ddd, ^3^*J*_H–H_ = 7.3; 6.3, ^4^*J*_H–H_ = 1.3, 0.4H, CH py *Z* isomer), 5.78 (s, 0.5H, NH *E* isomer), 5.58 (ddd, ^3^*J*_H–H_ = 7.2; 6.4, ^4^*J*_H–H_ = 1.3, 0.6H, CH py *E* isomer), 5.49 (s, 0.3H, NH *Z* isomer), 4.92 (s, 0.6H, =C*H*Ph *E* isomer). ^13^C{^1^H} NMR (100 MHz, CD_2_Cl_2_, 298 K): δ 168.8, 168.6, 168.6, 168.4, 167.9, 166.6, 165.0, 164.8 (s, C arom), 163.2, 162.0 (s, C py), 158.8 (s, C arom), 149.7 (s, Ir–C–N *E* isomer), 149.1 (s, Ir–C–N *Z* isomer), 146.6, 146.4, 146.1 (s, C arom), 141.6, 141.4 (s, CH arom), 141.0 (s, C arom), 140.8 (s, CH arom), 139.1 (s, CH arom), 138.1, 137.8, 137.6, 137.6 (s, CH arom), 137.5, 137.2, 137.2, 137.1 (s, C arom), 136.6, 136.4, 136.1, 133.6, 130.9, 130.7, 130.6, 130.5, 130.4, 130.0, 129.6, 129.5, 129.5, 129.0, 128.8, 128.4, 128.1, 128.0, 127.9, 127.8, 127.8, 127.5, 127.4, 127.0, 127.0 (s, CH arom), 126.8, 126.7, 126.4 (s, C arom), 126.2, 124.6, 124.0, 123.4, 120.7, 119.9, 119.8, 119.8, 119.7, 119.6, 119.4, 118.9, 118.1, 117.0 (s, CH arom), 106.1, 104.0 (s, CH py).

#### Preparation of Ir{κ^2^-*C*,*N*-[C(=CHPh)-py-NH]}{κ^2^-*C*,*N*-(MeC_6_H_3_-py)}_2_ (**10**)

A suspension of **7** (300 mg, 0.214 mmol) in toluene (15 mL), placed in a Schlenk flask equipped with a PTFE stopcock, was treated with 2-aminopyridine (70 mg, 0.744 mmol). The mixture was held during 24 h, at 120 °C. Afterward, the solution was concentrated until approximately 1 mL, and pentane was added to afford an orange solid, which was washed with pentane (3 × 3 mL) and dried under vacuum. Yield: 262 mg (76%). X-ray quality crystals were grown by layering a solution of this complex in toluene with MeOH at 4 °C. Anal. Calcd for C_37_H_31_IrN_4_: C, 61.39; H, 4.32; N, 7.74. Found: C, 61.00; H, 4.17; N, 7.56. HRMS (electrospray, *m*/*z*) calcd for C_37_H_32_IrN_4_ [M + H]: 797.2251; found: 797.2262. IR (cm^–1^): ν(N=H) 3374, 3352. ^1^H and ^13^C{^1^H} spectra show the formation of two isomers in a 60:40 ratio. ^1^H NMR (300 MHz, CD_2_Cl_2_, 298 K): δ 8.56 (ddd, ^3^*J*_H–H_ = 5.5, ^4^*J*_H–H_ = 1.7, ^5^*J*_H–H_ = 0.8, 0.6H, CH py *E* isomer), 8.41 (ddd, ^3^*J*_H–H_ = 5.5, ^4^*J*_H–H_ = 1.7, ^5^*J*_H–H_ = 0.8, 0.3H, CH py *Z* isomer), 7.92 (m, 0.6H, CH arom *E* isomer), 7.86 (ddd, ^3^*J*_H–H_ = 7.0, ^4^*J*_H–H_ = 1.5, ^5^*J*_H–H_ = 0.8, 0.4H, CH py *Z* isomer), 7.73 (m, 1.8H, CH arom both isomers), 7.61 (m, 0.6H, CH arom *E* isomer), 7.52 (m, 4.0H, CH arom both isomers), 7.45 (m, 0.8H, CH arom *Z* isomer), 7.34 (ddd, ^3^*J*_H–H_ = 7.1, ^4^*J*_H–H_ = 1.6, ^5^*J*_H–H_ = 0.8, 0.6H, CH arom *E* isomer), 7.30 (ddd, ^3^*J*_H–H_ = 5.6, ^4^*J*_H–H_ = 1.7, ^5^*J*_H–H_ = 0.8, 0.4H, CH arom *Z* isomer), 7.12 (ddd, ^3^*J*_H–H_ = 7.1; 5.5 ^4^*J*_H–H_ = 1.3, 0.6H, CH arom *E* isomer), 7.04 (m, 2.2H, CH arom both isomers), 6.97 (m, 0.4H, CH arom *Z* isomer), 6.89 (m, 0.6H, CH arom *E* isomer), 6.79 (m, 2.8H, CH arom both isomers), 6.71 (m, 0.6H, CH arom *E* isomer), 6.63 (m, 1.6H, CH arom both isomers + =C*H*Ph *Z* isomer, inferred from the HMBC spectrum), 6.54 (m, 0.6H, CH MeC_6_H_3_-py *E* isomer), 6.47 (m, 1.0H, CH arom both isomers), 6.39 (m, 1.4H, CH arom both isomers), 6.21 (m, 0.3H, CH MeC_6_H_3_-py *Z* isomer), 5.99 (ddd, ^3^*J*_H–H_ = 7.1; 6.4, ^4^*J*_H–H_ = 1.4, 0.4H, CH py *Z* isomer), 5.77 (s, 0.6H, NH *E* isomer), 5.58 (ddd, ^3^*J*_H–H_ = 7.1; 6.4, ^4^*J*_H–H_ = 1.4, 0.3H, CH py *E* isomer), 5.44 (s, 0.4H, NH *Z* isomer), 4.93 (s, 0.6H, =C*H*Ph *E* isomer), 2.34 (s, 1.2H, CH_3_ MeC_6_H_3_-py *Z* isomer), 2.27 (s, 1.8H, CH_3_ MeC_6_H_3_-py *E* isomer), 2.06 (s, 1.8H, CH_3_ MeC_6_H_3_-py *E* isomer), 1.91 (s, 1.2H, CH_3_ MeC_6_H_3_-py *Z* isomer). ^13^C{^1^H} NMR (75 MHz, CD_2_Cl_2_, 298 K): δ 167.9, 167.8, 167.7, 167.2, 164.2 (s, C arom), 163.3 (s, 2C, C py), 161.8, 161.1, 161.0 (s, C arom), 150.0 (s, Ir–C–N *E* isomer), 149.8 (s, Ir–C–N *Z* isomer), 148.8, 148.4, 148.2, 148.0 (s, CH arom), 142.4, 142.4, 142.1, 142.0, 141.2, 140.4, 140.2, 139.5, 139.3 (s, C arom), 138.6 (s, CH arom), 138.3 (s, C arom), 138.1, 138.0, 137.7, 136.9, 136.6, 136.4, 136.3, 136.3, 136.1, 136.0. 133.8, 129.6, 129.2, 129.0, 128.7, 128.4, 126.3, 124.6, 124.4, 124.3, 124.3, 124.3, 124.2, 123.8, 121.8, 121.6, 121.4, (s, CH arom), 121.2 (s, CH), 121.1, 121.0, 120.9 (s, CH arom), 119.5 (s, CH), 119.1, 118.5, 118.4, 118.3, 118.1, 117.0 (s, CH arom), 105.9 (s, CH py *Z* isomer), 103.8 (s, CH py *E* isomer), 22.1 (s, CH_3_*Z* isomer), 22.0 (s, CH_3_*E* isomer), 21.9 (s, CH_3_*E* isomer), 21.9 (s, CH_3_*Z* isomer).

#### Isomerization of Ir{κ^2^-*C*,*N*-[C(=CHPh)-py-NH]}{κ^2^-*C*,*N*-(C_6_H_4_-Isoqui)}_2_ (**9**) to Ir{κ^2^-*C*,*N*-[C(CH_2_Ph)Npy]}{κ^2^-*C*,*N*-(C_6_H_4_-Isoqui)}_2_ (**11**)

A suspension of **9** (100 mg, 0.126 mmol) in toluene (7 mL) was stirred in a Schlenk flask, equipped with a PTFE stopcock, at 120 °C, for 7 days, and dried under vacuum. The resulting solid was passed through a silica column chromatograph using dichloromethane as an eluent to obtain the starting material and then using acetone to get **11** as an orange solid. Yield: 12 mg (12%). X-ray quality crystals were grown by layering a solution of this complex in toluene with pentane at 4 °C. Anal. Calcd for C_43_H_31_IrN_4_: C, 64.89; H, 3.93; N, 7.04. Found: C, 64.59; H, 6.51; N, 7.18. HRMS (electrospray, *m*/*z*) calcd for C_43_H_32_IrN_4_ [M + H]: 797.2251; found: 797.2262. ^1^H NMR (300 MHz, CD_2_Cl_2_, 298 K): δ 8.91 (m, 1H, CH Ph-isoqui), 8.73 (d, ^3^*J*_H–H_ = 8.7, 1H, CH Ph-isoqui), 8.18 (m, 1H, CH Ph-isoqui), 7.95 (d, ^3^*J*_H–H_ = 7.9, 1H, CH Ph-isoqui), 7.82 (dd, ^3^*J*_H–H_ = 7.9, ^4^*J*_H–H_ = 1.5, 1H, CH Ph-isoqui), 7.68 (m, 2H, CH py, 5H, CH Ph-isoqui), 7.40 (m, 1H, CH Ph-isoqui), 7.30 (q, ^3^*J*_H–H_ = 7.3, 2H, CH Ph-isoqui), 7.20 (ddd, ^3^*J*_H–H_ = 5.5, ^4^*J*_H–H_ = 1.8, ^5^*J*_H–H_ = 0.9, 1H, CH py), 7.06 (m, 5H, CH Ph-isoqui), 6.92 (ddd, ^3^*J*_H–H_ = 7.9; 7.1, ^4^*J*_H–H_ = 1.6, 1H, CH Ph-isoqui), 6.83 (td, ^3^*J*_H–H_ = 7.3, ^4^*J*_H–H_ = 4.2, CH Ph-isoqui), 6.73 (ddd, ^3^*J*_H–H_ = 7.1; 5.5, ^4^*J*_H–H_ = 1.6, 1H, CH py), 6.54 (m, 5H, CH C_6_H_5_), 4.02 (AB spin system, Δν = 41, *J*_A–B_ = 13.5, 2H, CH_2_). ^13^C{^1^H} NMR (75 MHz, CD_2_Cl_2_, 298 K): δ 228.2 (s, Ir–C=N), 171.6 (s, C py), 169.4 (s, C Ph-isoqui), 167.9 (s, C Ph-isoqui), 167.1 (s, C Ph-isoqui) 162.9 (s, C Ph-isoqui), 162.5 (s, C Ph-isoqui), 150.8 (s, C Ph-isoqui), 147.0 (s, C Ph-isoqui), 145.8 (s, CH py), 145.4 (s, C Ph-isoqui), 140.4 (s, CH Ph-isoqui), 139.2 (s, CH Ph-isoqui), 138.7 (s, CH py), 138.6 (s, CH Ph-isoqui), 138.5 (s, C C_6_H_5_), 137.7 (s, CH Ph-isoqui), 137.4 (s, C Ph-isoqui), 131.1 (s, CH Ph-isoqui), 131.0 (s, CH Ph-isoqui), 131.0 (s, CH Ph-isoqui), 130.9 (s, CH Ph-isoqui), 130.6 (s, CH Ph-isoqui), 130.0 (s, CH Ph-isoqui), 129.5 (s, CH C_6_H_5_), 128.4 (s, CH Ph-isoqui), 128.2 (s, CH Ph-isoqui), 128.1 (s, CH Ph-isoqui), 128.0 (s, CH Ph-isoqui), 127.6 (s, CH Ph-isoqui), 127.5 (s, CH Ph-isoqui), 127.3 (s, CH C_6_H_5_), 126.8 (s, C Ph-isoqui), 126.7 (s, C Ph-isoqui), 125.1 (s, CH C_6_H_5_), 121.1 (s, CH Ph-isoqui), 120.9 (s, CH Ph-isoqui), 120.7 (s, CH Ph-isoqui), 120.6 (s, CH Ph-isoqui), 119.2 (s, CH py), 118.9 (s, CH py), 54.9 (s, CH_2_).

#### Isomerization of Ir{κ^2^-*C*,*N*-[C(=CHPh)-py-NH]}{κ^2^-*C*,*N*-(MeC_6_H_3_-py)}_2_ (**10**) to Ir{κ^2^-*C*,*N*-[C(CH_2_Ph)Npy]}{κ^2^-*C*,*N*-(MeC_6_H_3_-py)}_2_ (**12**)

A suspension of **10** (100 mg, 0.138 mmol) in toluene (7 mL) was stirred in a Schlenk flask, equipped with a PTFE stopcock, at 120 °C, for 7 days, and dried under vacuum. The resulting solid was passed through a silica column chromatograph using dichloromethane as an eluent to obtain the starting material and then using acetone to get **12** as a yellow solid. Yield: 30 mg (10%). Anal. Calcd for C_37_H_31_IrN_4_: C, 61.39; H, 4.32; N, 7.74. Found: C, 61.47; H, 4.65, N, 7.87. HRMS (electrospray, *m*/*z*): Calcd for C_37_H_32_IrN_4_ [M + H]: 797.2251; found: 797.2262. ^1^H NMR (400 MHz, CD_2_Cl_2_, 298 K): δ 7.83 (d, ^3^*J*_H–H_ = 8.2, 1H, CH py), 7.59 (m, 6H, CH C_6_H_5_, py and MeC_6_H_3_-py), 7.34 (m, 2H, CH py), 7.27 (d, ^3^*J*_H–H_ = 7.8, 1H, MeC_6_H_3_-py), 7.19 (ddd, ^3^*J*_H–H_ = 5.5, ^4^*J*_H–H_ = 1.5, ^5^*J*_H–H_ = 0.7, 1H, CH py), 7.01 (s, 1H, CH MeC_6_H_3_-py), 6.81 (m, 7H, C_6_H_5_, py and MeC_6_H_3_-py), 6.70 (m, 2H, CH MeC_6_H_3_-py), 6.49 (d, ^3^*J*_H–H_ = 7.3, 2H, CH C_6_H_5_), 4.04 (AB spin system, Δν = 47, *J*_A–B_ = 13.1, 2H, CH_2_), 2.29 (s, 3H, CH_3_ MeC_6_H_3_-py), 2.13 (s, 3H, CH_3_ MeC_6_H_3_-py). ^13^C{^1^H} NMR (75 MHz, CD_2_Cl_2_, 298 K): δ 228.9 (s, Ir–C=N), 171.0 (s, N–C py), 167.1 (s, N–C py), 166.1 (s, N–C py), 158.8 (s, C MeC_6_H_3_-py, inferred from the HMBC spectrum), 158.3 (s, C MeC_6_H_3_-py, inferred from the HMBC spectrum), 148.1, 146.7, 145.6 (s, CH arom), 143.0 (s, Ir–C MeC_6_H_3_-py), 141.2 (s, Ir–C MeC_6_H_3_-py), 140.9 (s, C MeC_6_H_3_-py), 139.8 (s, C MeC_6_H_3_-py), 139.1 (s, CH MeC_6_H_3_-py), 138.6 (s, CH arom), 138.2 (s, CH MeC_6_H_3_-py), 137.8 (s, C C_6_H_5_, inferred from the HMBC spectrum), 137.1, 136.9 (s, CH arom), 129.8 (s, 2C, CH C_6_H_5_), 127.6 (s, 2C, CH C_6_H_5_), 125.1, 124.6, 124.4, 122.3, 122.2, 122.1, 122.0, 119.3, 119.1, 118.7 (s, CH arom), 55.0 (s, CH_2_), 22.0 (s, CH_3_ MeC_6_H_3_-py), 22.0 (s, CH_3_ MeC_6_H_3_-py).

#### Preparation of Ir{κ^2^-*C*,*N*-[C(CH_2_*^t^*Bu)Npy]}{κ^2^-*C*,*N*-(MeC_6_H_3_-py)}_2_ (**13**)

To a suspension of **8** (600 mg, 0.492 mmol) in toluene (30 mL), placed in a Schlenk flask equipped with a PTFE stopcock, was added 2-aminopyridine (140 mg, 1.487 mmol). The mixture was held during 24 h, at 120 °C. After that time, the orange solution was concentrated until approximately 2 mL, and pentane was added. The formed yellow solid was washed with pentane (3 × 3 mL) and dried under vacuum. Yield: 381 mg (55%). Anal. Calcd for C_35_H_35_IrN_4_: C, 59.72; H, 5.01; N, 7.96. Found: C, 59.63; H, 4.75; N, 7.97. HRMS (electrospray, *m*/*z*): Calcd for C_35_H_36_IrN_4_ [M + H]: 705.2564; found: 705.2565. ^1^H NMR (300 MHz, CD_2_Cl_2_, 298 K): δ 7.90 (d, ^3^*J*_H–H_ = 8.2, 1H, CH py), 7.81 (d, ^3^*J*_H–H_ = 8.2, 2H, CH py), 7.69 (ddd, ^3^*J*_H–H_ = 8.2; 7.4, ^4^*J*_H–H_ = 1.65, 1H, CH py), 7.56 (m, 5H, CH py, CH MeC_6_H_3_-py), 7.45 (d, ^3^*J*_H–H_ = 5.5, 1H, CH py), 7.31 (dt, ^3^*J*_H–H_ = 5.5, ^4^*J*_H–H_ = 1.2, 1H, CH py), 7.22 (d, ^3^*J*_H–H_ = 5.2, 1H, CH py), 7.04 (s, 1H, CH MeC_6_H_3_-py), 6.95 (ddd, ^3^*J*_H–H_ = 7.1; 5.5, ^4^*J*_H–H_ = 1.3, 1H, CH py), 6.84 (ddd, ^3^*J*_H–H_ = 7.0; 5.2, ^4^*J*_H–H_ = 1.3, 1H, CH py), 6.75 (m, 3H, CH py, CH MeC_6_H_3_-py), 6.68 (s, 1H, CH MeC_6_H_3_-py), 2.66 (AB spin system, Δν = 36, *J*_A–B_ = 14.8, 2H, CH_2_), 2.28 (s, 3H, CH_3_ MeC_6_H_3_-py), 2.09 (s, 3H, CH_3_ MeC_6_H_3_-py), 0.69 (s, 9H, *^t^*Bu). ^13^C{^1^H} NMR (75 MHz, CD_2_Cl_2_, 298 K): δ 234.5 (s, Ir–C=N), 172.4 (s, N–C py), 167.6 (s, N–C py), 166.1 (s, N–C py), 162.6 (s, C MeC_6_H_3_-py), 159.2 (s, C MeC_6_H_3_-py), 148.4 (s, CH py), 146.7 (s, CH py), 145.3 (s, CH py), 142.3 (s, Ir–C MeC_6_H_3_-py), 141.2 (s, Ir–C MeC_6_H_3_-py), 140.7 (s, C MeC_6_H_3_-py), 139.9 (s, C MeC_6_H_3_-py), 139.0 (s, CH MeC_6_H_3_-py), 138.4 (s, CH py), 138.2 (s, CH MeC_6_H_3_-py), 137.2 (s, CH py), 137.0 (s, CH py), 124.5 (s, CH MeC_6_H_3_-py), 124.3 (s, CH MeC_6_H_3_-py), 122.3 (s, CH py), 122.1 (s, CH MeC_6_H_3_-py), 122.0 (s, CH MeC_6_H_3_-py), 119.3 (s, CH py), 119.0 (s, CH py), 118.4 (s, CH py), 118.2 (s, CH py), 59.9 (s, CH_2_), 32.9 (s, C *^t^*Bu), 30.5 (s, CH_3_*^t^*Bu), 22.0 (s, CH_3_ MeC_6_H_3_-py), 22.0 (s, CH_3_ MeC_6_H_3_-py).

#### Preparation of PMMA films

An amount of 19 mg of PMMA (average *M*_w_ 97 000, average *M*_n_ 46 000) was dissolved in 1.0 mL of dichloromethane in a glovebox at room temperature. Then, 1 mg of the iridium complex (5 wt %) was added with stirring to form a homogeneous solution, which was drop-coated onto a quartz substrate and dried at room temperature.

## Supporting Information Available

General information for the experimental section, Eyring plots, structural analysis, and NMR spectra (PDF)
*xyz* coordinates (XYZ)

## Supplementary Material

ic2c00197_si_001.pdf

ic2c00197_si_002.xyz

## Accession Codes

CCDC 2141479–2141485 contain the supplementary crystallographic data for this paper. These data can be obtained free of charge via www.ccdc.cam.ac.uk/data_request/cif, or by emailing data_request@ccdc.cam.ac.uk, or by contacting The Cambridge Crystallographic Data Centre, 12 Union Road, Cambridge CB2 1EZ, UK; fax: +44 1223 336033.
