# Supplementary material for: Alkynyl Ligands as Building Blocks for the Preparation of Phosphorescent Iridium(III) Emitters: Alternative Synthetic Precursors and Procedures
Source: Inorg Chem. 2022 Apr 19;61(24):9019–33. doi: 10.1021/acs.inorgchem.2c00197 (PMC9490848; doi:10.1021/acs.inorgchem.2c00197)
Supplement: Supplementary file 1 — ic2c00197_si_001.pdf [file ic2c00197_si_001.pdf]

Supporting Information For

Alkynyl Ligands as Building Blocks for the Preparation of Phosphorescent Iridium(III) Emitters: Alternative Synthetic Precursors and Procedures

Vadim Adamovich,[2] María Benítez,[1] Pierre-Luc Boudreault,[2] María L. Buil,[1] Miguel A. Esteruelas,[1] Enrique Oñate,[1] and Jui-Yi Tsai[2]*

[1]Departamento de Química Inorgánica, Instituto de Síntesis Química y Catálisis Homogénea (ISQCH), Centro de Innovación en Química Avanzada (ORFEO-CINQA), Universidad de Zaragoza – CSIC, 50009 Zaragoza, Spain

[2]Universal Display Corporation, Ewing, New Jersey 08618, United States

*Corresponding author's e-mail address: maester@unizar.es

CONTENTS

Experimental section: Instrumental methods S3

Structural Analysis of Complexes 3, 4, 6, 7, 9, 10 and 11 S4

Computational Details S7

Energies of Optimized Structures of 11-13 S7

UV-vis Spectra of Complexes 11-13 (Observed and Calculated) S9

Analysis of Computed UV/Vis Data of Complexes 11-13 S11

Theoretical Analysis of Molecular Orbitals of Complexes 11-13 S14

Spin Density Distributions for the Optimized Triplet T_1 of 11-13 S20

Cyclic Voltammograms of Complexes 11-13 S20

Normalized Excitation and Emission Spectra of Complexes 11-13 S21

NMR spectra S24

References S49

Experimental section: Instrumental methods. Solvents were dried by the usual procedures and distilled under argon atmosphere or from an MBraun solvent purification apparatus. NMR spectra were recorded on a Bruker ARX 300, Bruker Avance 300 MHz, Bruker Avance 400 MHz or Bruker Avance 500 MHz instruments. Elemental analyses were carried out in a Perkin-Elmer 2400-B Series II CHNS-Analyzer. High-resolution electrospray (HRMS) mass spectra were acquired using a MicroTOF-Q hybrid quadrupole time-of-flight spectrometer (Bruker Daltonics, Bremen, Germany). Attenuated total reflection infrared spectra (ATR-IR) of solid samples were run on a Perkin-Elmer Spectrum 100 FT-IR spectrometer. UV-visible spectra were registered on an Evolution 600 spectrophotomer. Steady-state photoluminescence spectra were recorded on a Jobin-Yvon Horiba Fluorolog FL-3-11 spectrofluorometer. Lifetimes were measured using an IBH 5000F coaxial nanosecond flash lamp. Quantum yields were measured using the Hamamatsu Absolute PL Quantum Yield Measurement System C11347-11. Cyclic voltammetry measurements were performed using a Voltalab PST050 potentiostat with Pt wire as working electrode, Pt wire as counter electrode, and saturated calomel (SCE) as reference electrode. The experiments were carried out under argon in dicloromethane solutions (10^{-3} M), with Bu_4NPF_6 as supporting electrolyte (0.1 M). Scan rate was 100 mV·s-1. The potentials were referenced to the ferrocene/ferrocenium (Fc/Fc^+) couple. The emitter **13** have been tested in bottom emission OLED structures with the device pixel area 2 mm^2. The glass coated ITO substrates were patterned by photolithography. The device pixel area was defined by the photoresist grid. The devices were fabricated by high vacuum (<10^{-7} Torr) thermal evaporation (VTE). Organic layers have been deposited with a deposition rate of 2 Å/s. Devices electroluminescence spectra and luminance were tested using a Spectrophotometer PR-730 at 10 mA/cm^2. Current-voltage-luminance (JVL) measurements were performed using calibrated photodiode.

Structural Analysis of Complexes 3, 4, 6, 7, 9, 10 and 11. X-ray data were collected on a Bruker APEX CCD (**3, 4, 6, 7, 11**), and DUO CCD (**9, 10**) diffractometers (Mo radiation, λ = 0.71073 Å). The crystals were cooled with a nitrogen flow from Oxford Cryosystems systems. Data were corrected for absorption by using a multiscan method applied with the SADABS program.[1] The structures were solved by Patterson or direct methods and refined by full-matrix least squares on F^2 with SHELXL2016,[2] including isotropic and subsequently anisotropic displacement parameters. The hydrogen atoms were observed in the last Fourier Maps or calculated, and refined freely or using a restricted riding model.

Crystal data for **3** (CSD deposition number 2141479): $C_{76}H_{50}Ir_2N_4$ x CH_2Cl_2, M_W 1488.52, red, irregular block (0.187 x 0.086 x 0.036 mm^3), triclinic, space group P-1, *a*: 11.7330(6) Å, *b*: 14.2956(8) Å, *c*: 18.0139(10) Å, α: 75.9440(10) °, β: 81.7670(10) °, γ: 78.5260(10) °, *V* = 2858.1(3) $Å^3$, *Z* = 2, *Z'* = 1, D_{calc}: 1.730 g cm^{-3}, F(000): 1460, T = 100(2) K, μ 4.797 mm^{-1}. 30126 measured reflections (2θ: 3-57°, ω scans 0.3^o), 13306 unique (R_{int} = 0.0321); min./max. transm. factors 0.669/0.862. Final agreement factors were R^1 = 0.0324 (10522 observed reflections, I > 2σ(I)) and wR^2 = 0.0755; data/restraints/parameters 13306/0/766; GoF = 1.028. Largest peak and hole 1.593 (close to iridium atoms) and -1.389 e/ $Å^3$.

Crystal data for **4** (CSD deposition number 2141483): $C_{64}H_{50}Ir_2N_4$ x 2(C_7H_8), M_W 1443.74, yellow, irregular block (0.300 x 0.050 x 0.040 mm^3), triclinic, space group P-1, *a*: 14.0985(14) Å, *b*: 14.3987(14) Å, *c*: 16.3805(16) Å, α: 97.0440(10) °, β: 103.5210(10) °, γ: 107.9650(10) °, *V* = 3006.5(5) $Å^3$, *Z* = 2, *Z'* = 1, D_{calc}: 1.595 g cm^{-3}, F(000): 1432, T = 150(2) K, μ 4.471 mm^{-1}. 52874 measured reflections (2θ: 3-57°, ω scans 0.3^o), 14359 unique (R_{int} = 0.0467); min./max. transm. factors 0.592/0.862. Final agreement factors were R^1 = 0.0309 (11083 observed reflections, I > 2σ(I)) and wR^2 = 0.0712;

data/restraints/parameters 14359/10/732; GoF = 1.027. Largest peak and hole 2.332 (close to iridium atoms) and -1.133 e/ Å^3.

Crystal data for **6** (CSD deposition number 2141481): $C_{76}H_{50}Ir_2N_4$ x 3(CH_2Cl_2), M_W 1658.37, yellow, irregular block (0.183 x 0.126 x 0.108 mm^3), monoclinic, space group P2/n, *a*: 14.1431(9) Å, *b*: 15.4188(10) Å, *c*: 15.4571(10) Å, *β*: 108.2720(10) °, *V* = 3200.8(4) Å^3, *Z* = 2, *Z'* = 0.5, D_{calc}: 1.721 g cm^{-3}, F(000): 1628, T = 100(2) K, μ 4.455 mm^{-1}. 55018 measured reflections (2θ: 3-57°, ω scans 0.3^o), 7864 unique (R_{int} = 0.0563); min./max. transm. factors 0.675/0.862. Final agreement factors were R^1 = 0.0278 (6158 observed reflections, I > 2σ(I)) and wR^2 = 0.0655; data/restraints/parameters 7864/0/411; GoF = 1.033. Largest peak and hole 1.814 (close to iridium atoms) and -1.501 e/ Å^3.

Crystal data for **7** (CSD deposition number 2141480): $C_{64}H_{50}Ir_2N_4$ x 0.5(C_7H_8) x 0.5(CH_2Cl_2), M_W 1348.01, yellow, irregular block (0.173 x 0.112 x 0.075 mm^3), triclinic, space group P-1, *a*: 12.8924(11) Å, *b*: 14.3214(12) Å, *c*: 15.2084(13) Å, *α*: 87.3910(10) °, *β*: 77.4950(10) °, *γ*: 77.0800(10) °, *V* = 2672.0(4) Å^3, *Z* = 2, *Z'* = 1, D_{calc}: 1.675 g cm^{-3}, F(000): 1324, T = 100(2) K, μ 5.073 mm^{-1}. 41043 measured reflections (2θ: 3-57°, ω scans 0.3^o), 12649 unique (R_{int} = 0.0518); min./max. transm. factors 0.668/0.862. Final agreement factors were R^1 = 0.0455 (9635 observed reflections, I > 2σ(I)) and wR^2 = 0.1183; data/restraints/parameters 12649/3/643; GoF = 1.039. Largest peak and hole 3.326 (close to iridium atoms) and -2.249 e/ Å^3.

Crystal data for **9** (CSD deposition number 2141485): $C_{43}H_{31}IrN_4$, M_W 795.92, red, irregular block (0.300 x 0.040 x 0.030 mm^3), monoclinic, space group $P2_1/c$, *a*: 11.2451(13) Å, *b*: 13.7323(16) Å, *c*: 21.453(3) Å, *β*: 101.707(2) °, *V* = 3243.9(7) Å^3, *Z* = 4, *Z'* = 1, D_{calc}: 1.630 g cm^{-3}, F(000): 1576, T = 120(2) K, μ 4.155 mm^{-1}. 32866 measured reflections (2θ: 3-57°, ω scans 0.3^o), 9363 unique (R_{int} = 0.0633); min./max. transm. factors 0.677/0.862. Final agreement factors were R^1 = 0.0336 (6439 observed reflections,

I > 2σ(I)) and wR^2 = 0.0673; data/restraints/parameters 9363/0/436; GoF = 1.009. Largest peak and hole 1.009 (close to iridium atoms) and -1.714 e/ $Å^3$.

Crystal data for **10** (CSD deposition number 2141484): $C_{37}H_{31}IrN_4$, M_W 723.86, red, irregular block (0.210 x 0.086 x 0.073 mm^3), monoclinic, space group $P2_1/n$, *a*: 10.7154(13) Å, *b*: 21.539(3) Å, *c*: 13.1315(16) Å, *β*: 106.479(2) °, *V* = 2906.2(6) $Å^3$, *Z* = 4, *Z'* = 1, D_{calc}: 1.654 g cm^{-3}, F(000): 1432, T = 100(2) K, μ 4.628 mm^{-1}. 46231 measured reflections (2θ: 3-57°, ω scans 0.3°), 7936 unique (R_{int} = 0.0383); min./max. transm. factors 0.668/0.862. Final agreement factors were R^1 = 0.0223 (6723 observed reflections, I > 2σ(I)) and wR^2 = 0.0511; data/restraints/parameters 7936/0/387; GoF = 1.001. Largest peak and hole 1.293 (close to iridium atoms) and -0.598 e/ $Å^3$.

Crystal data for **11** (CSD deposition number 2141482): $C_{43}H_{31}IrN_4$ x 0.25(C_5H_{12}), M_W 813.95, red, irregular block (0.109 x 0.108 x 0.094 mm^3), monoclinic, space group $P2_1/c$, *a*: 19.549(2) Å, *b*: 17.2406(18) Å, *c*: 20.010(2) Å, *β*: 93.058(2) °, *V* = 6734.6(12) $Å^3$, *Z* = 8, *Z'* = 2, D_{calc}: 1.606 g cm^{-3}, F(000): 3236, T = 100(2) K, μ 4.004 mm^{-1}. 66691 measured reflections (2θ: 3-57°, ω scans 0.3°), 12524 unique (R_{int} = 0.0935); min./max. transm. factors 0.600/0.862. Final agreement factors were R^1 = 0.0566 (9240 observed reflections, I > 2σ(I)) and wR^2 = 0.1551; data/restraints/parameters 12524/7/881; GoF = 1.041. Largest peak and hole 5.397 (close to iridium atoms) and -3.252 e/ $Å^3$.

Computational Details. All calculations were performed at the DFT level using the B3LYP functional[3] supplemented with the Grimme's dispersion correction D3[4] as implemented in Gaussian09.[5] Ir atoms were described by means of an effective core potential SDD for the inner electron[6] and its associated double-ζ basis set for the outer ones, complemented with a set of f-polarization functions for iridium.[7] The 6-31G** basis set was used for the H, C, and N atoms.[8] All minima were verified to have no negative frequencies. The geometries were fully optimized in vacuo and in THF ($\varepsilon = 7.4257$) solvent using the continuum SMD model.[9] We performed TD-DFT calculations at the same level of theory in THF calculating the lowest 50 singlet-singlet excitations at the ground state S_0. It should be noted that the singlet-triplet excitations are set to zero due to the neglect of spin-orbit coupling in the TDDFT calculations as implemented in G09. The UV/vis absorption spectra were obtained by using the GaussSum 3 software.[10] The phosphorescence emission compares well with the 0-0 transition calculated taking into account the zero point energies (zpe) of the geometries of both the optimized T_1 and S_0 states in THF.

Energies of Optimized Structures of 11-13

Complex 11 S_0(thf)

Zero-point correction=	0.626049 (Hartree/Particle)
Thermal correction to Energy=	0.662935
Thermal correction to Enthalpy=	0.663879
Thermal correction to Gibbs Free Energy=	0.556739
Sum of electronic and zero-point Energies=	-1980.361406
Sum of electronic and thermal Energies=	-1980.324520
Sum of electronic and thermal Enthalpies=	-1980.323575
Sum of electronic and thermal Free Energies=	-1980.430715

Complex 11 T_1(thf)

Zero-point correction=	0.622495 (Hartree/Particle)
Thermal correction to Energy=	0.659810
Thermal correction to Enthalpy=	0.660754
Thermal correction to Gibbs Free Energy=	0.551391

Sum of electronic and zero-point Energies= -1980.287853
Sum of electronic and thermal Energies= -1980.250539
Sum of electronic and thermal Enthalpies= -1980.249594
Sum of electronic and thermal Free Energies= -1980.358957

Complex 12 S_0(thf)

Zero-point correction= 0.586440 (Hartree/Particle)
Thermal correction to Energy= 0.621785
Thermal correction to Enthalpy= 0.622729
Thermal correction to Gibbs Free Energy= 0.518778
Sum of electronic and zero-point Energies= -1751.752749
Sum of electronic and thermal Energies= -1751.717404
Sum of electronic and thermal Enthalpies= -1751.716460
Sum of electronic and thermal Free Energies= -1751.820411

Complex 12 T_1(thf)

Zero-point correction= 0.582372 (Hartree/Particle)
Thermal correction to Energy= 0.618363
Thermal correction to Enthalpy= 0.619307
Thermal correction to Gibbs Free Energy= 0.513241
Sum of electronic and zero-point Energies= -1751.661429
Sum of electronic and thermal Energies= -1751.625437
Sum of electronic and thermal Enthalpies= -1751.624493
Sum of electronic and thermal Free Energies= -1751.730559

Complex 13 S_0(thf)

Zero-point correction= 0.618116 (Hartree/Particle)
Thermal correction to Energy= 0.653493
Thermal correction to Enthalpy= 0.654437
Thermal correction to Gibbs Free Energy= 0.551613
Sum of electronic and zero-point Energies= -1677.923127
Sum of electronic and thermal Energies= -1677.887751
Sum of electronic and thermal Enthalpies= -1677.886806
Sum of electronic and thermal Free Energies= -1677.989631

Complex 13 T_1 (thf)

Zero-point correction= 0.614038 (Hartree/Particle)
Thermal correction to Energy= 0.651196
Thermal correction to Enthalpy= 0.652140
Thermal correction to Gibbs Free Energy= 0.542140
Sum of electronic and zero-point Energies= -1677.828273
Sum of electronic and thermal Energies= -1677.791115
Sum of electronic and thermal Enthalpies= -1677.790171
Sum of electronic and thermal Free Energies= -1677.900172

UV-vis Spectra of Complexes 11-13 (Observed and Calculated)

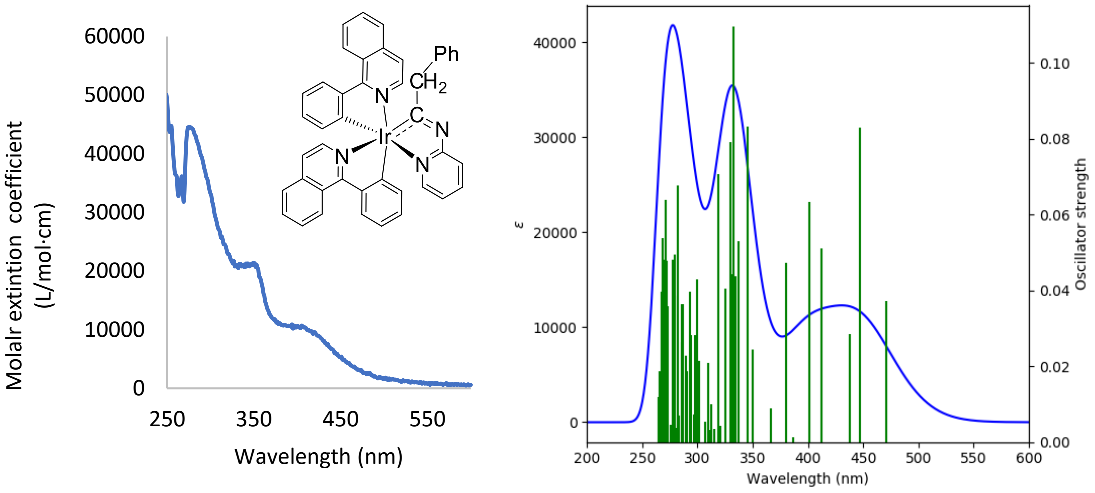

Figure S1. Observed UV-vis spectrum of complex **11** in 2-MeTHF (1.0 x 10^{-5} M) and calculated (B3LYP(GD3)//SDD(f)/6-31G**) in THF.

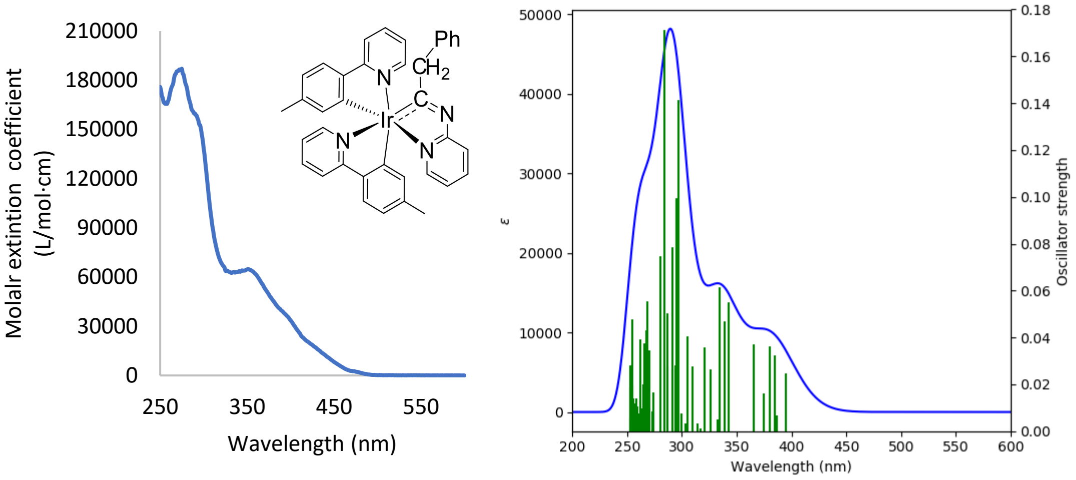

Figure S2. Observed UV-vis spectrum of complex **12** in 2-MeTHF (1.0 x 10^{-5} M) and calculated (B3LYP(GD3)//SDD(f)/6-31G**) in THF.

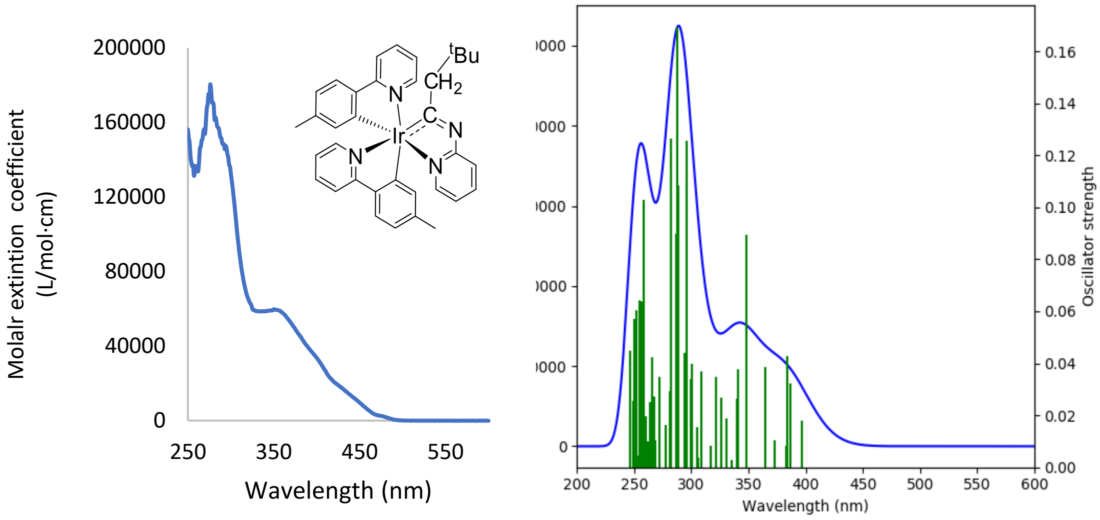

Figure S3. Observed UV-vis spectrum of complex **13** in 2-MeTHF (1.0 x 10^{-5} M) and calculated (B3LYP(GD3)//SDD(f)/6-31G**) in THF.

Analysis of Computed UV/Vis Data of Complexes 11-13

Table S1. Selected transitions for the calculated UV spectrum of complex 11 in THF

λ (nm)	Osc. Strength	Symmetry	Major contributions	Minor contributions
554	0	Triplet-A	H-4->LUMO (10%), HOMO->LUMO (59%)	H-3->L+1 (9%), H-2->L+1 (8%), H-1->L+1 (5%)
471	0.0373	Singlet-A	HOMO->LUMO (95%)	H-1->LUMO (2%)
447	0.0828	Singlet-A	H-1->LUMO (66%), HOMO->L+1 (30%)	-
401	0.0634	Singlet-A	H-2->LUMO (88%)	H-2->L+1 (4%), H-1->L+1 (3%)
345	0.0831	Singlet-A	H-4->LUMO (87%)	H-5->LUMO (6%)
333	0.1097	Singlet-A	H-3->L+1 (56%), H-2->L+2 (22%)	H-2->L+1 (3%), HOMO->L+3 (8%)
318	0.0706	Singlet-A	H-6->LUMO (12%), H-5->L+1 (38%), H-1->L+3 (14%), HOMO->L+4 (16%)	H-4->L+1 (5%), HOMO->L+3 (4%)
283	0.0678	Singlet-A	H-2->L+5 (64%)	H-11->LUMO (3%), H-10->L+1 (3%), H-9->L+1 (2%), H-4->L+2 (2%), HOMO->L+6 (9%)
272	0.0638	Singlet-A	H-13->LUMO (34%), H-10->L+1 (10%), H-3->L+3 (20%)	H-12->L+1 (4%), H-11->L+1 (4%), H-1->L+6 (6%), H-1->L+7 (2%), HOMO->L+6 (7%)
268	0.0538	Singlet-A	H-6->L+2 (74%), H-5->L+2 (15%)	-

Table S2. Composition (%) of the selected transitions for the calculated UV-vis spectrum of complex 11

λ (nm)	Osc. Strength	Symmetry	Ir	Ph-isoqui(1)	Ph-isoqui(2)	Metallacycle
554	0	Triplet-A	35-->2 (-33)	22-->50 (28)	32-->46 (14)	11-->2 (-9)
471	0.0373	Singlet-A	43-->2 (-41)	17-->52 (35)	33-->44 (11)	7-->2 (-5)
447	0.0828	Singlet-A	42-->3 (-39)	29-->49 (20)	22-->46 (24)	6-->2 (-4)
401	0.0634	Singlet-A	39-->2 (-37)	12-->51 (39)	22-->45 (23)	27-->2 (-25)
345	0.0831	Singlet-A	7-->2 (-5)	53-->52 (-1)	20-->44 (24)	19-->2 (-17)
333	0.1097	Singlet-A	22-->4 (-18)	20-->35 (15)	41-->38 (-3)	18-->22 (4)
318	0.0706	Singlet-A	24-->3 (-21)	20-->47 (27)	16-->48 (32)	40-->2 (-38)
283	0.0678	Singlet-A	36-->3 (-33)	15-->14 (-1)	24-->9 (-15)	25-->74 (49)
272	0.0638	Singlet-A	16-->3 (-13)	31-->53 (22)	39-->43 (4)	14-->2 (-12)
268	0.0538	Singlet-A	7-->8 (1)	7-->2 (-5)	6-->5 (-1)	80-->86 (6)

Table S3. Selected transitions for the calculated UV spectrum of complex 12 in THF

λ (nm)	Osc. Strength	Symmetry	Major contributions	Minor contributions
452	0	Triplet-A	HOMO->LUMO (37%), HOMO->L+1 (26%)	H-3->LUMO (4%), H-3->L+1 (6%), H-2->L+1 (3%), H-1->L+1 (5%), HOMO->L+2 (4%)
395	0.0245	Singlet-A	HOMO->LUMO (85%)	H-1->LUMO (8%), HOMO->L+2 (4%)
381	0.0364	Singlet-A	H-1->LUMO (54%), H-1->L+2 (13%), HOMO->L+1 (19%)	H-1->L+1 (3%), HOMO->LUMO (5%)
365	0.0371	Singlet-A	H-1->L+1 (71%), HOMO->L+1 (13%)	H-1->L+2 (6%), HOMO->L+3 (2%)
343	0.055	Singlet-A	H-2->LUMO (84%)	H-1->L+2 (6%), HOMO->L+3 (2%)
335	0.0613	Singlet-A	H-2->L+1 (72%), H-2->L+2 (16%)	HOMO->L+3 (2%)
297	0.1414	Singlet-A	H-3->L+1 (66%), H-2->L+3 (12%)	H-5->LUMO (4%), H-3->L+2 (3%), H-2->L+4 (8%)
284	0.1714	Singlet-A	H-2->L+4 (67%)	H-5->L+1 (7%), H-4->LUMO (3%), H-3->L+1 (4%), H-2->L+5 (3%)
262	0.394	Singlet-A	H-3->L+4 (71%)	H-8->LUMO (3%), H-8->L+1 (3%), H-6->L+1 (3%), H-4->L+4 (3%), HOMO->L+8 (2%)

Table S4. Composition (%) of the selected transitions for the calculated UV-vis spectrum of complex 12

λ (nm)	Osc. Strength	Symmetry	Ir	Ph-Py(1)	Ph-Py(2)	Metallacycle
452	0	Triplet-A	38-->3 (-35)	12-->45 (33)	41-->44 (3)	9-->7 (-2)
395	0.0245	Singlet-A	41-->2 (-39)	12-->49 (37)	39-->39 (0)	8-->10 (2)
381	0.0364	Singlet-A	42-->3 (-39)	32-->43 (11)	19-->38 (19)	7-->16 (9)
365	0.0371	Singlet-A	42-->5 (-37)	34-->41 (7)	16-->48 (32)	7-->6 (-1)
343	0.055	Singlet-A	42-->2 (-40)	11-->51 (40)	14-->40 (26)	33-->7 (-26)
335	0.0613	Singlet-A	43-->5 (-38)	11-->37 (26)	12-->43 (31)	35-->15 (-20)
297	0.1414	Singlet-A	17-->4 (-13)	12-->43 (31)	51-->48 (-3)	19-->5 (-14)
284	0.1714	Singlet-A	38-->3 (-35)	13-->35 (22)	13-->54 (41)	36-->7 (-29)
262	0.394	Singlet-A	11-->3 (-8)	15-->35 (20)	57-->55 (-2)	17-->7 (-10)

Table S5. Selected transitions for the calculated UV spectrum of complex 13 in THF

λ (nm)	Osc. Strength	Symmetry	Major contributions	Minor contributions
450	0	Triplet-A	H-1->LUMO (22%), H-1->L+1 (12%), HOMO->LUMO (36%)	H-5->LUMO (3%), H-3->LUMO (5%), H-3->L+1 (3%), H-2->LUMO (2%), H-2->L+1 (2%)
397	0.0181	Singlet-A	HOMO->LUMO (95%)	-
384	0.0428	Singlet-A	H-1->LUMO (70%), HOMO->L+1 (20%)	H-1->L+2 (3%), HOMO->L+2 (4%)
365	0.0385	Singlet-A	H-1->L+1 (83%)	H-2->L+2 (2%), H-1->L+2 (9%)
348	0.0896	Singlet-A	H-2->LUMO (86%)	H-2->L+1 (2%), H-2->L+2 (2%), H-1->L+3 (2%)
309	0.037	Singlet-A	H-2->L+3 (36%), HOMO->L+5 (47%)	H-3->LUMO (7%), H-2->L+2 (4%)
296	0.1255	Singlet-A	H-5->LUMO (43%), H-4->LUMO (12%), H-2->L+4 (31%)	H-3->L+1 (3%)
288	0.1694	Singlet-A	H-5->L+1 (62%), H-2->L+4 (11%)	H-5->LUMO (3%), H-4->L+1 (9%), H-4->L+3 (3%)
282	0.1265	Singlet-A	H-5->L+2 (15%), H-3->L+2 (17%), H-2->L+5 (56%)	H-2->L+4 (3%)
259	0.1028	Singlet-A	H-8->LUMO (11%), H-7->L+1 (28%), H-4->L+4 (21%)	H-8->L+1 (4%), H-6->LUMO (5%), H-6->L+2 (6%), H-3->L+4 (8%), HOMO->L+8 (3%)

Table S6. Composition (%) of the selected transitions for the calculated UV-vis spectrum of complex 13

λ (nm)	Osc. Strength	Symmetry	Ir	Ph-Py (1)	Ph-Py(2)	Metallacycle
450	0	Triplet-A	39-->3 (-36)	23-->35 (12)	27-->57 (30)	11-->6 (-5)
397	0.0181	Singlet-A	47-->2 (-45)	31-->27 (-4)	16-->64 (48)	6-->7 (1)
384	0.0428	Singlet-A	42-->3 (-39)	20-->34 (14)	30-->53 (23)	7-->10 (3)
365	0.0385	Singlet-A	41-->5 (-36)	17-->61 (44)	35-->25 (-10)	8-->9 (1)
348	0.0896	Singlet-A	40-->2 (-38)	11-->27 (16)	9-->62 (53)	39-->8 (-31)
309	0.037	Singlet-A	41-->4 (-37)	21-->21 (0)	15-->24 (9)	23-->51 (28)
296	0.1255	Singlet-A	22-->2 (-20)	25-->36 (11)	25-->56 (31)	28-->6 (-22)
288	0.1694	Singlet-A	17-->4 (-13)	28-->63 (35)	32-->31 (-1)	23-->2 (-21)
282	0.1265	Singlet-A	30-->5 (-25)	14-->7 (-7)	21-->5 (-16)	35-->82 (47)
259	0.1028	Singlet-A	20-->4 (-16)	33-->50 (17)	23-->39 (16)	23-->8 (-15)

Theoretical Analysis of Molecular Orbitals of Complexes 11-13

Energies and population analysis (%) of frontier molecular orbitals are given in Tables S7–S9 whereas Figures S4–S6 collects the frontier molecular orbitals.

Table S7. Composition of the frontier orbital of complex 11

MO	eV	Iridium	Ph-isoqui(1)	Ph-isoqui(2)	Metallacycle
L+9	0.23	66	8	4	21
L+8	0.13	14	1	16	69
L+7	-0.10	2	33	61	4
L+6	-0.16	3	63	32	2
L+5	-0.49	3	3	1	93
L+4	-0.72	2	39	57	1
L+3	-0.81	3	56	37	4
L+2	-1.10	8	2	5	86
L+1	-1.67	3	45	51	1
LUMO	-1.85	2	52	44	2
HOMO	-5.17	43	16	34	7
H-1	-5.29	42	35	17	6
H-2	-5.56	39	11	22	28
H-3	-5.90	12	24	50	15
H-4	-5.96	7	56	21	16
H-5	-6.15	15	16	9	60
H-6	-6.37	6	5	6	84
H-7	-6.45	4	2	17	77
H-8	-6.67	21	34	22	24
H-9	-6.69	16	4	20	60

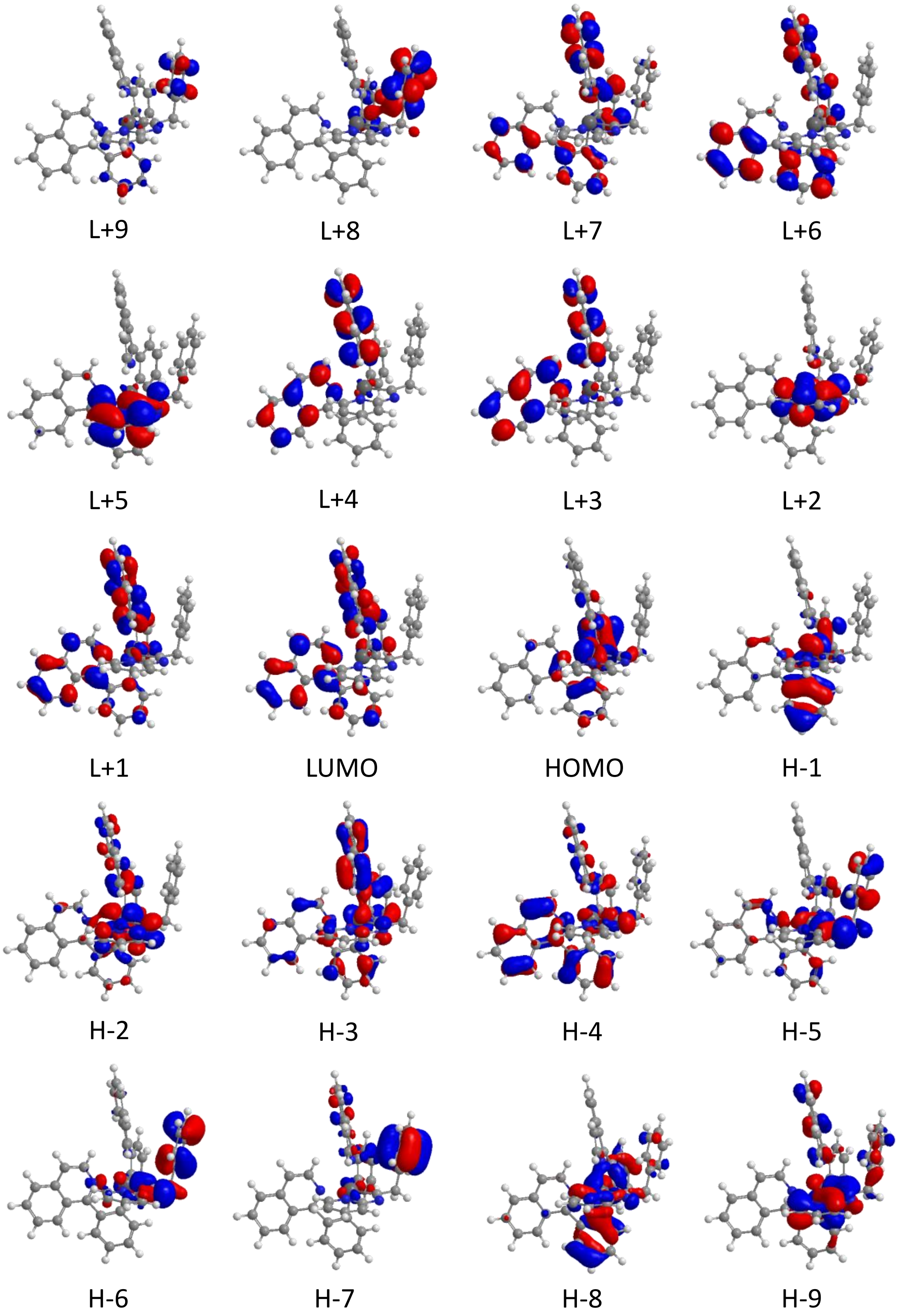

Figure S4. Frontier molecular orbitals of complex **11** (isovalue 0.003 au).

Table S8. Composition of the frontier orbital of complex 12

MO	eV	Iridium	Ph-Py(1)	Ph-py(2)	Metallacycle
L+9	0.52	8	75	10	7
L+8	0.27	5	1	1	93
L+7	0.20	84	2	3	11
L+6	0.10	9	1	14	76
L+5	-0.46	3	9	0	88
L+4	-0.59	3	34	58	5
L+3	-0.78	4	52	35	9
L+2	-1.07	8	5	9	79
L+1	-1.14	5	44	51	1
LUMO	-1.24	2	51	40	7
HOMO	-5.13	41	10	41	8
H-1	-5.20	43	39	11	7
H-2	-5.58	43	11	11	36
H-3	-5.89	10	13	65	12
H-4	-5.97	7	73	12	8
H-5	-6.10	16	13	6	65
H-6	-6.38	6	3	2	90
H-7	-6.53	4	2	10	84
H-8	-6.65	17	20	9	53
H-9	-6.71	25	19	29	27

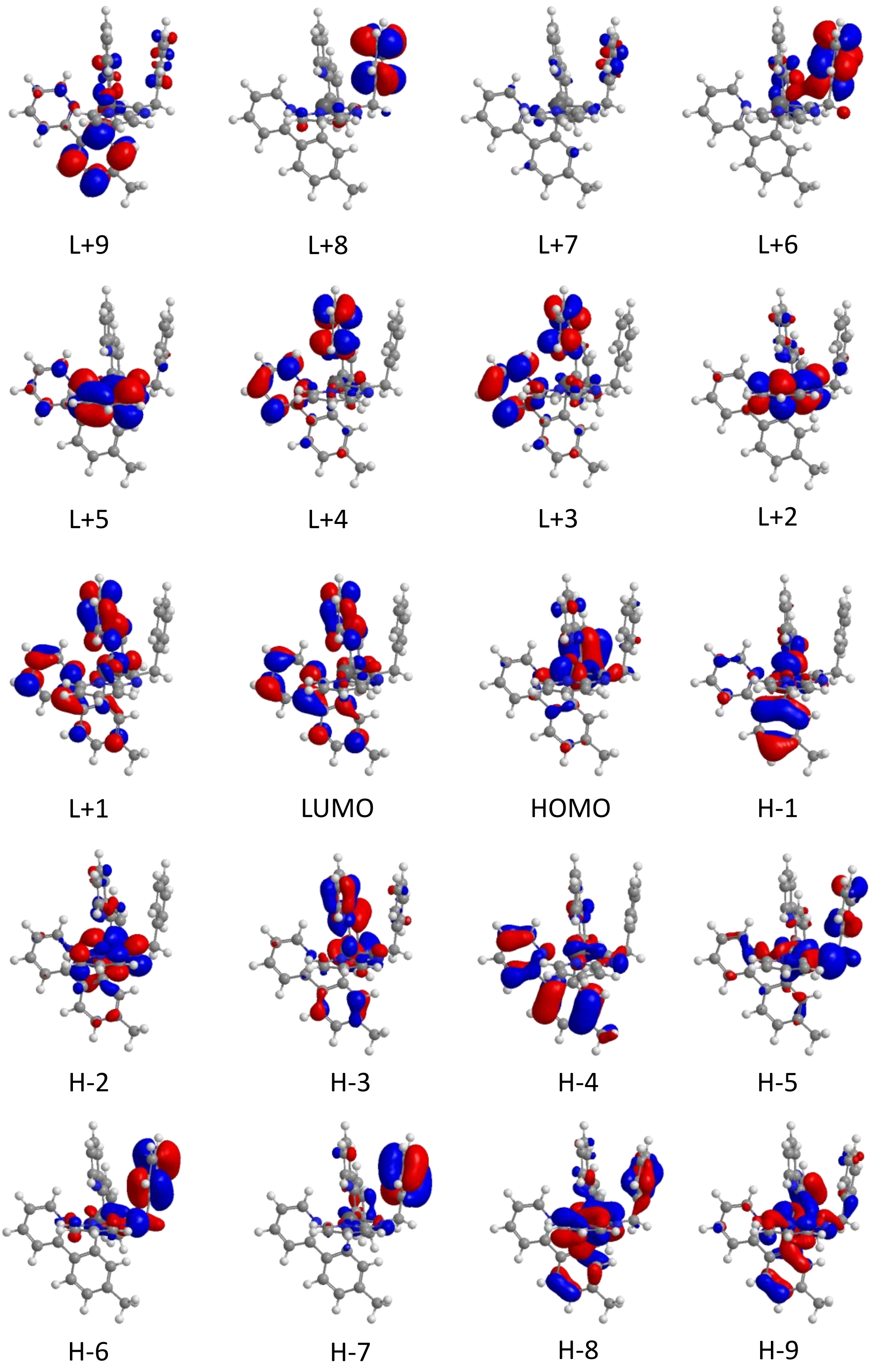

Figure S5. Frontier molecular orbitals of complex **12** (isovalue 0.003 au).

Table S9. Composition of the frontier orbital of complex 13

MO	eV	Iridium	Ph-Py(1)	Ph-py(2)	L
L+9	1.01	9	42	45	4
L+8	0.61	6	53	41	1
L+7	0.47	6	39	54	1
L+6	0.17	94	1	1	3
L+5	-0.42	4	7	0	89
L+4	-0.61	3	50	43	4
L+3	-0.81	5	38	48	10
L+2	-1.03	9	4	11	77
L+1	-1.15	5	68	27	1
LUMO	-1.27	2	27	64	7
HOMO	-5.15	47	31	16	6
H-1	-5.19	41	17	35	7
H-2	-5.52	40	11	9	40
H-3	-5.92	9	13	46	32
H-4	-5.93	10	62	3	25
H-5	-6.03	14	24	42	20
H-6	-6.53	25	8	8	58
H-7	-6.68	25	32	32	12
H-8	-6.80	25	26	34	15
H-9	-6.93	14	23	42	21

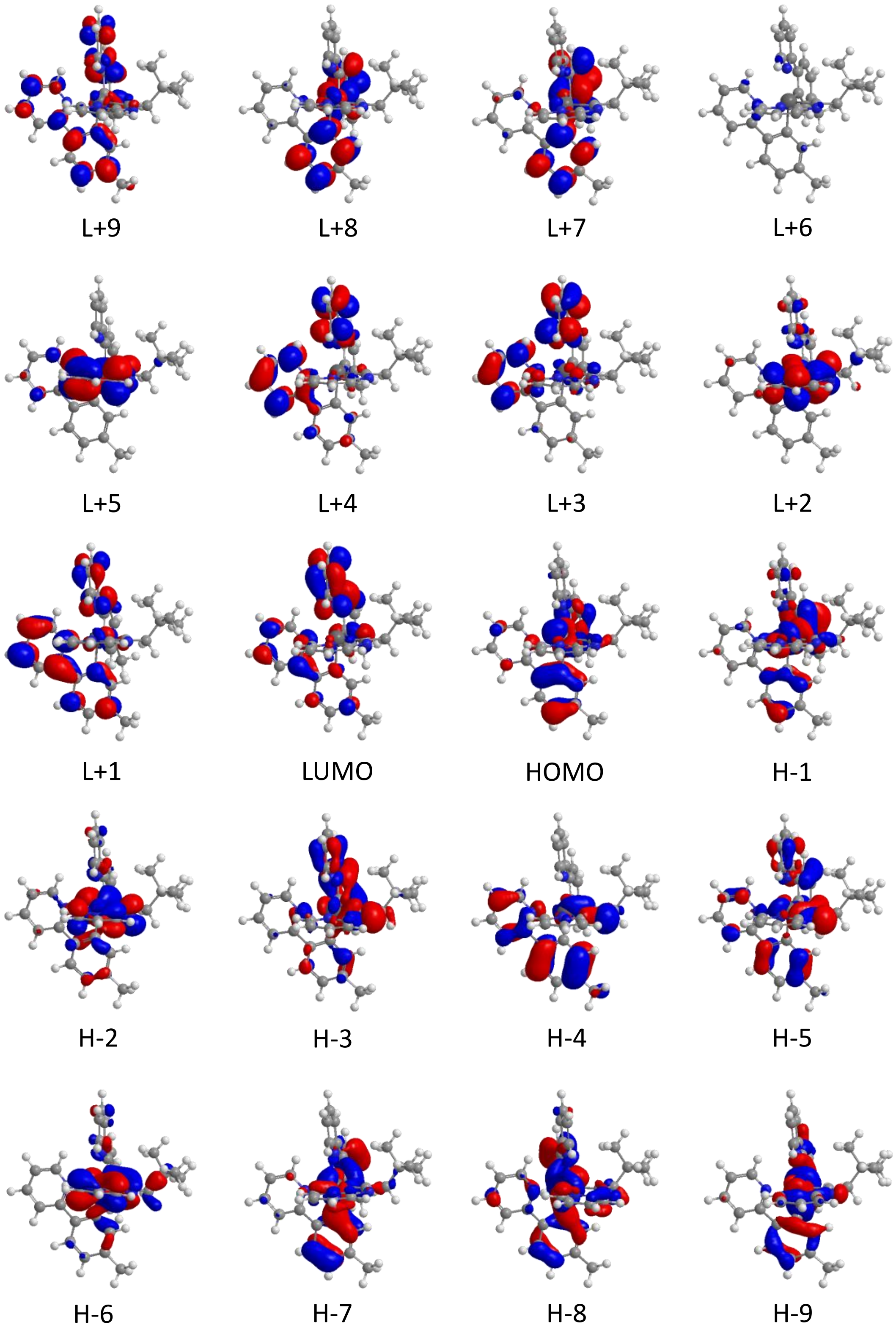

Figure S6. Frontier molecular orbitals of complex **13** (isovalue 0.003 au).

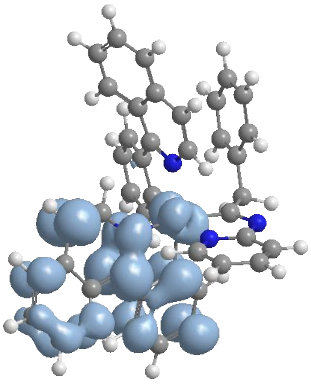

11

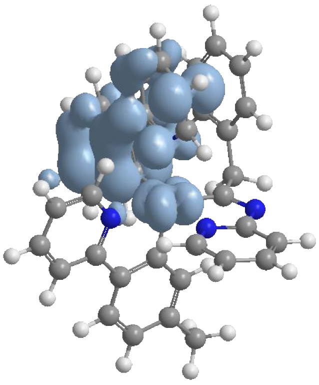

12

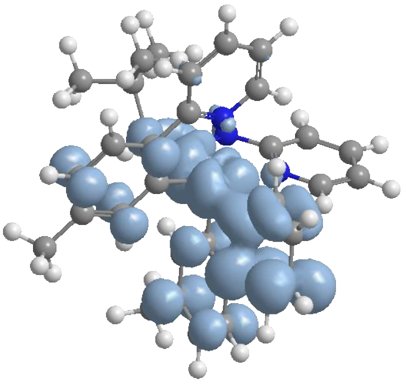

13

Figure S7. Spin density distributions for the optimized triplet T_1 of complexes **11-13** (0.002 isovalue).

Cyclic Voltammograms of Complexes 11-13

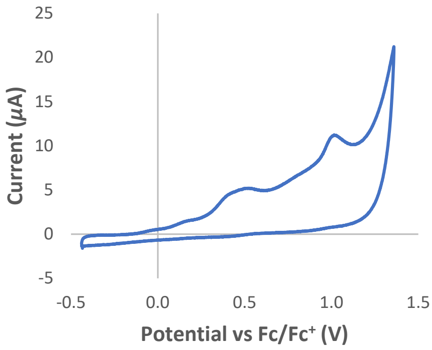

11

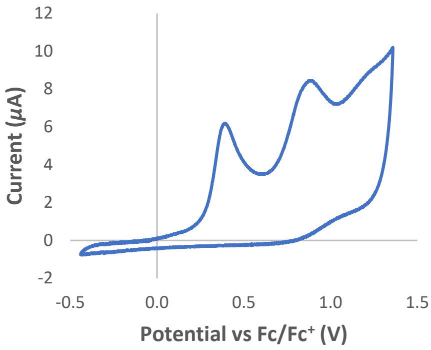

12

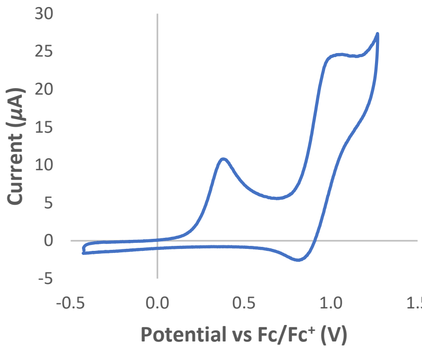

13

Figure S8. Cyclic voltammograms of complexes **11-13** in 10^{-3} M solutions with Bu_4NPF_6 as supporting electrolyte (0.1 M) at a scan rate of 250 mV s^{-1}. The potentials were referenced to the ferrocene/ferrocenium (Fc/Fc^+) couple.

Normalized Excitation and Emission Spectra of Complexes 11-13

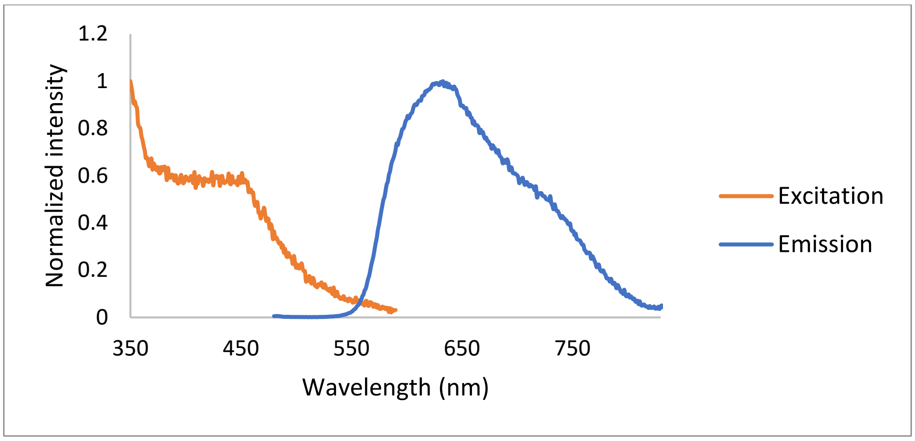

Figure S9. Normalized emission and excitation spectra of complex **11** in PMMA film (5 wt %) at 298 K. Excitation spectrum with emission at 630 nm, emission spectrum with excitation at 440 nm.

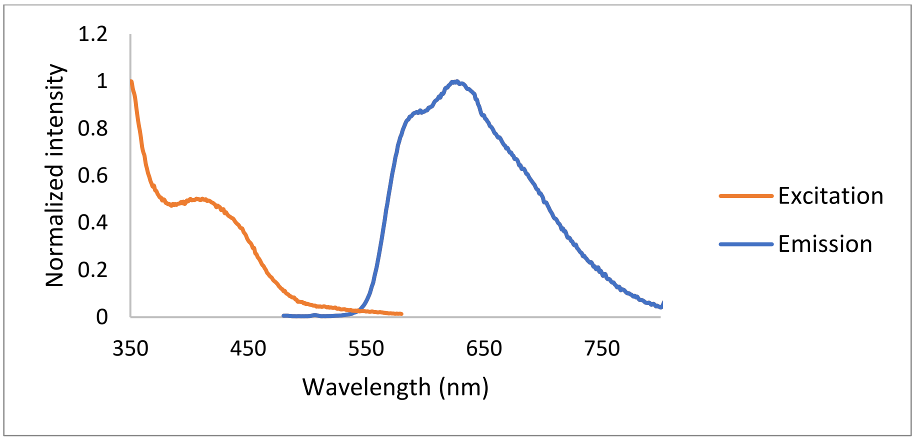

Figure S10. Normalized emission and excitation spectra for a 1 x 10^{-5} M solution of complex **11** in 2-MeTHF at 298 K. Excitation spectrum with emission at 620 nm, emission spectrum with excitation at 440 nm.

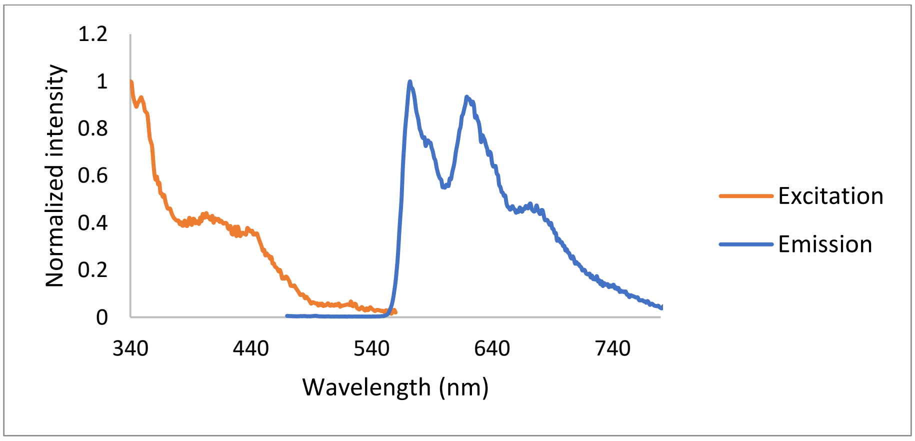

Figure S11. Normalized emission and excitation spectra for a 1 x 10^{-5} M solution of complex **11** in 2-MeTHF at 77 K. Excitation spectrum with emission at 600 nm, emission spectrum with excitation at 430 nm.

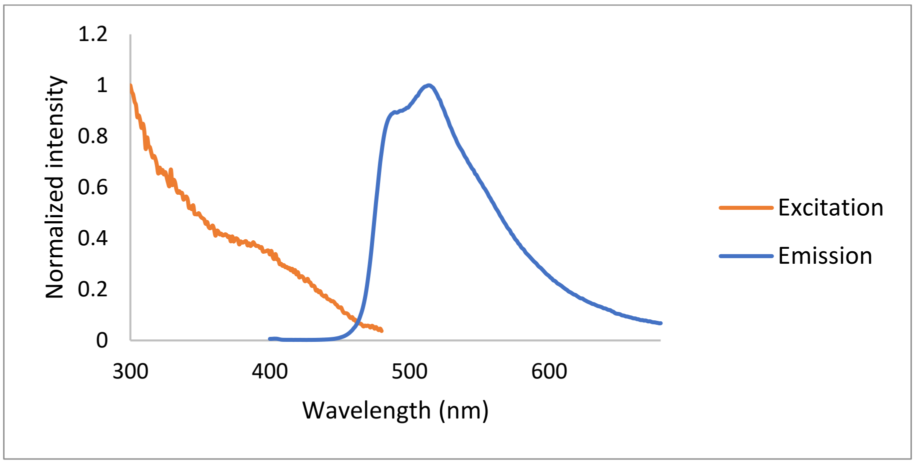

Figure S12. Normalized emission and excitation spectra of complex **12** in PMMA film (5 wt %) at 298 K. Excitation spectrum with emission at 520 nm, emission spectrum with excitation at 360 nm.

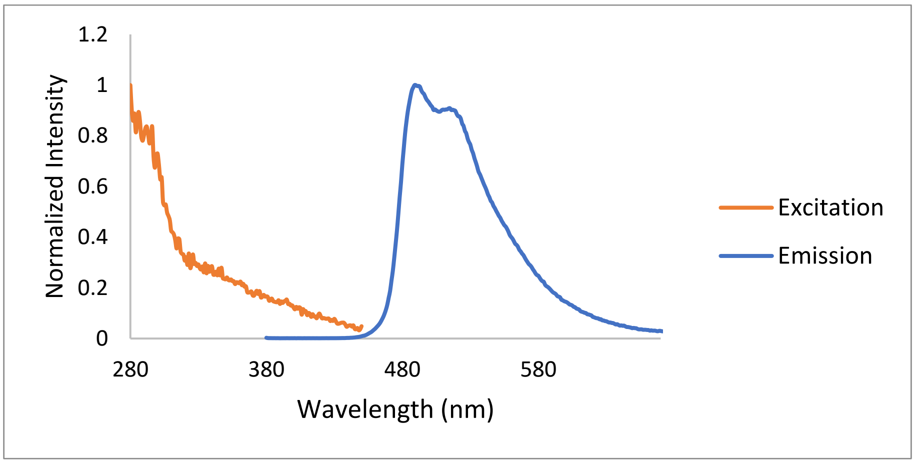

Figure S13. Normalized emission and excitation spectra for a 1 x 10^{-5} M solution of complex **12** in 2-MeTHF at 298 K. Excitation spectrum with emission at 500 nm, emission spectrum with excitation at 340 nm.

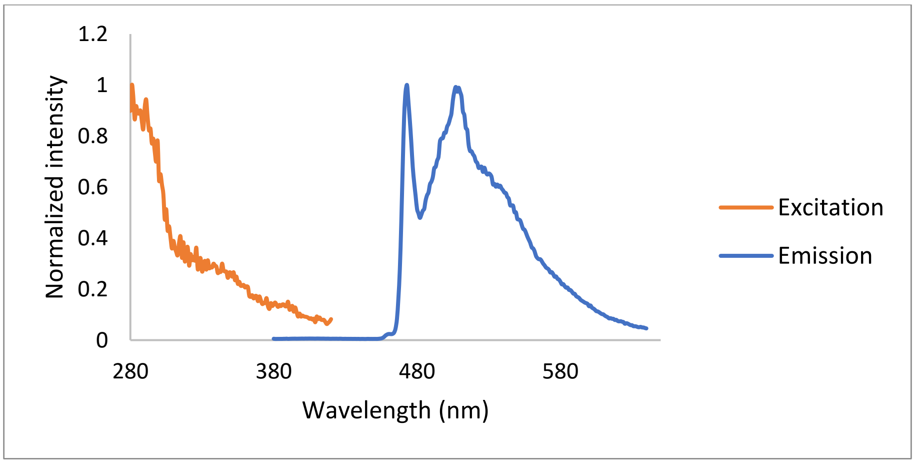

Figure S14. Normalized emission and excitation spectra for a 1 x 10^{-5} M solution of complex **12** in 2-MeTHF at 77 K. Excitation spectrum with emission at 480 nm, emission spectrum with excitation at 340 nm.

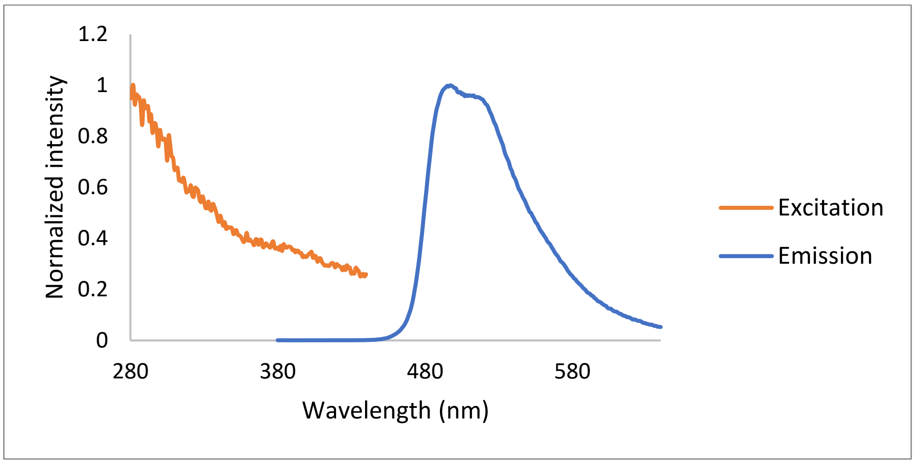

Figure S15. Normalized emission and excitation spectra of complex **13** in PMMA film (5 wt %) at 298 K. Excitation spectrum with emission at 480 nm, emission spectrum with excitation at 340 nm.

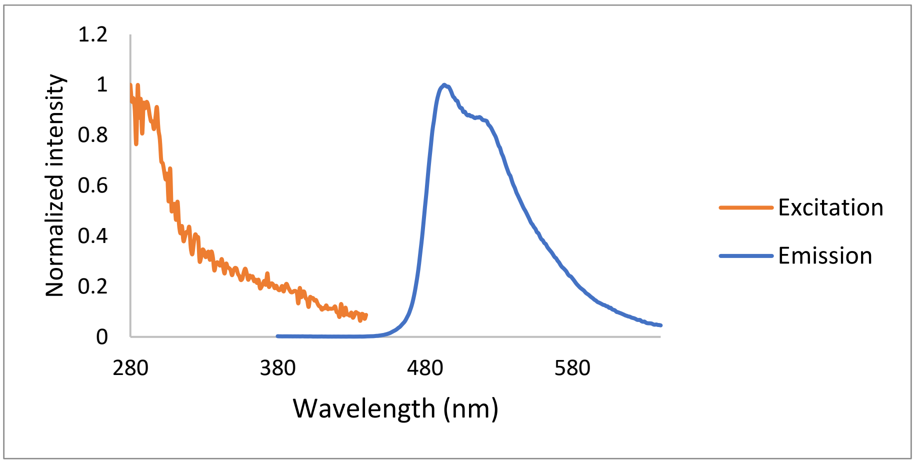

Figure S16. Normalized emission and excitation spectra for a 1 x 10^{-5} M solution of complex **13** in 2-MeTHF at 298 K. Excitation spectrum with emission at 480 nm, emission spectrum with excitation at 340 nm.

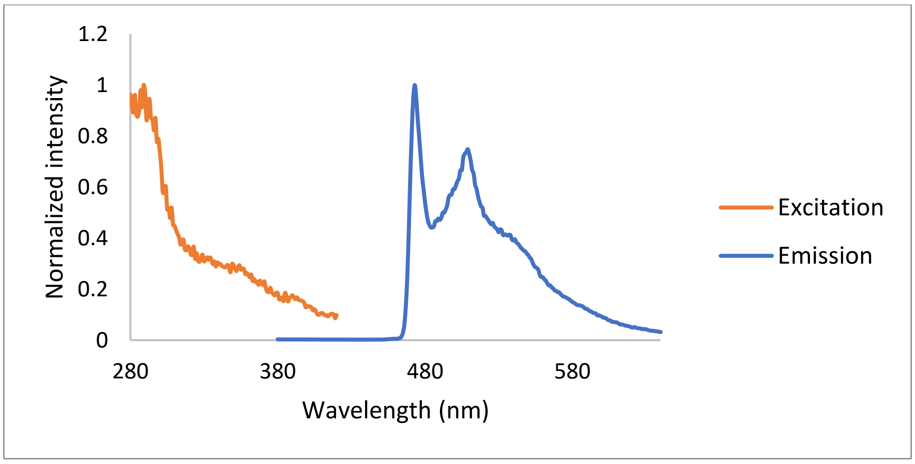

Figure S17. Normalized emission and excitation spectra for a 1 x 10^{-5} M solution of complex **13** in 2-MeTHF at 77 K. Excitation spectrum with emission at 480 nm, emission spectrum with excitation at 340 nm.

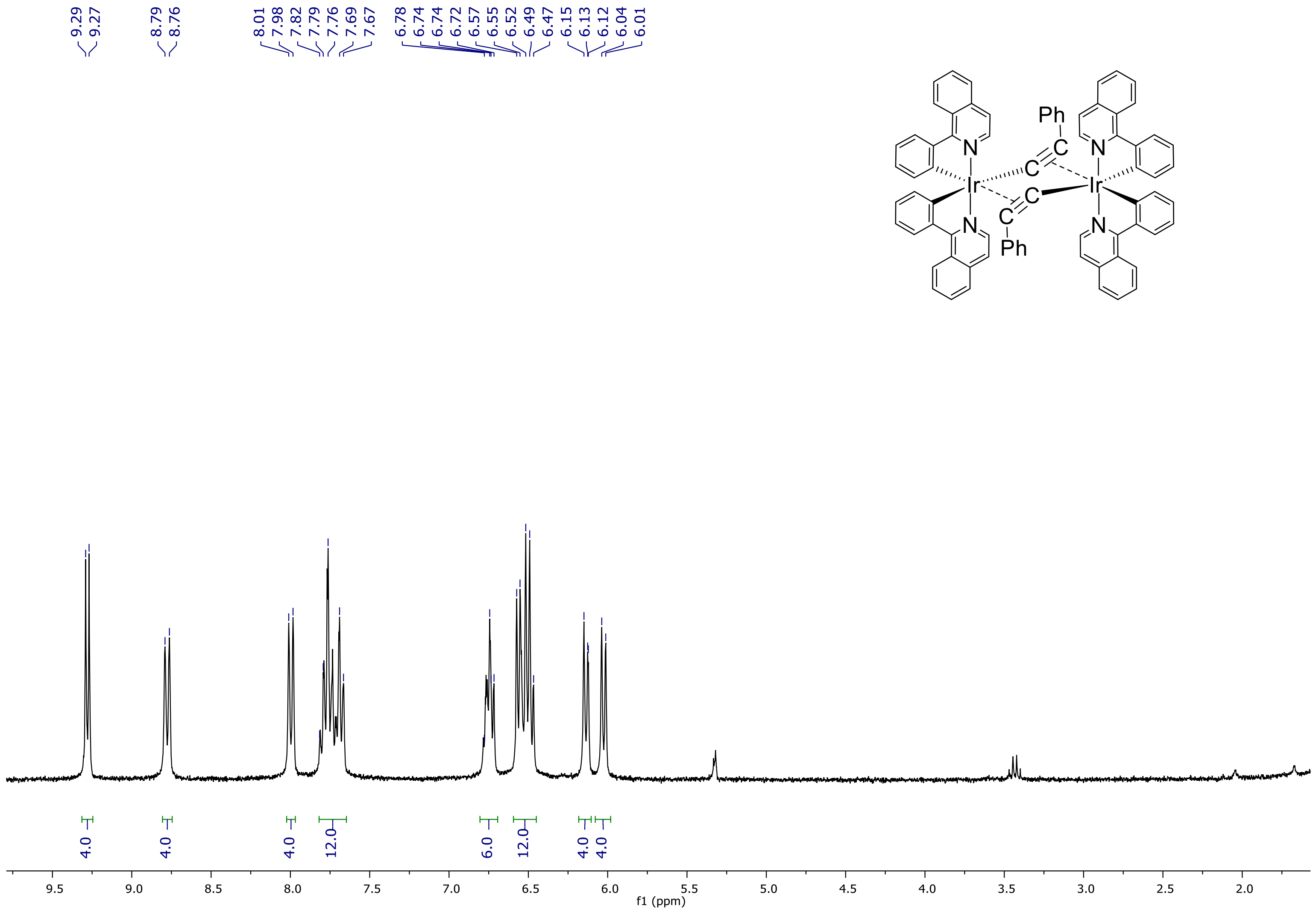

Figure S18. ^{1}H NMR spectrum (300 MHz, CD_2Cl_3, 298 K) of complex **3.**

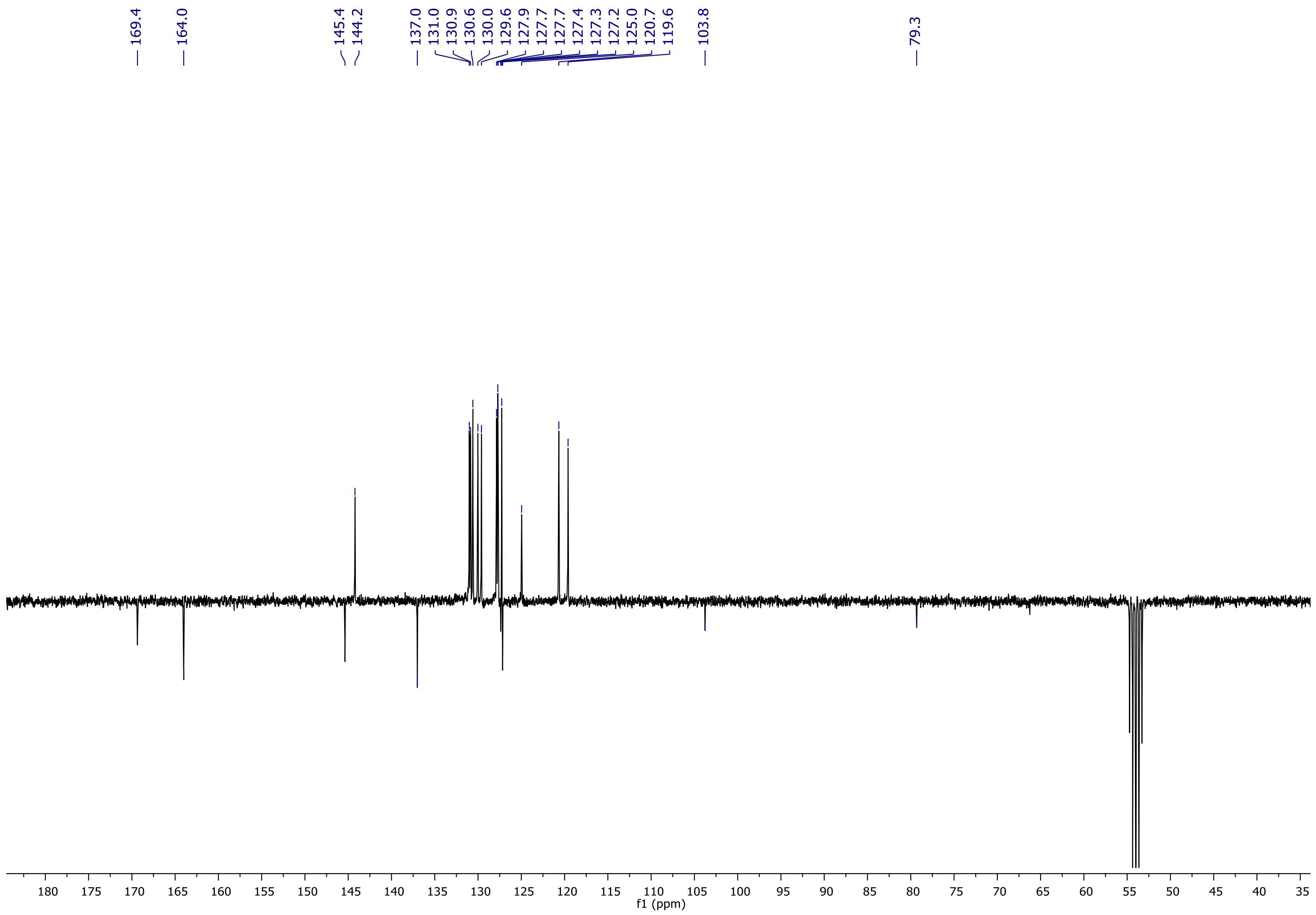

Figure S19. $^{13}C\{^1H\}$-APT NMR spectrum (75 MHz, CD_2Cl_2, 298 K) of complex **3.**

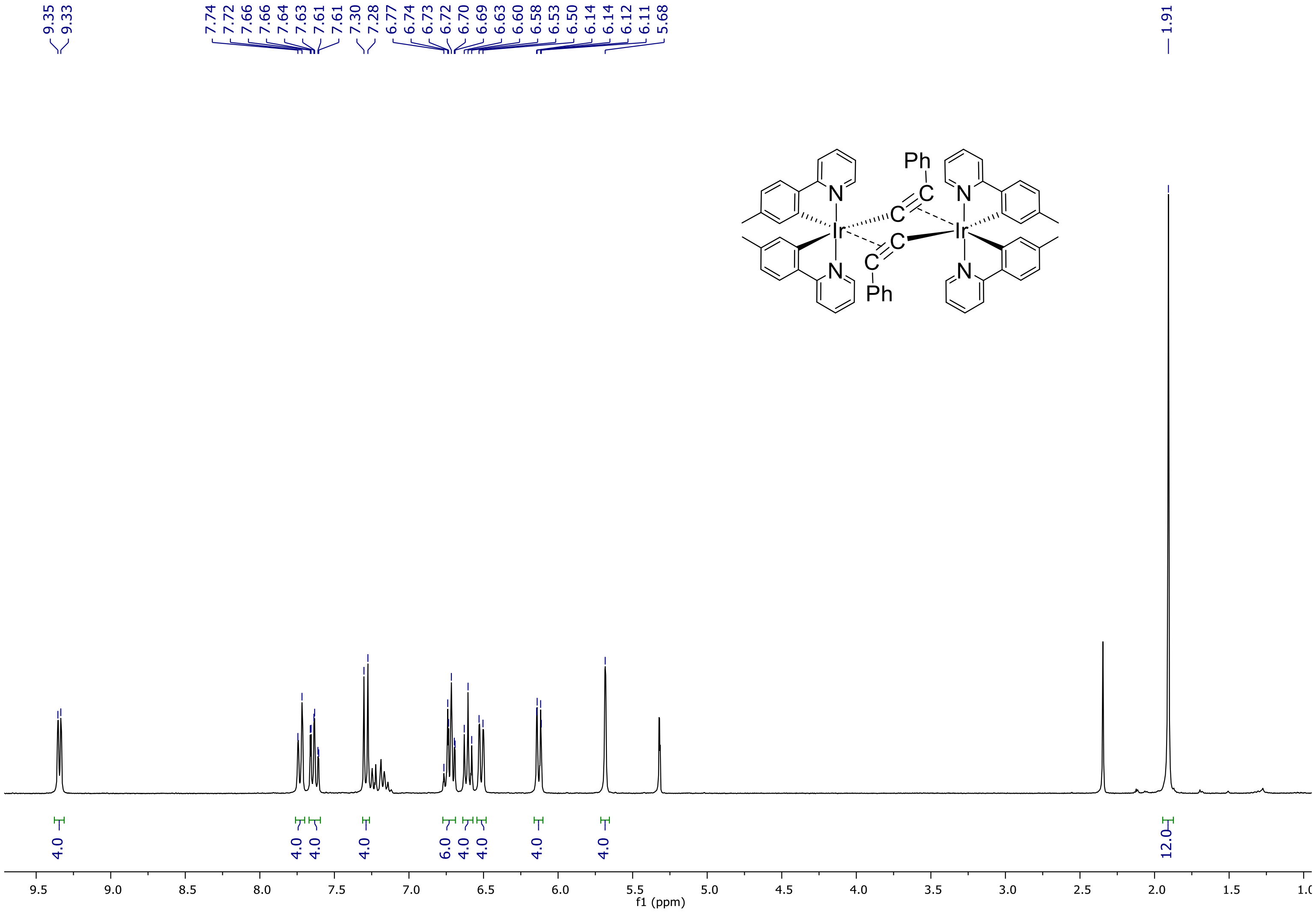

Figure S20. ^{1}H NMR spectrum (300 MHz, CD_2Cl_2, 298 K) of complex **4.**

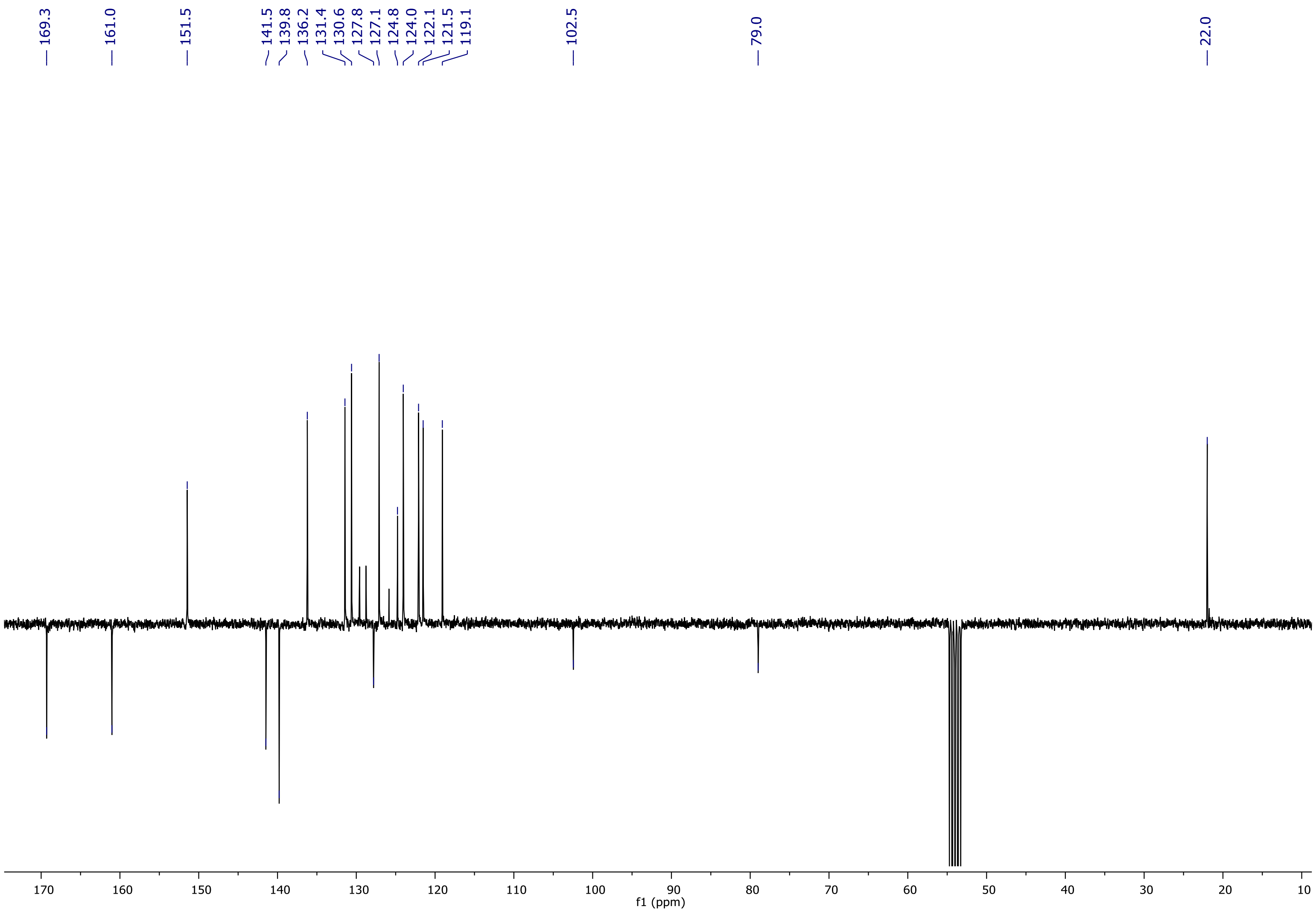

Figure S21. $^{13}C\{^{1}H\}$-APT NMR spectrum (75 MHz, CD_2Cl_2, 298 K) of complex **4.**

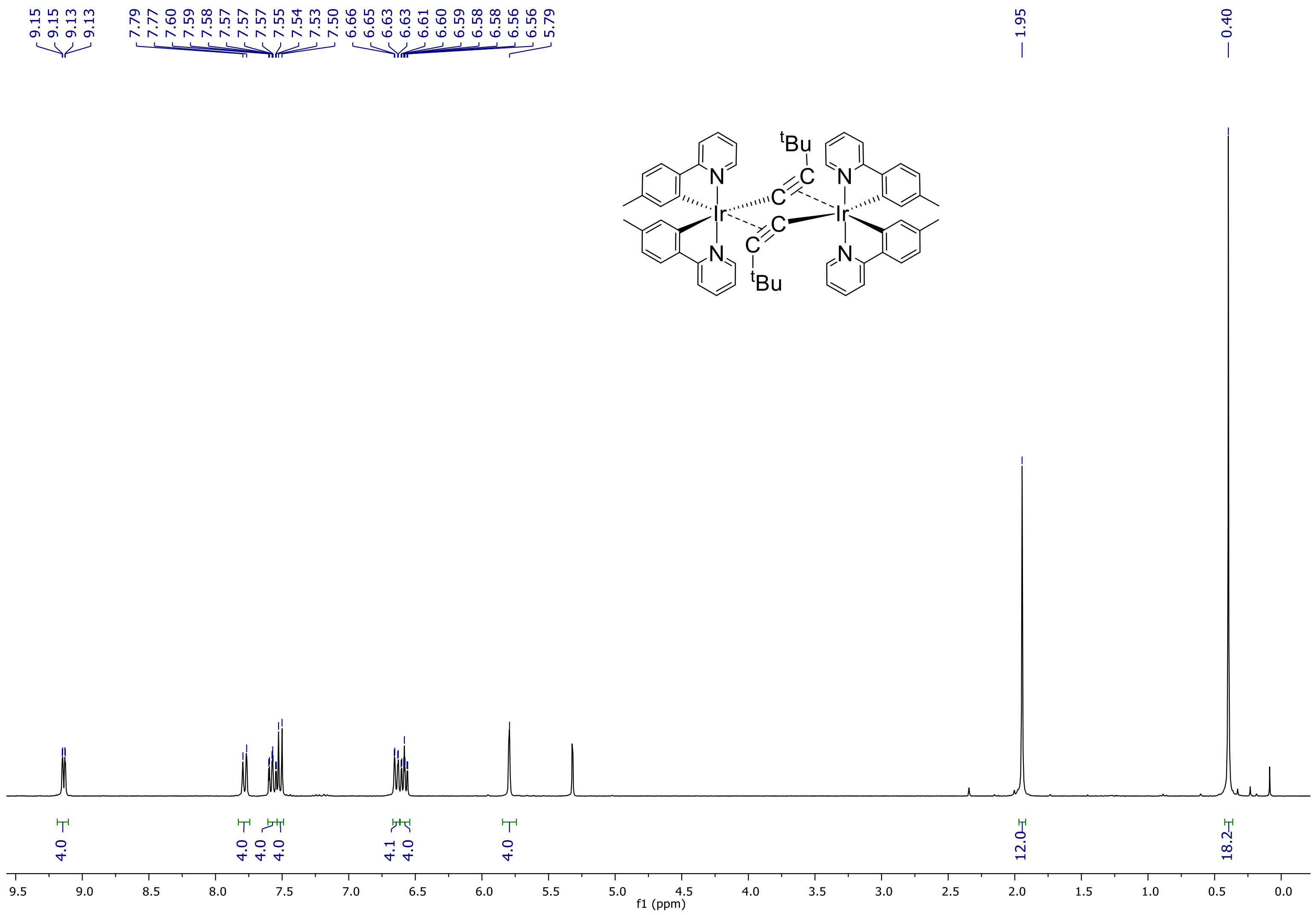

Figure S22. ^{1}H NMR spectrum (300 MHz, CD_2Cl_2, 298 K) of complex **5.**

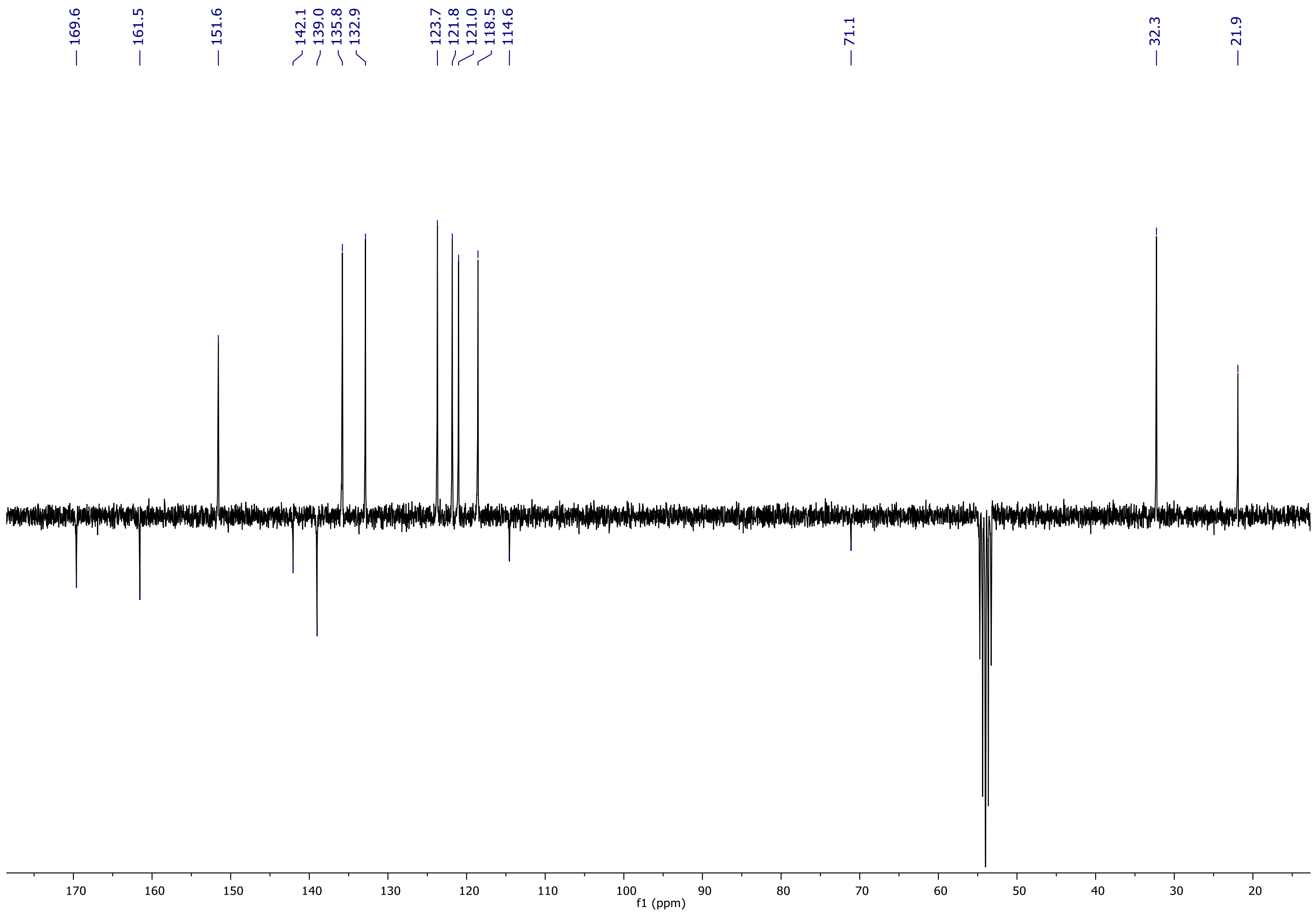

Figure S23. $^{13}C\{^{1}H\}$-APT NMR spectrum (75 MHz, CD_2Cl_2, 298 K) of complex **5.**

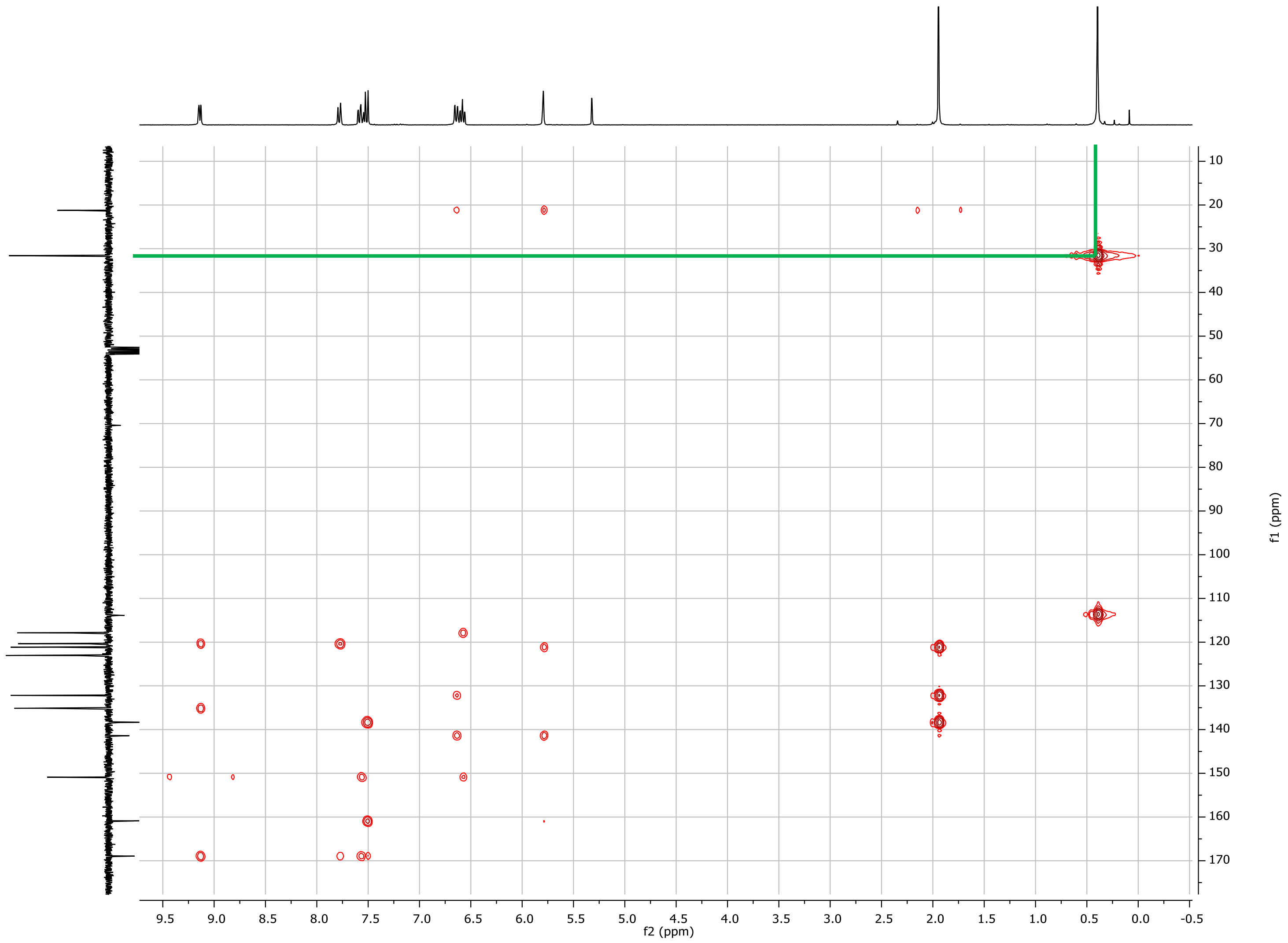

Figure S24. HMBC ^{1}H-$^{13}C\{^{1}H\}$ NMR spectrum (300-75 MHz, CD_2Cl_2, 298 K) of compound **5.**

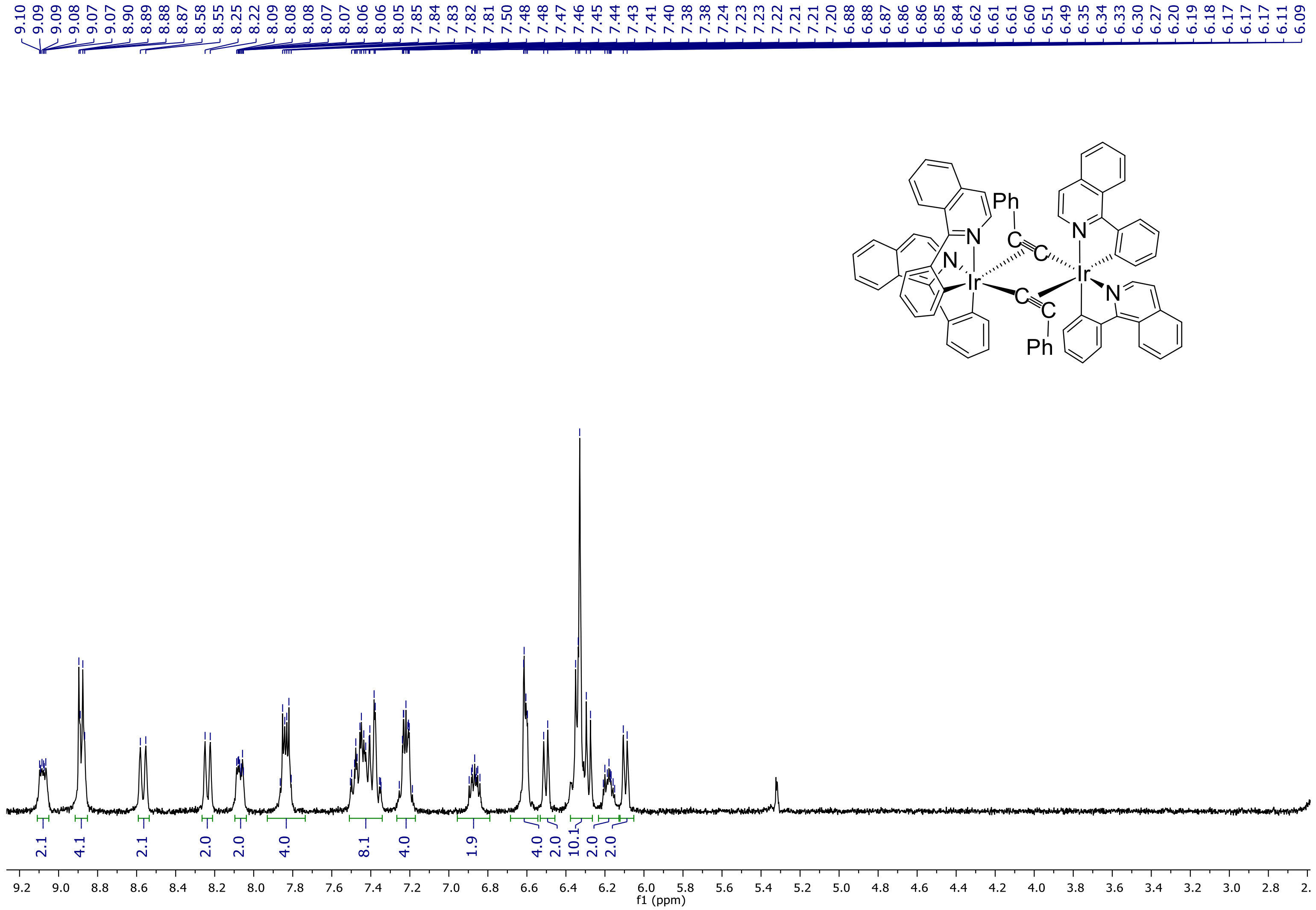

Figure S25. ^{1}H NMR spectrum (300 MHz, CD_2Cl_2, 298 K) of complex **6.**

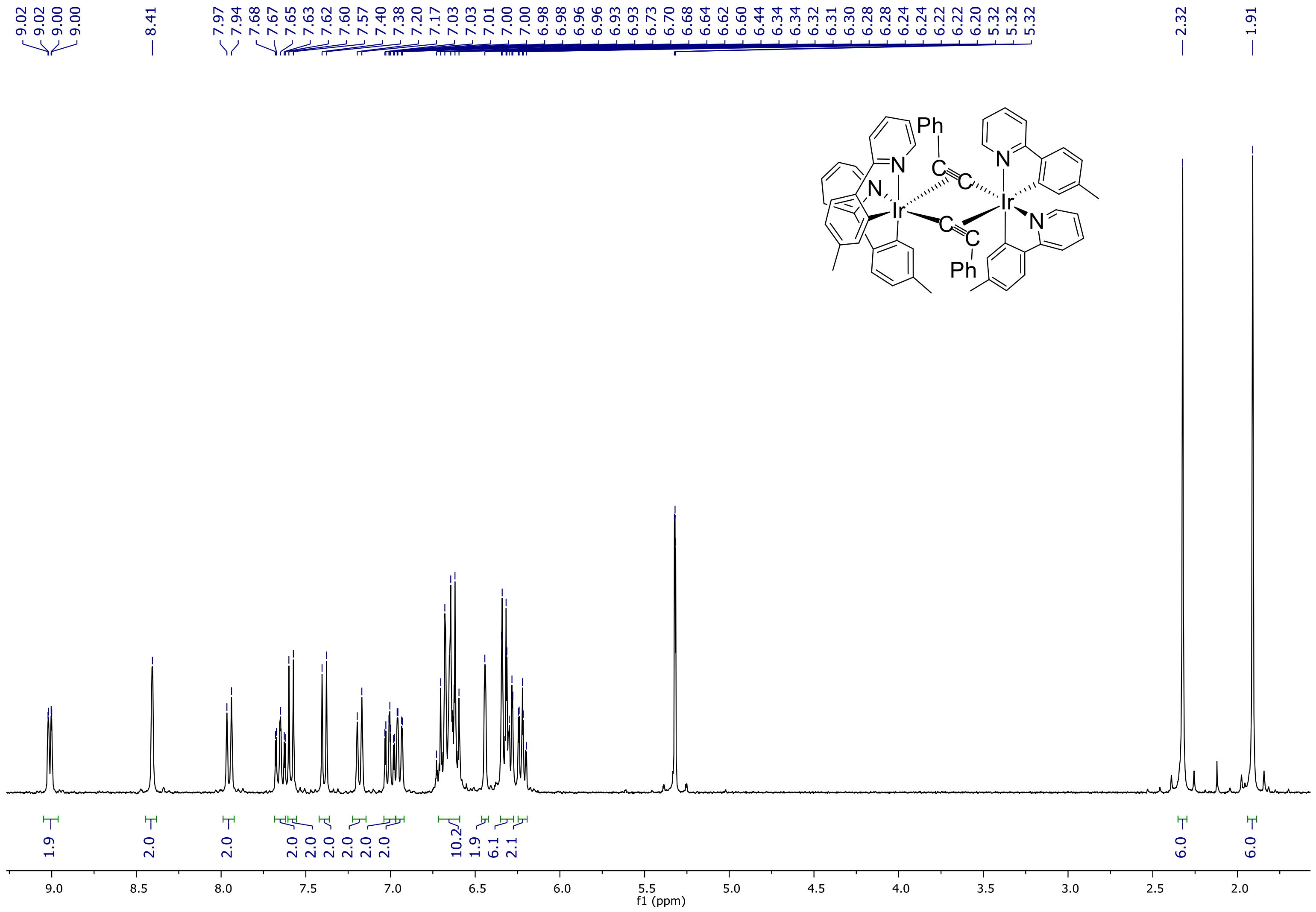

Figure S26. ^{1}H NMR spectrum (300 MHz, CD_2Cl_2, 298 K) of complex **7.**

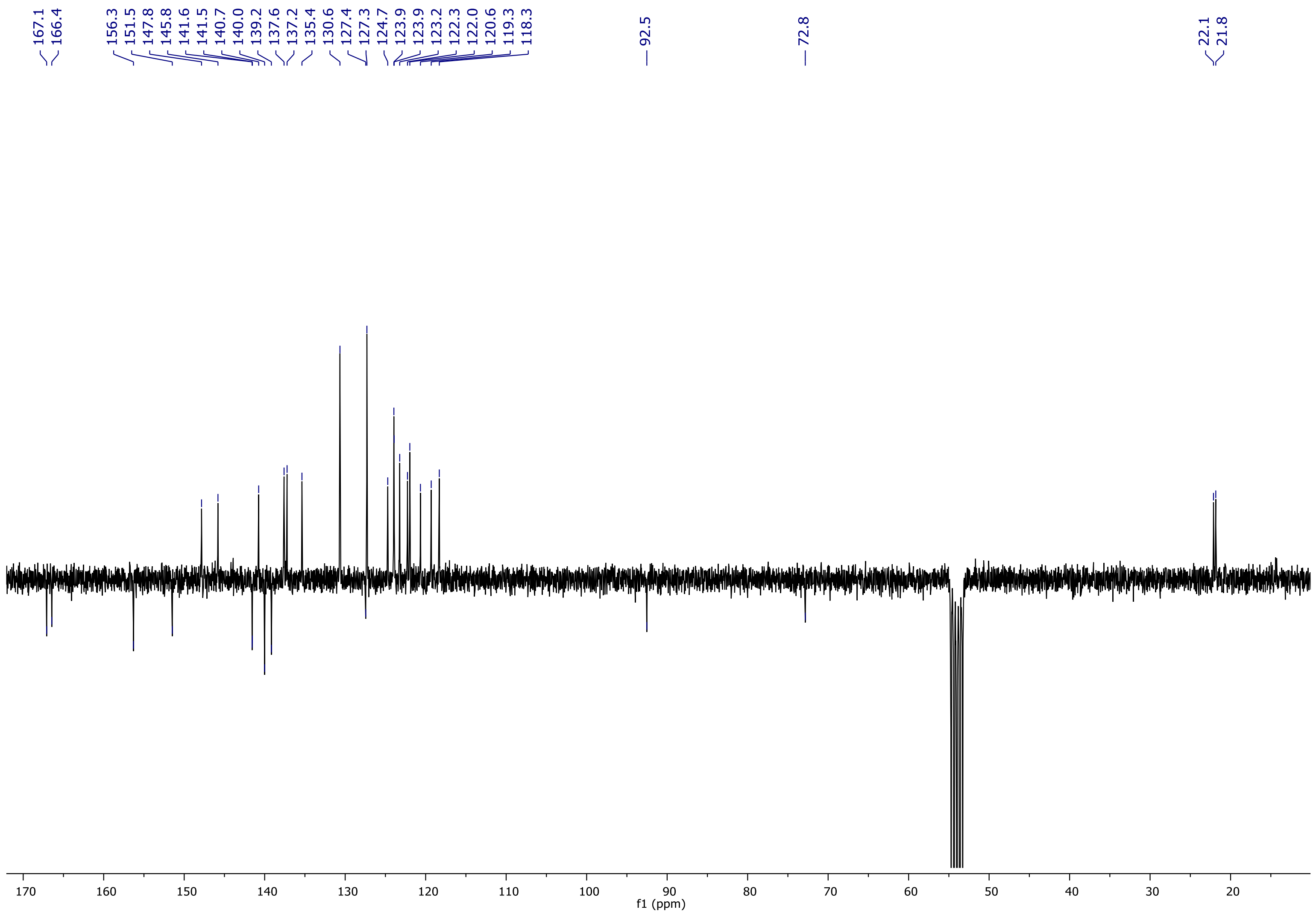

Figure S27. $^{13}C\{^1H\}$-APT NMR spectrum (75 MHz, CD_2Cl_2, 298 K) of complex **7.**

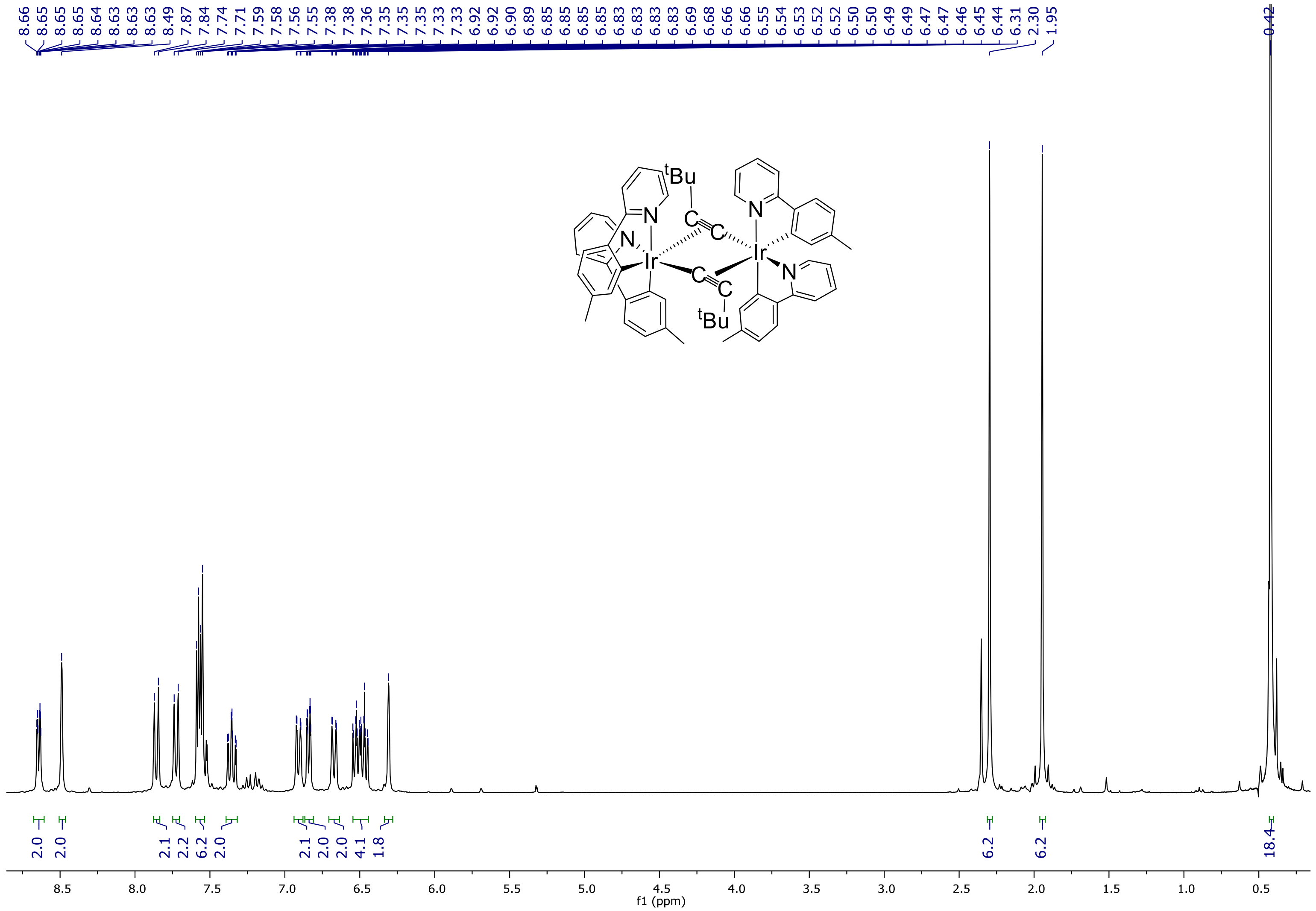

Figure S28. ^{1}H NMR spectrum (300 MHz, CD_2Cl_2, 298 K) of complex **8.**

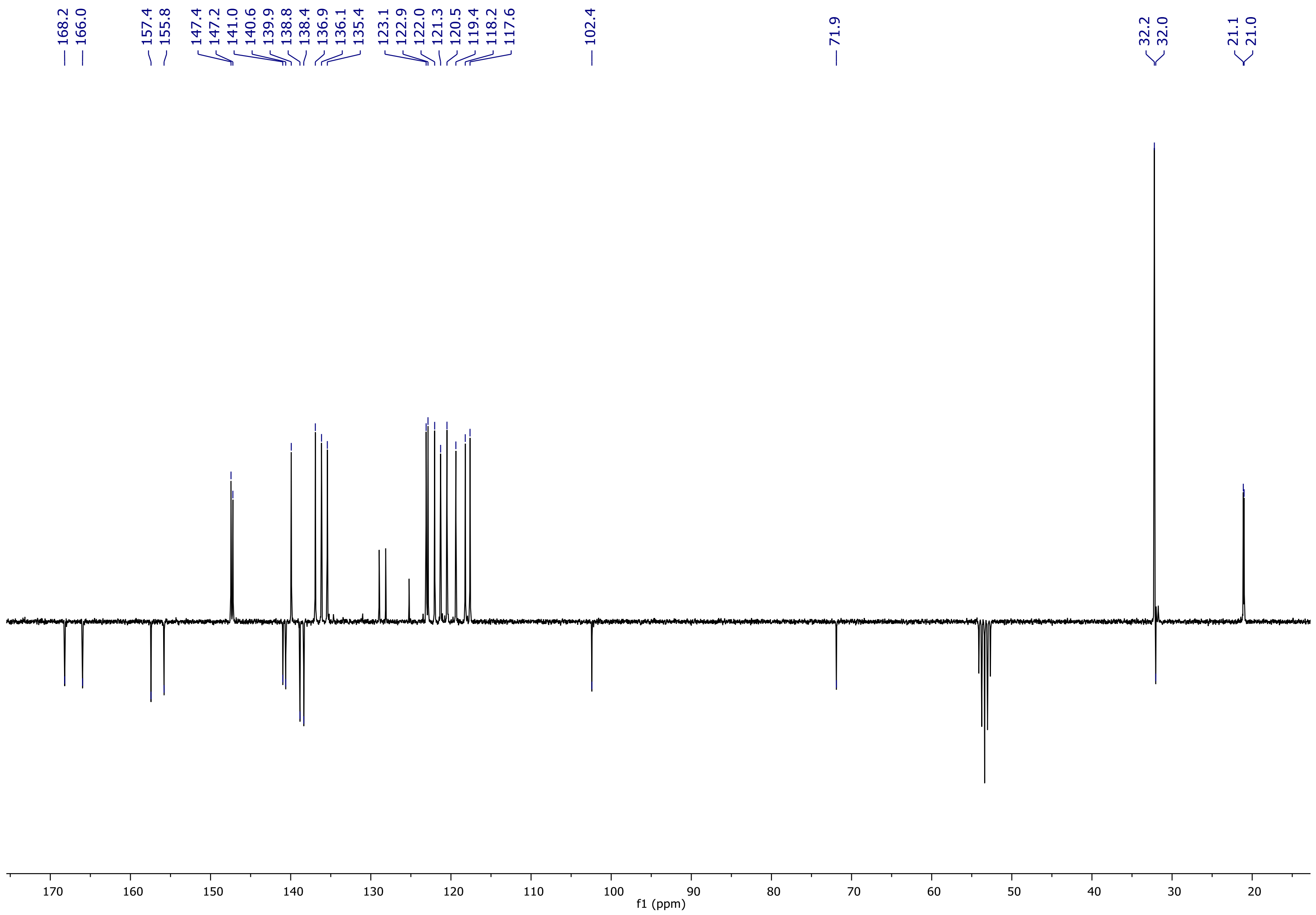

Figure S29. $^{13}C\{^{1}H\}$-APT NMR spectrum (75 MHz, CD_2Cl_2, 298 K) of complex **8.**

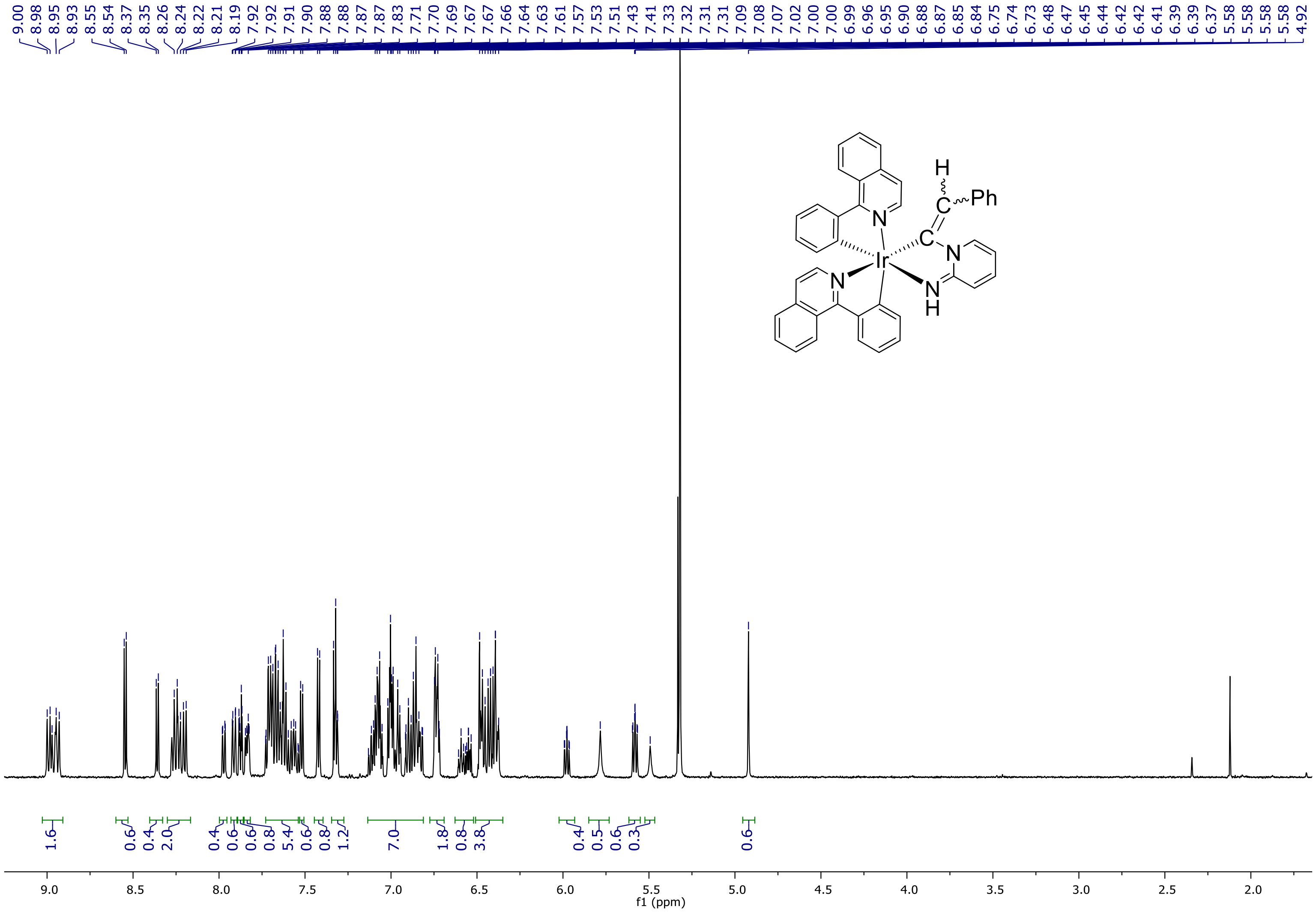

Figure S30. ^{1}H NMR spectrum (500 MHz, CD_2Cl_2, 298 K) of complex **9.**

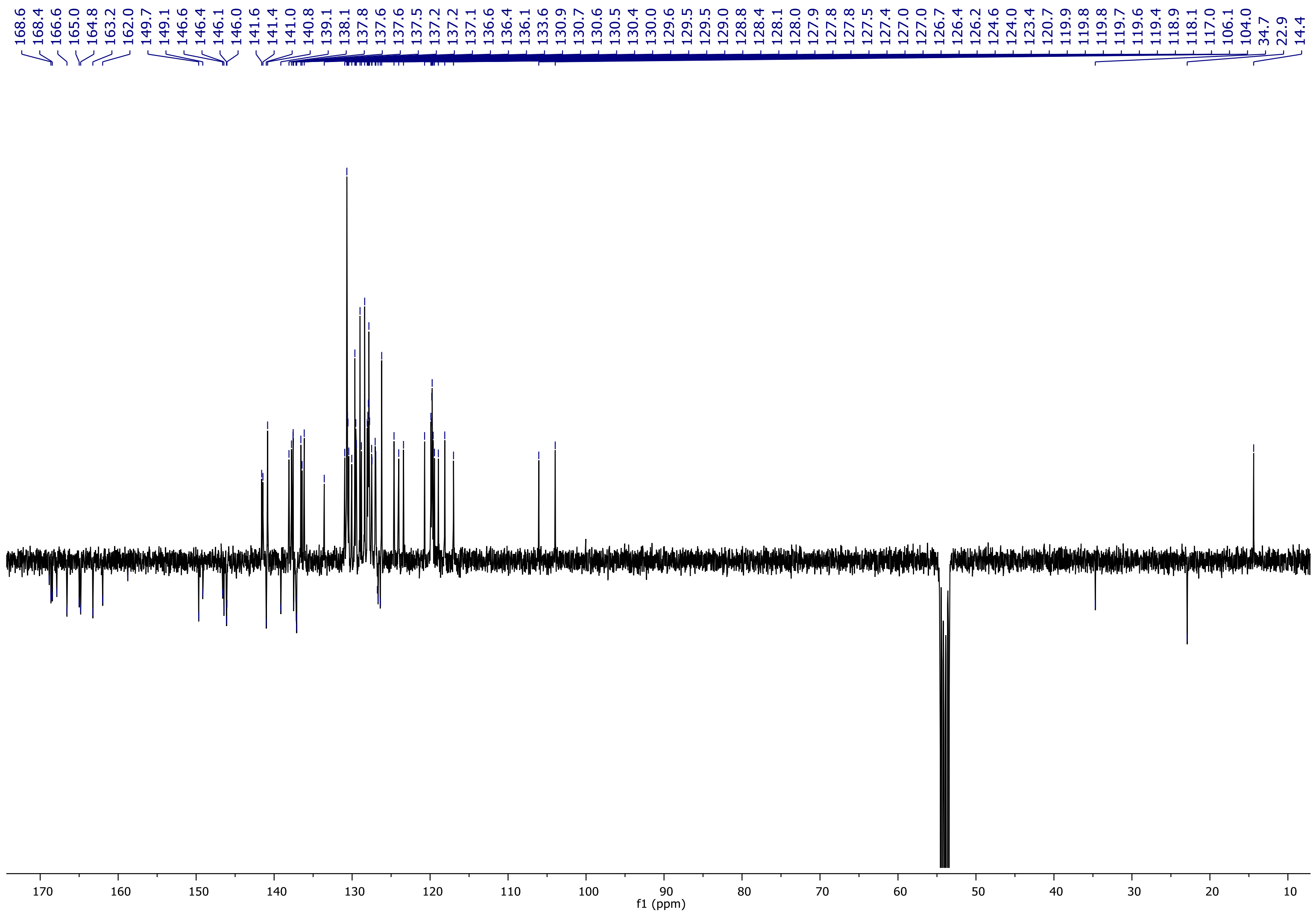

Figure S31. $^{13}C\{^{1}H\}$-APT NMR spectrum (100 MHz, CD_2Cl_2, 298 K) of complex **9.**

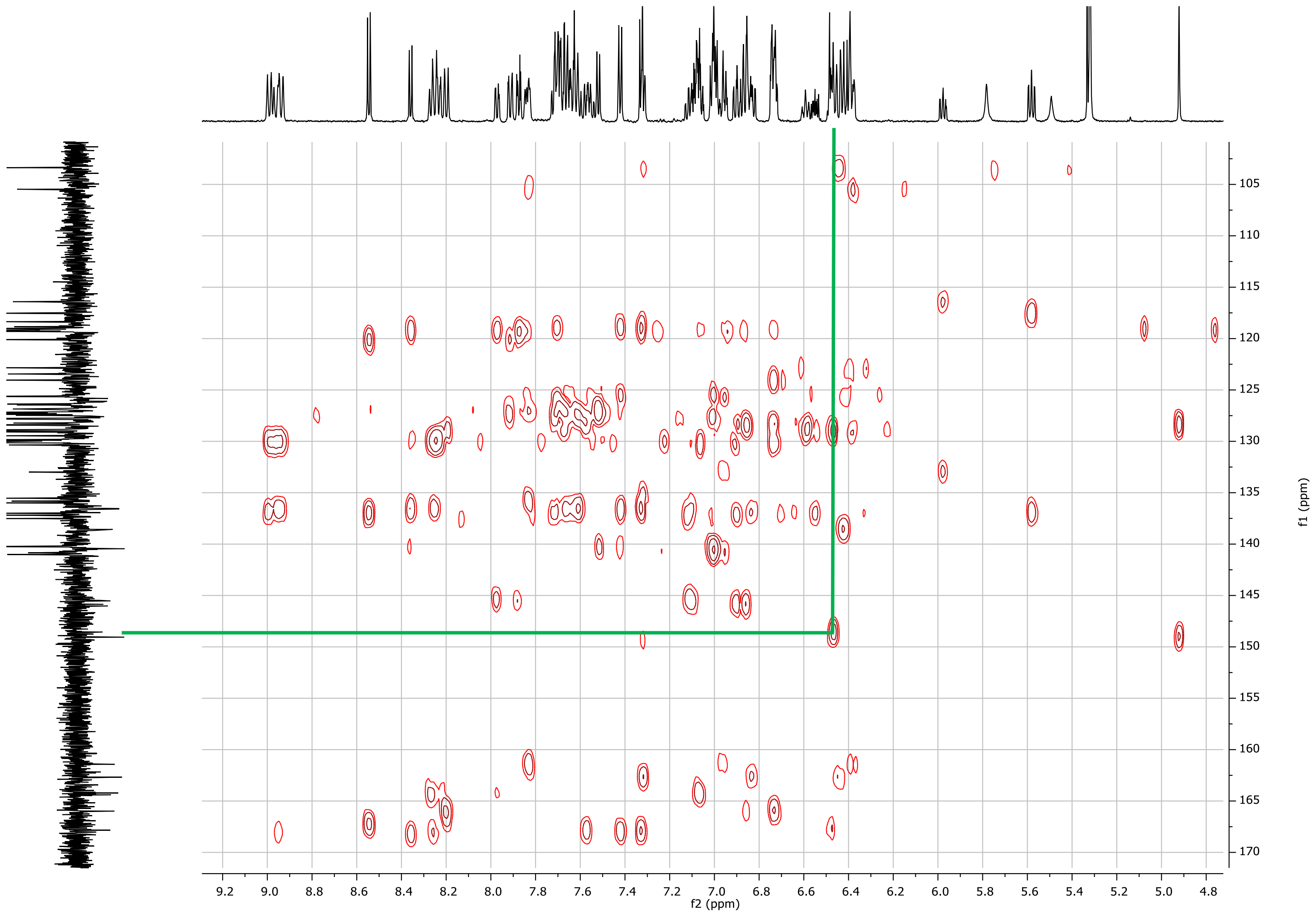

Figure S32. HMBC ^{1}H-$^{13}C\{^{1}H\}$ NMR spectrum (125 MHz, CD_2Cl_2, 298 K) of complex **9.**

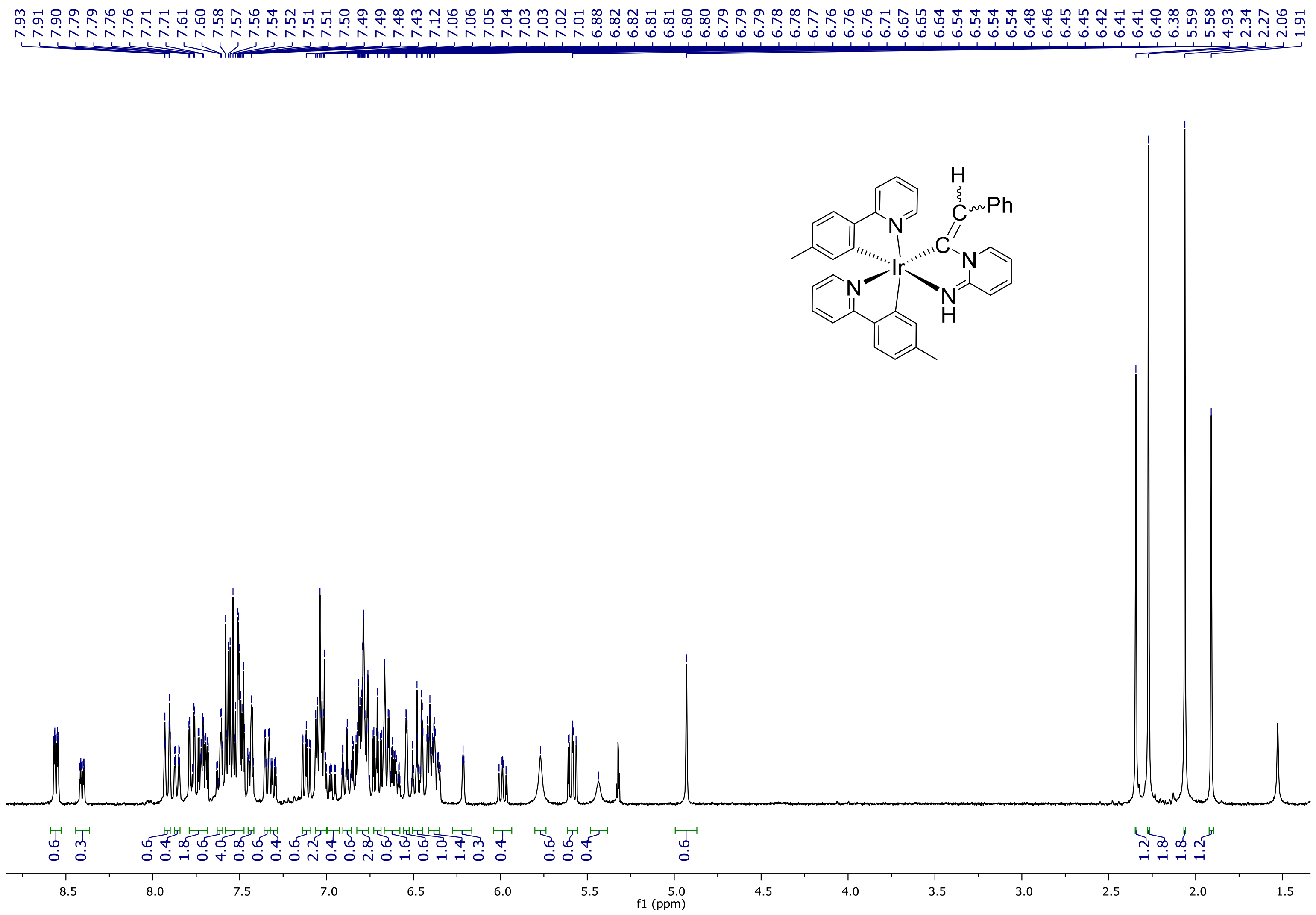

Figure S33. ^{1}H NMR spectrum (300 MHz, CD_2Cl_2, 298 K) of complex **10.**

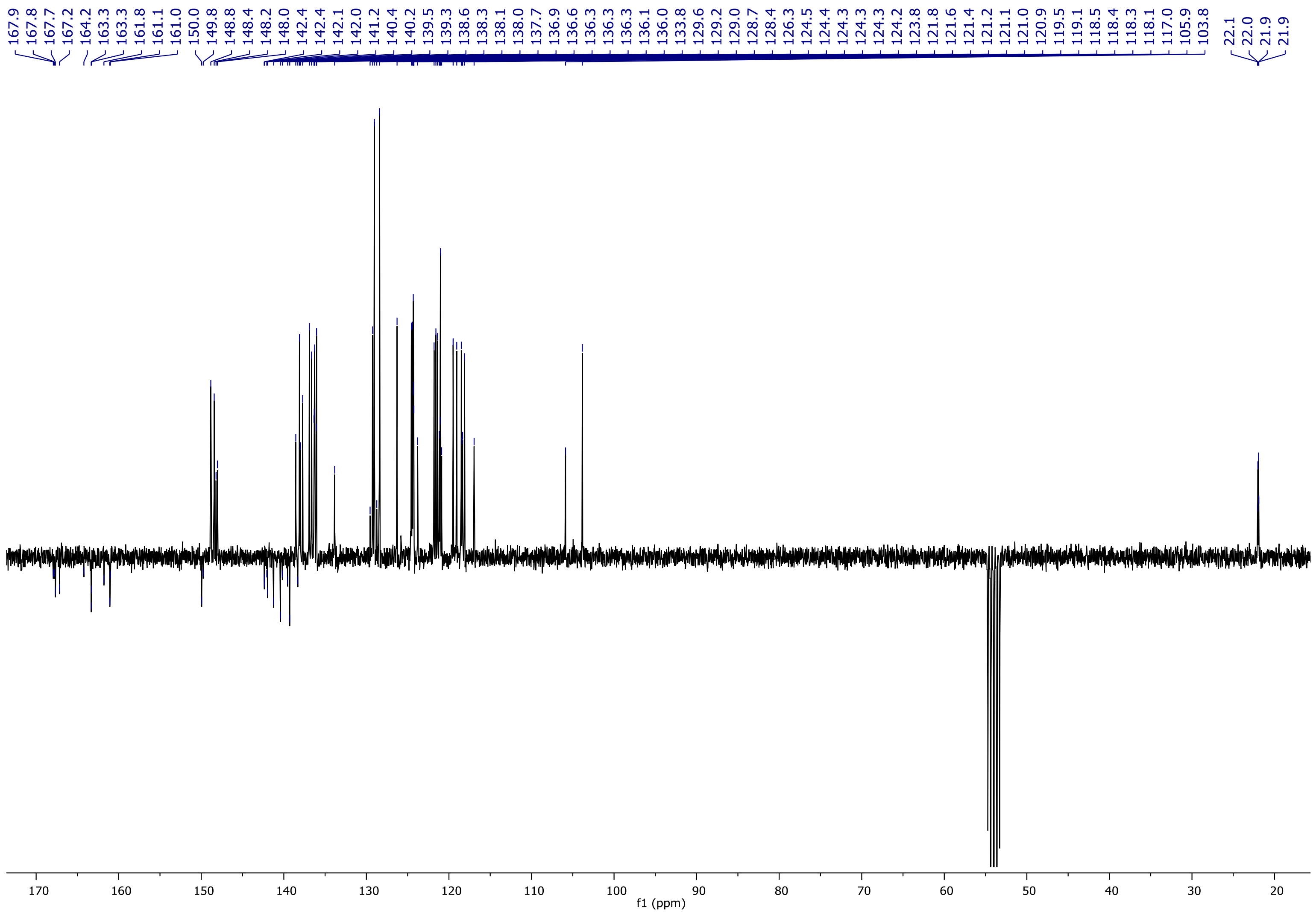

Figure S34. $^{13}C\{^{1}H\}$-APT NMR spectrum (75 MHz, CD_2Cl_2, 298 K) of complex **10.**

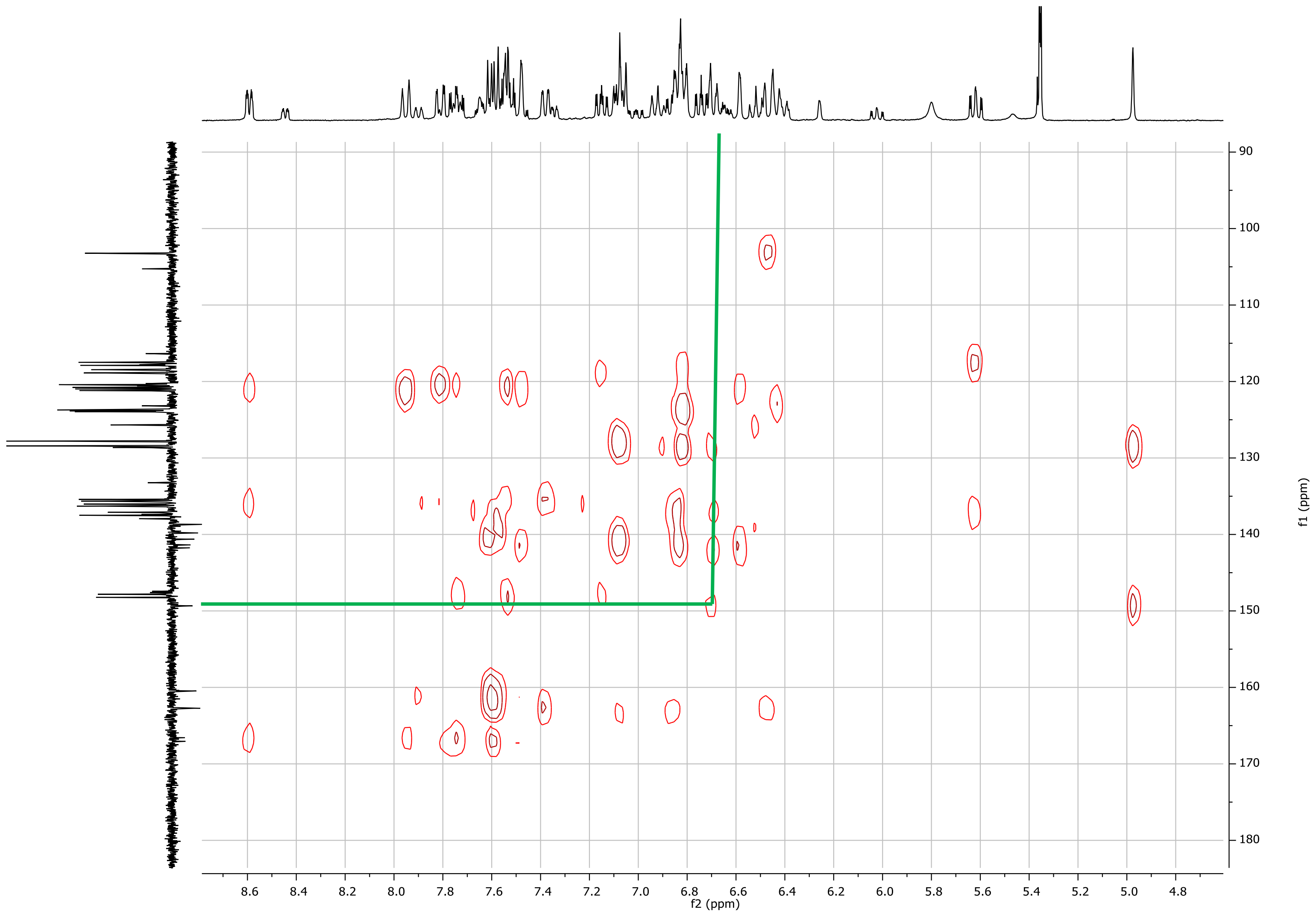

Figure S35. HMBC ^{1}H-$^{13}C\{^{1}H\}$ NMR spectrum (75 MHz, CD_2Cl_2, 298 K) of complex **10.**

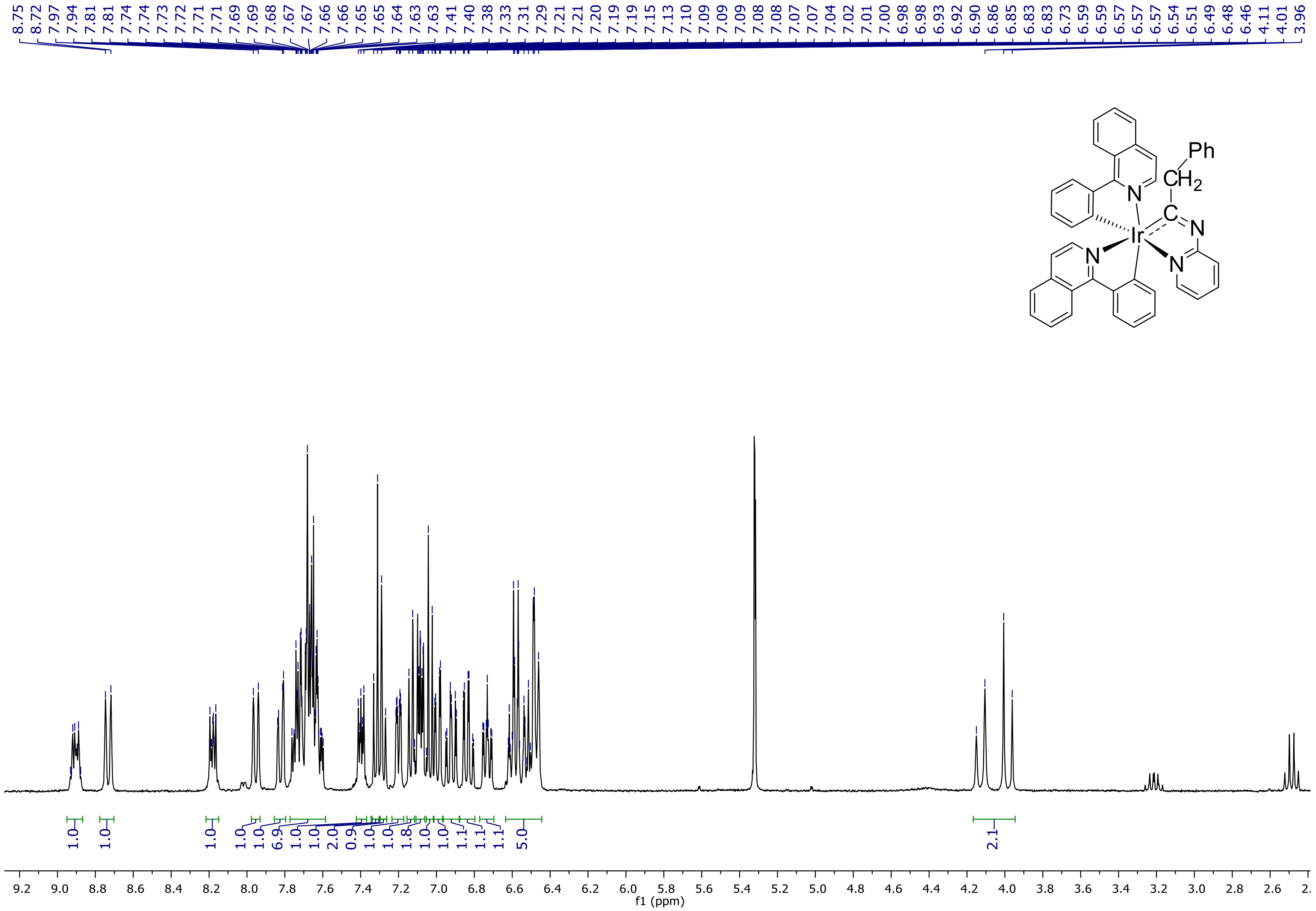

Figure S36. ^{1}H NMR spectrum (300 MHz, CD_2Cl_2, 298 K) of complex **11.**

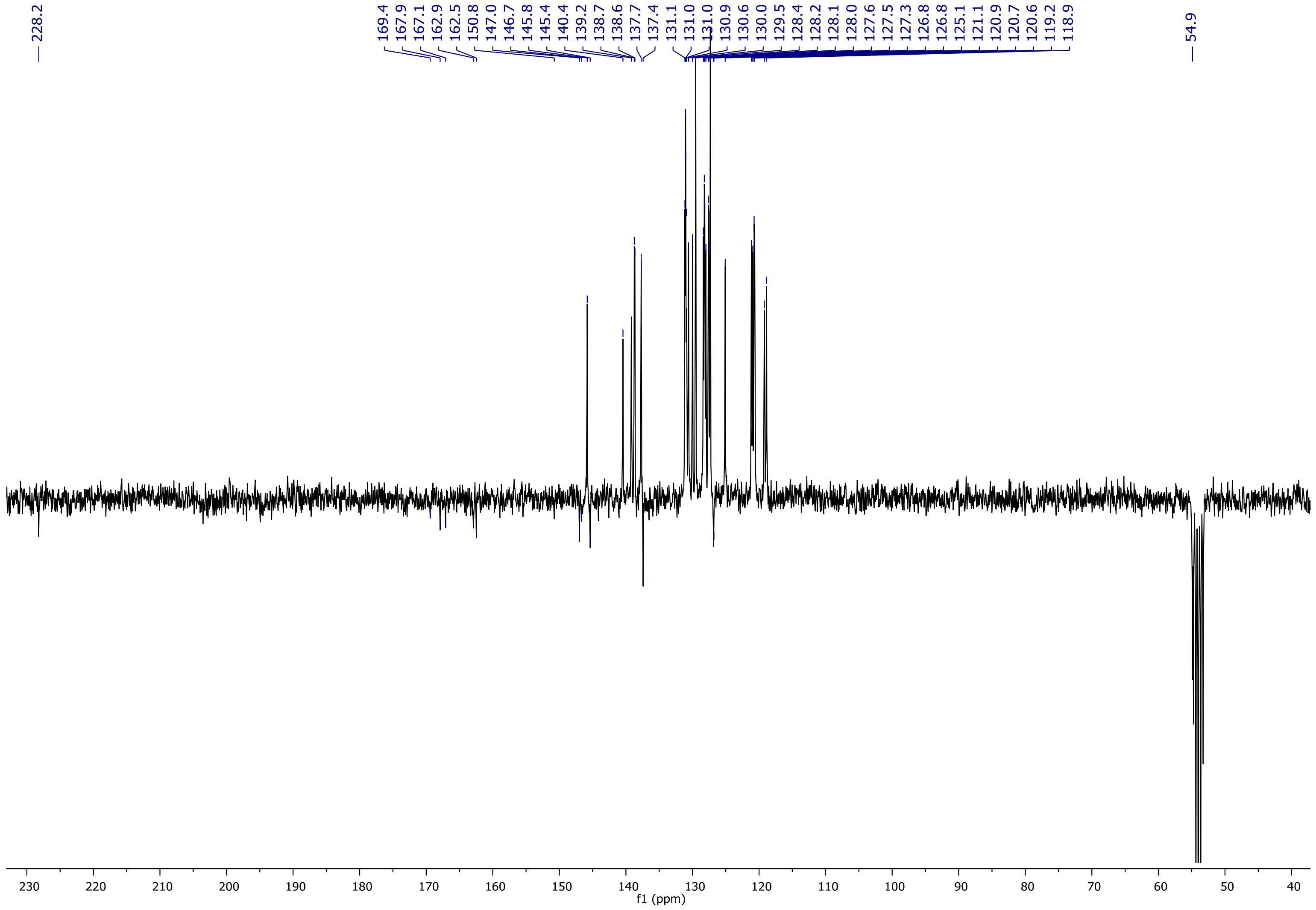

Figure S37. $^{13}C\{^1H\}$-APT NMR spectrum (75 MHz, CD_2Cl_2, 298 K) of complex **11.**

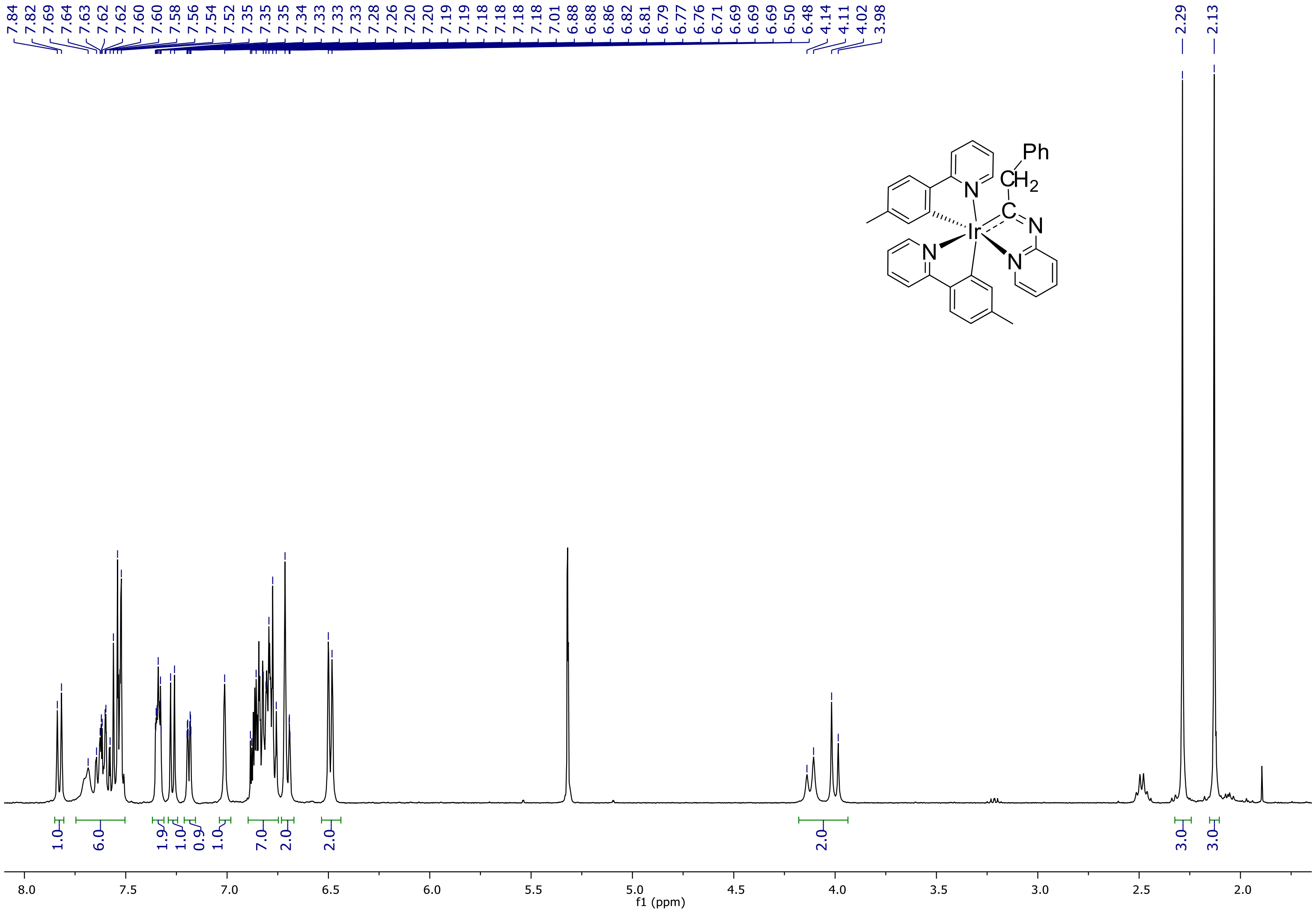

Figure S38. ^{1}H NMR spectrum (400 MHz, CD_2Cl_2, 298 K) of complex **12.**

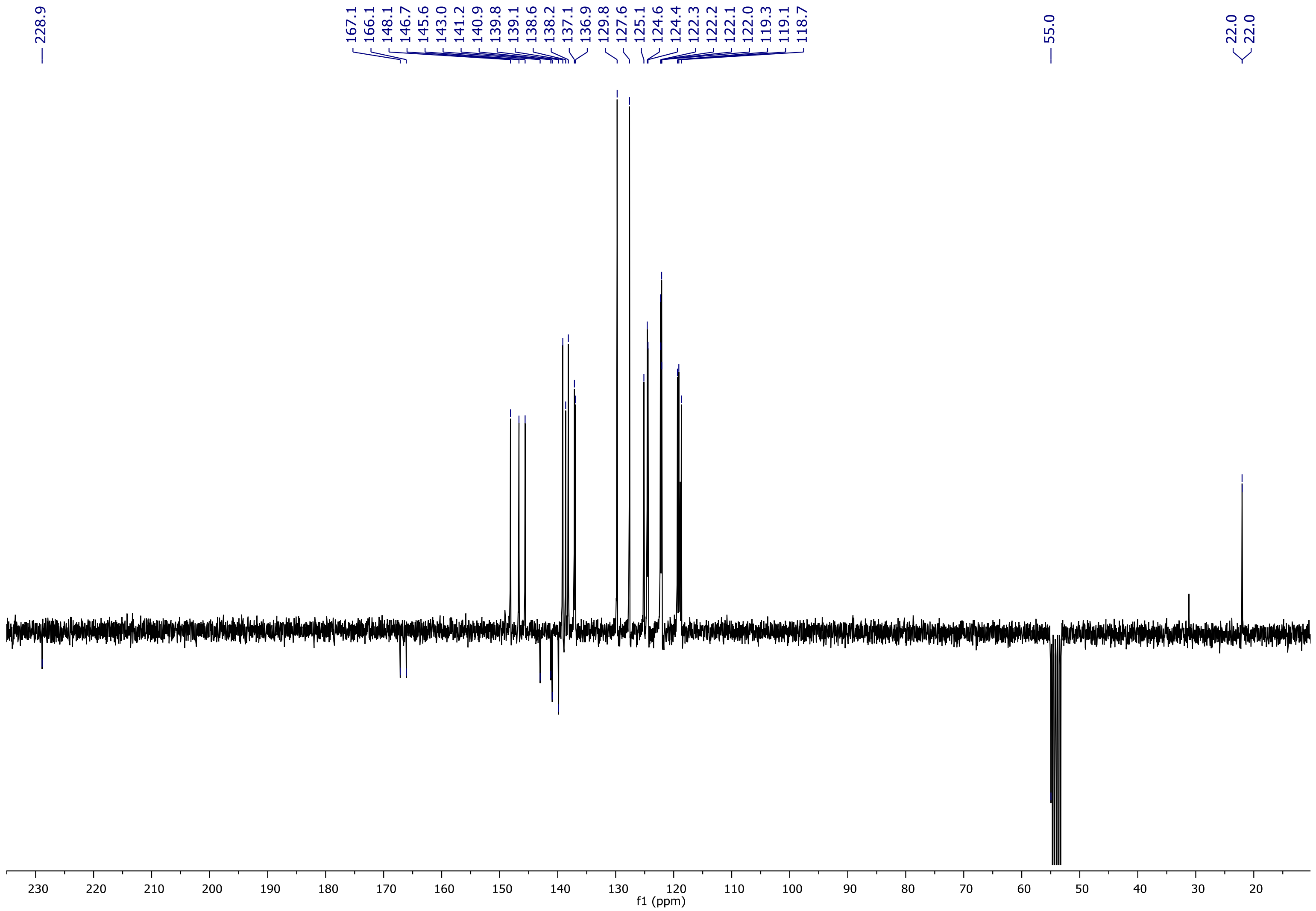

Figure S39. $^{13}C\{^{1}H\}$-APT NMR spectrum (75 MHz, CD_2Cl_2, 298 K) of complex **12.**

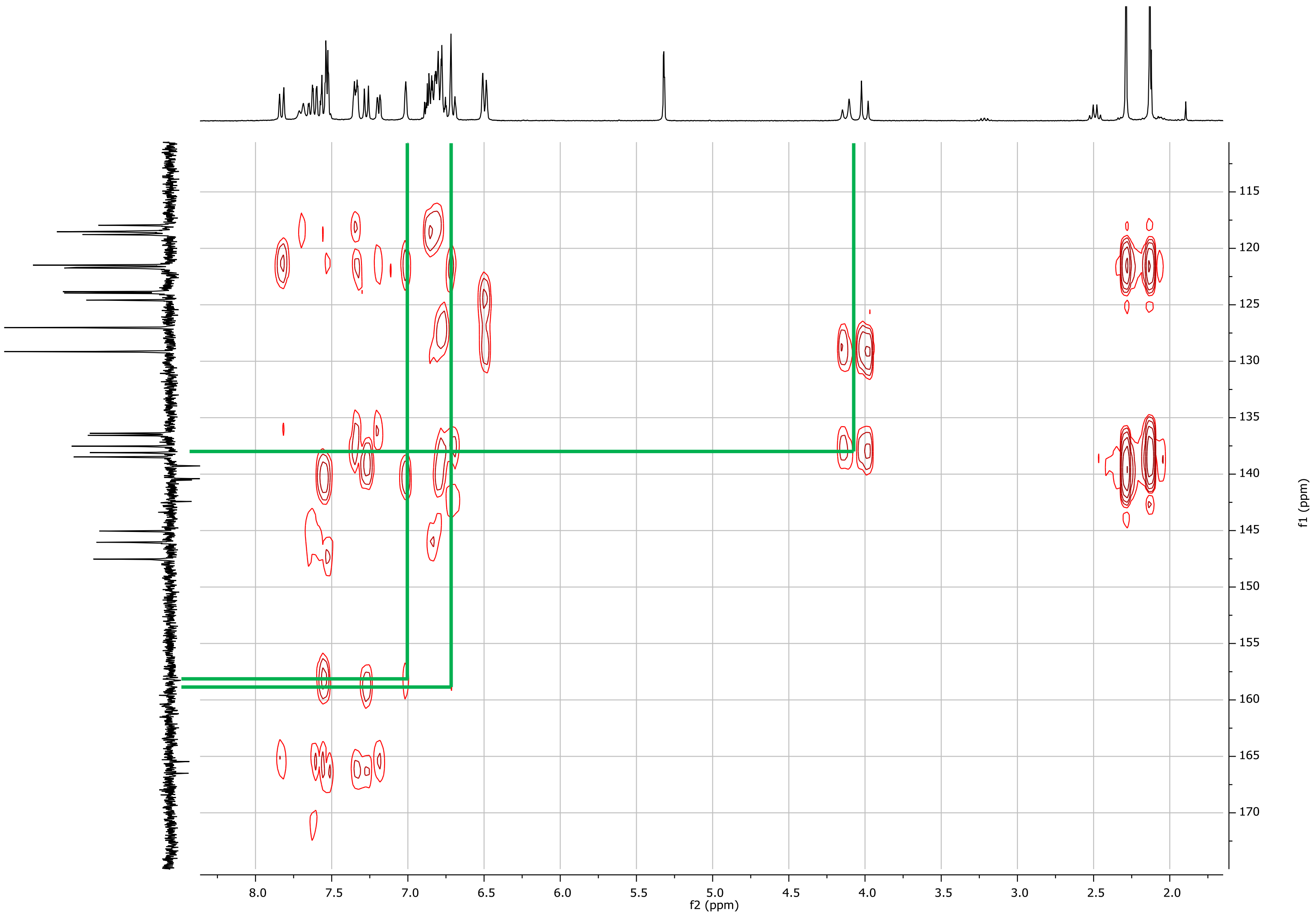

Figure S40. HMBC ^{1}H-$^{13}C\{^{1}H\}$ NMR spectrum (75 MHz, CD_2Cl_2, 298 K) of complex **12.**

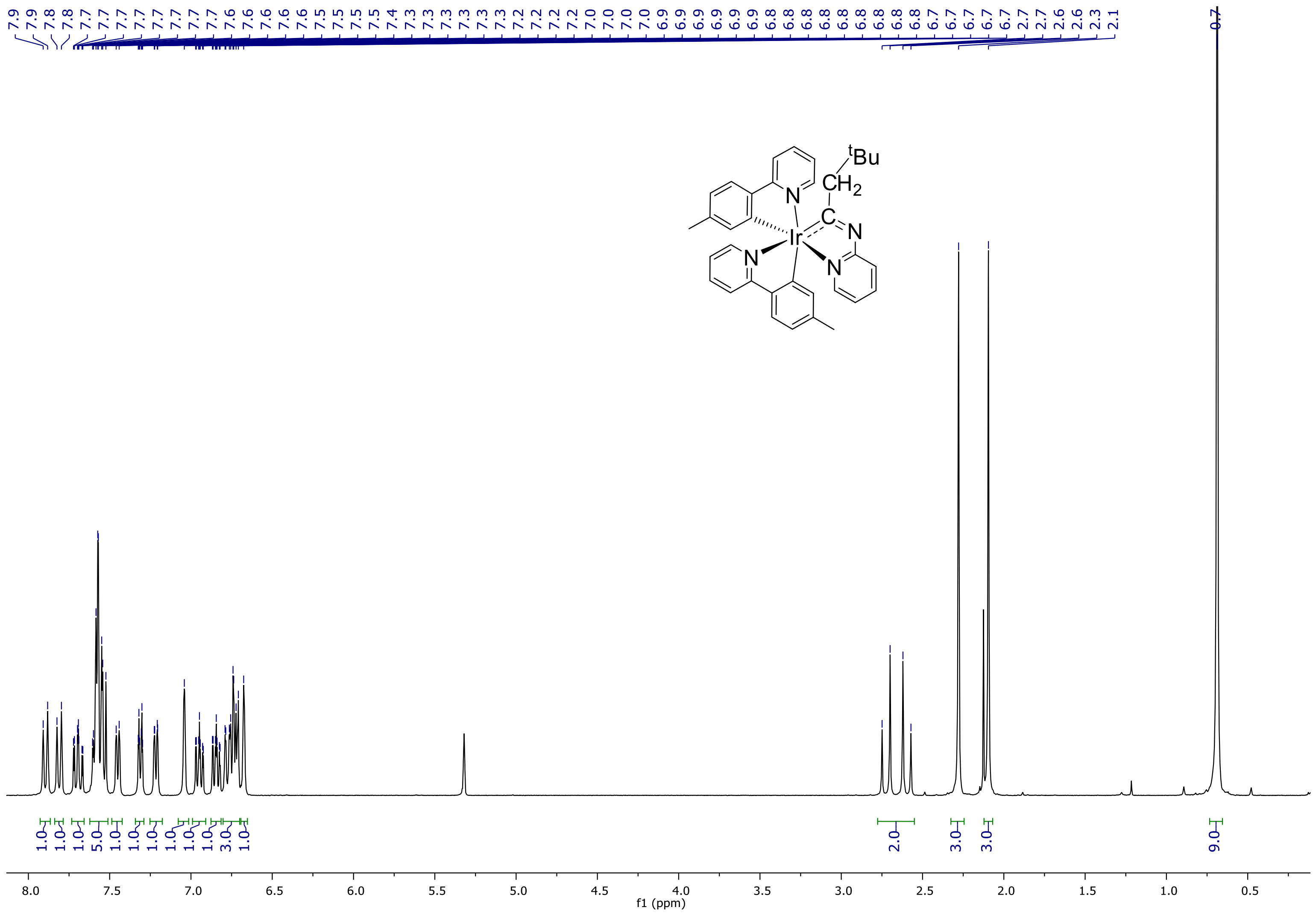

Figure S41. ^{1}H NMR spectrum (300 MHz, CD_2Cl_2, 298 K) of complex **13.**

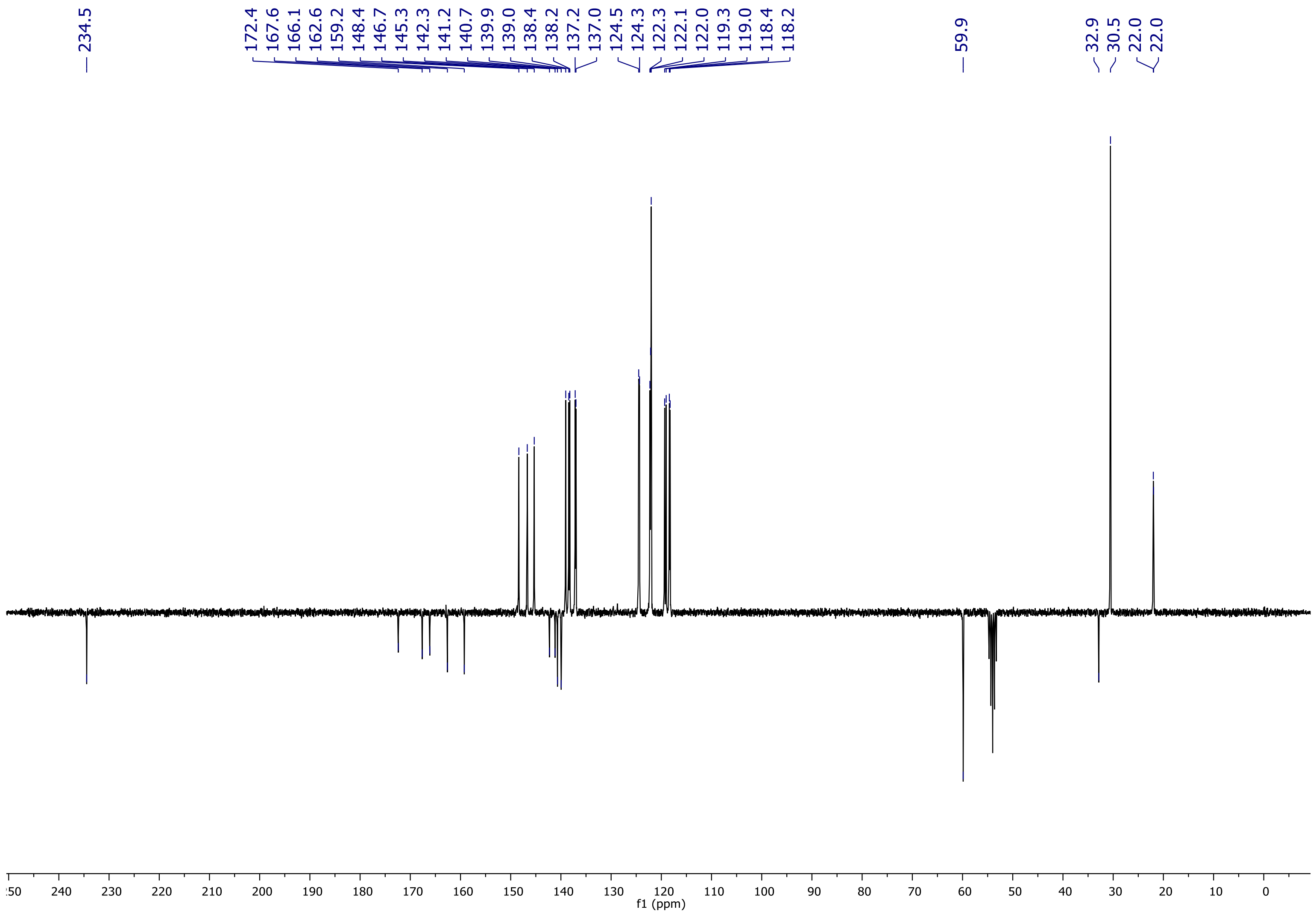

Figure S42. $^{13}C\{^{1}H\}$-APT NMR spectrum (75 MHz, CD_2Cl_2, 298 K) of complex **13.**

References

(1) Blessing, R. H. *Acta Crystallogr.* **1995**, *A51*, 33. SADABS: Area-detector absorption correction; Bruker- AXS, Madison, WI, 1996.

(2) SHELXL-2016/6. Sheldrick, G. M. *Acta Cryst.* **2008**, *A64*, 112-122.

(3) (a) Lee, C.; Yang, W.; Parr, R. G. Development of the Colle-Salvetti correlationenergy formula into a functional of the electron density. Phys. Rev. B 1988, 37, 785– 789. (b) Becke, A. D. Density-functional exchange-energy approximation with correct asymptotic behavior. J. Chem. Phys. 1993, 98, 5648–5652. (c) Stephens, P. J.; Devlin, F. J.; Chabalowski, C. F.; Frisch, M. J. Ab Initio Calculation of Vibrational Absorption and Circular Dichroism Spectra Using Density Functional Force Fields. J. Phys. Chem. 1994, 98, 11623–11627.

(4) Grimme, S.; Antony, J.; Ehrlich, S.; Krieg, H. A consistent and accurate ab initio parametrization of density functional dispersion correction (DFT-D) for the 94 elements H-Pu. J. Chem. Phys. 2010, 132, 154104.

(5) Gaussian 09, Revision D.01, Frisch, M. J.; Trucks, G. W.; Schlegel H. B.; Scuseria, G. E.; Robb, M. A.; Cheeseman, J. R.; Scalmani, G.; Barone, V.; Mennucci, B.; Petersson, G. A.; Nakatsuji, H.; Caricato, M.; Li, X.; Hratchian, H. P.; Izmaylov, A. F.; Bloino, J.; Zheng, G.; Sonnenberg, J. L.; Hada, M.; Ehara, M.; Toyota, K.; Fukuda, R.; Hasegawa, J.; Ishida, M.; Nakajima, T.; Honda, Y.; Kitao, O.; Nakai, H.; Vreven, T.; Montgomery, J. A.; Peralta, Jr., J. E.; Ogliaro, F.; Bearpark, M.; Heyd, J. J.; Brothers, E.; Kudin, K. N.; Staroverov, V. N.; Keith, T.; Kobayashi, R.; Normand, J.; Raghavachari, K.; Rendell, A.; Burant, J. C.; Iyengar, S. S.; Tomasi, J.; Cossi, M.; Rega, N.; S43 Millam, J. M.; Klene, M.; Knox, J. E.; Cross, J. B.; Bakken, V.; Adamo, C.; Jaramillo, J.; Gomperts, R.; Stratmann, R. E.; Yazyev, O.; Austin, A. J.; Cammi, R.; Pomelli, C.;

Ochterski, J. W.; Martin, R. L.; Morokuma, K.; Zakrzewski, V. G.; Voth, G. A.; Salvador, P.; Dannenberg, J. J.; Dapprich, S.; Daniels, A. D.; Farkas, O.; Foresman, J. B.; Ortiz, J. V.; Cioslowski, J.; Fox, D. J. Gaussian, Inc., Wallingford CT, 2013.

(6) Andrea, D.; Häußermann, U. M.; Dolg, M.; Stoll, H.; Preuss, H. Energyadjusted ab initio pseudopotentials for the second and third row transition elements. Theor. Chim. Acta 1990, 77, 123–141.

(7) Ehlers, A. W.; Bohme, M.; Dapprich, S.; Gobbi, A.; Hollwarth, A.; Jonas, V.; Kohler, K. F.; Stegmann, R.; Veldkamp, A.; Frenking, G. A set of f-polarization functions for pseudo-potential basis sets of the transition metals SC-Cu, Y-Ag and La-Au. Chem. Phys. Lett. 1993, 208, 111–114.

(8) (a) Hehre, W. J.; Ditchfield, R.; Pople, J. A. Self-Consistent Molecular Orbital Methods. XII. Further Extensions of Gaussian-Type Basis Sets for Use in Molecular Orbital Studies of Organic Molecules. J. Chem. Phys. 1972, 56, 2257–2261. (b) Francl, M. M.; Pietro, W. J.; Hehre, W. J.; Binkley, J. S.; Gordon, M. S.; DeFrees, D. J.; Pople, J. A. Self-consistent molecular orbital methods. XXIII. A polarization-type basis set for second-row elements. J. Chem. Phys. 1982, 77, 3654–3665.

(9) Marenich, A. V.; Cramer, C. J.; Truhlar, D. G. Universal Solvation Model Based on Solute Electron Density and on a Continuum Model of the Solvent Defined by the Bulk Dielectric Constant and Atomic Surface Tensions. J. Phys. Chem. B 2009, 113, 6378–6396.

(10) O'Boyle, N. M.; Tenderholt, A. L.; Langner, K. M. cclib: A Library for Package-Independent Computational Chemistry Algorithms. J. Comput. Chem. 2008, 29, 839−845.
